# A lineage-resolved cartography of microRNA promoter activity in *C. elegans* empowers multidimensional developmental analysis

**DOI:** 10.1038/s41467-024-47055-4

**Published:** 2024-03-30

**Authors:** Weina Xu, Jinyi Liu, Huan Qi, Ruolin Si, Zhiguang Zhao, Zhiju Tao, Yuchuan Bai, Shipeng Hu, Xiaohan Sun, Yulin Cong, Haoye Zhang, Duchangjiang Fan, Long Xiao, Yangyang Wang, Yongbin Li, Zhuo Du

**Affiliations:** 1 State Key Laboratory of Molecular Developmental Biology, Institute of Genetics and Developmental Biology, Chinese Academy of Sciences, Beijing, China; 2 University of Chinese Academy of Sciences, Beijing, China; 3 College of Life Sciences, Capital Normal University, Beijing, China

**Keywords:** Embryology, Differentiation, miRNAs, Single-cell imaging

## Abstract

Elucidating the expression of microRNAs in developing single cells is critical for functional discovery. Here, we construct scCAMERA (single-cell cartography of microRNA expression based on reporter assay), utilizing promoter-driven fluorescent reporters in conjunction with imaging and lineage tracing. The cartography delineates the transcriptional activity of 54 conserved microRNAs in lineage-resolved single cells throughout *C. elegans* embryogenesis. The combinatorial expression of microRNAs partitions cells into fine clusters reflecting their function and anatomy. Notably, the expression of individual microRNAs exhibits high cell specificity and divergence among family members. Guided by cellular expression patterns, we identify developmental functions of specific microRNAs, including miR-1 in pharynx development and physiology, miR-232 in excretory canal morphogenesis by repressing NHR-25/NR5A, and a functional synergy between miR-232 and miR-234 in canal development, demonstrating the broad utility of scCAMERA. Furthermore, integrative analysis reveals that tissue-specific fate determinants activate microRNAs to repress protein production from leaky transcripts associated with alternative, especially neuronal, fates, thereby enhancing the fidelity of developmental fate differentiation. Collectively, our study offers rich opportunities for multidimensional expression-informed analysis of microRNA biology in metazoans.

## Abstract (web summary)

By tracing promoter expression in lineage-mapped single cells, Xu et al. present a whole-body cartography of microRNA transcriptional activities during *C. elegans* embryogenesis and demonstrate its broad utility in multifaceted functional analyses.

## Introduction

MicroRNAs (miRNAs) are key non-coding RNAs playing prominent roles in post-transcriptional gene repression by pairing to complementary sites in the 3′ untranslated region (3′UTR) of target genes^1,2^. While genetic analyses have shown that miRNAs collectively play essential roles in organismal development and viability^3–6^, individual miRNA knockouts often lead to mild phenotypes^7–11^. Various explanations for the subtle effects of individual miRNAs have been proposed, including functional redundancy, regulation of the robustness rather than the execution of developmental processes, functioning under stress conditions, and regulation of highly specific cellular processes missed in general phenotypic assays^11,12^. Knowing the expression of each miRNA in individual developing cells can greatly aid analysis by providing insights into cellular and developmental contexts, guiding the design of synthetic genetic analyses, and prioritizing detailed phenotypic assays.

Since the discovery of miRNAs, their spatiotemporal expression has been extensively studied using techniques such as Northern blot, in situ hybridization, promoter-driven fluorescent reporters, and sequencing methods^13–21^. These studies, primarily conducted at the organismal or tissue levels, have revealed that miRNA expression is spatiotemporally specific, providing guidance for dissecting miRNA function during development. For instance, studies have employed methods like tissue-specific isolation or labeling of miRNAs followed by sequencing to characterize miRNAs enriched in specific tissues of interest in model organisms^13,17,20,22–24^. Although recent advances in single-cell genomics enable sequencing of protein-coding genes in developing cells at a single-cell resolution^25^, a similar analysis for miRNAs faces technical challenges in high-throughput sequencing of miRNAs in single cells and accurately determining cell identities. Due to their short length and lack of a poly-A tail, most commercially available single-cell RNA-seq platforms designed for protein-coding genes cannot be applied to miRNAs, despite the development of several single-cell miRNA-sequencing methods^26–28^. Moreover, inferring cell identities requires evaluating the expression status of multiple cell- or lineage-specific protein-coding genes^25^, necessitating simultaneous sequencing of both miRNAs and protein-coding genes. Consequently, a lineage-resolved single-cell atlas of miRNA expression has not been constructed using sequencing approaches.

An alternative method for determining gene expression in identity-resolved single cells involves generating promoter-fusion or protein-fusion (including endogenous labeling) fluorescent reporters, followed by high-resolution imaging and cell annotation. This approach has been applied in model organisms with simplified body plans, such as *C. elegans*, to map the cellular expression of specific genes^29–33^. With the advancement of the CRISPR/Cas9 technique, while tagging endogenous proteins with a fluorescent protein is feasible, it cannot be directly applied to miRNAs. A possible strategy involves replacing the miRNA locus with a fluorescent protein gene to monitor endogenous transcriptional activity^34^. However, it remains unclear whether the expression recapitulates that of the endogenous locus under miRNA-perturbed conditions. Therefore, although the approach based on the promoter-driven reporter has the limitation of missing critical cis-elements of transcription, it remains a widely used method to profile the transcriptional activity of miRNAs^18,35–39^. However, a systematic single-cell analysis of promoter-driven miRNA reporters has yet to be conducted, though it has been done for specific miRNAs such as miR-57^40^.

By utilizing promoter-driven reporters in conjunction with live imaging and systematic lineage tracing, we present single-cell CArtography of MicroRNA Expression based on Reporter Assay (scCAMERA) in *C. elegans*. It maps transcriptional activity of miRNA promoters throughout embryogenesis, offering a whole-body spatiotemporal miRNA expression atlas in metazoan species, notable for its resolution, annotation clarity, and extensive cell coverage. Through integrative analysis of miRNA expression in single-cell developmental contexts, we demonstrate that scCAMERA enables the discovery of functions of individual miRNAs and the dissection of general principles of miRNA-dependent developmental regulation.

## Results

### Construction of scCAMERA

**Fig. 1:**
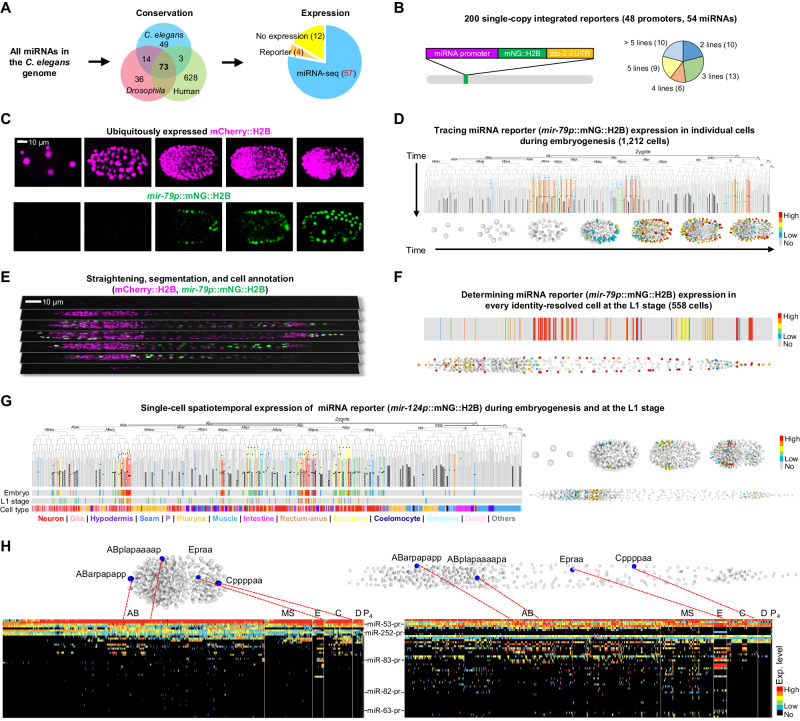
Construction of scCAMERA throughout embryogenesis. **A** Key steps in selecting miRNAs. **B** Left: Structure of miRNA-pr. Right: Distribution of the number of independent reporter strains generated for each miRNA. **C** 3D time-lapse imaging of embryogenesis using a dual-fluorescent reporter. Images show the embryonic expression of histone::mCherry and miRNA promoter-driven mNG::H2B at representative stages. **D** Top: Tree visualization of miRNA-pr expression mapped on the embryonic cell lineage leading to all 671 terminal cells. Vertical lines indicate cells, horizontal lines indicate cell divisions, dashed lines denote cells undergoing programmed cell death, and black lines denote late embryonic cells not covered by lineage tracing. Black dots represent cells with increased fluorescence intensity. Bottom: 3D rendering of the locations of all cells and their miRNA-pr expression levels at representative stages. **E** Expression of a miRNA-pr at the L1 stage after image straightening. **F** Top: Expression levels of a miRNA-pr in 558 identity-resolved cells at the L1 stage. Bottom: 3D rendering of cell locations and miRNA-pr expression levels. **G** Single-cell spatiotemporal expression of a miRNA reporter. The tree summary of the single-cell expression follows the organization shown in (**D**), with the first barcode located beneath the tree indicating the averaged expression level across all expressed cells in each cell track to capture developmental expression. A cell track is a sequence of temporally ordered mother-daughter cells leading to the corresponding terminal cell (Supplementary Fig. 1D). The second barcode illustrates cellular expression levels at the L1 stage. The third barcode denotes the tissue type of each cell. The 3D renderings on the right visualize the location and expression levels of miRNA-pr-expressing cells in embryos and at the L1 stage. **H** Cell-by-cell integration of multiple miRNA-pr expression in embryonic (left) and L1 stages (right). Shown above are the locations of representative cells in the embryo or at the L1 stage, with their names indicated. Shown below is a heatmap summary of the expression level of each miRNA reporter in each cell, ordered first by lineage and then by generation.

We selected 61 miRNAs as the targets from all *C. elegans* miRNAs documented in the miRBase^41^. These miRNAs have been identified as conserved among *C. elegans*, *D. melanogaster*, and *H. sapiens*^42^, and they are expressed either during embryogenesis or in the L1 stage^18,43–45^ (Fig. 1A and Methods). To profile their expression, we constructed reporters for 54 of the 61 miRNAs using their promoters (median length = 2.0 kb) to drive the expression of a nucleus-localized fluorescent protein mNeonGreen (mNG::H2B, Fig. 1B). We excluded miRNAs whose promoters are located within introns and transcribed in the same direction as the host gene unless they had been previously studied (Supplementary Fig. 1A). Each reporter was integrated into the genome as a single copy^46^, and two or more independent integration strains were generated for each miRNA to minimize potential expression bias caused by the integration site (Fig. 1B, Supplementary Fig. 1B for representative examples and Supplementary Data 1, 2). This process resulted in a collection of 200 transgenic strains.

Each reporter strain also ubiquitously expressed the histone::mCherry transgene, allowing visualization of all nuclei and facilitating single-cell expression mapping. Using these dual-reporter strains, we performed imaging-based single-cell identification, annotation, and lineage tracing to quantify the transcriptional activity of miRNA promoter-driven reporter (miRNA-pr) in lineage-resolved cells. 3D time-lapse imaging was performed to record embryogenesis with high spatiotemporal resolution (Fig. 1C), and the images were processed to identify and trace all cells for de novo construction of lineages up to the bean-to-comma stage (Fig. 1D)^31,47,48^. At this stage, the embryo undergoes terminal development, giving rise to 90% of all cells generated during embryogenesis. After cell identification, mNG::H2B intensity was quantified and adjusted to indicate the transcriptional activity of each miRNA-pr^30,31^, producing a cell-level expression pattern mapped onto the cell lineage (Fig. 1D and Supplementary Data 3).

To document expression during late embryogenesis that was not captured by lineage tracing, we annotated lineage identities of all 558 cells at the L1 stage as previously described (Fig. 1E, F)^32,49,50^. Due to the potential long half-life of mNG::H2B, the fluorescence intensity represents cumulative transcriptional activity over time. To further capture the temporal dynamics of miRNA-pr during embryogenesis, we assessed whether the fluorescence intensity increased within all traced time points in each cell (Methods and Supplementary Fig. 1C). The cells with increased expression, along with the earliest expressing cells observed throughout, allowed us to predict cells where a miRNA promoter is potentially active (Fig. 1D and Supplementary Data 3). Together, we quantified the transcriptional activity of 54 conserved miRNAs in every lineage-resolved cell throughout embryogenesis (Fig. 1G and Supplementary Fig. 1D).

*C. elegans* development follows an invariant cell lineage, generating a fixed number of terminal cells per individual, with each cell’s ultimate fate matching its lineage identity^51^. Therefore, by determining cell lineage identity, equivalent cells can be reliably identified among embryos, and various characteristics can be integrated and compared cell-by-cell. Exploiting this feature, we integrated the expression data from lineage-equivalent cells to generate the scCAMERA detailing the transcriptional activity of each miRNA-pr in each cell (Fig. 1H). When multiple reporter strains existed for the same miRNA, the reporter exhibiting the most representative expression pattern was determined and used (Methods and Supplementary Fig. 1E–G). Since the 3D position of each nucleus was followed, the scCAMERA connects miRNA expression to spatiotemporal cellular contexts in situ across embryonic development (Supplementary Data 3, https://dulab.genetics.ac.cn/scCAMERA).

**Fig. 2:**
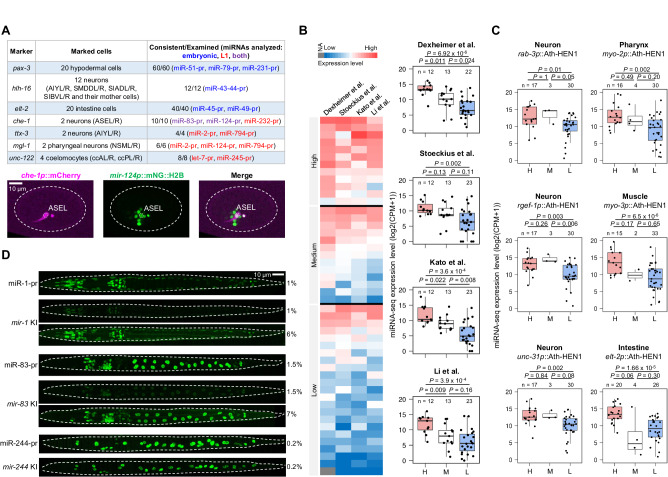
Quality assessment of scCAMERA. **A** Top: Summary of the accuracy of cell annotation clarity using cell-specific reporters. Bottom: An example illustrating the overlap of a reporter known to be expressed in the ASEL cell with *mir-124p*::mNG::H2B annotated as expressed in the ASEL cell. **B** Comparison of miRNA reporter expression with global miRNA-seq results. Left: Heatmap showing the relative expression of each miRNA analyzed in this study versus four previous studies using miRNA-seq. miRNAs are classified as high, medium, and low expression based on the number of miRNA-pr expression cells observed in this study. Right: Comparison of miRNA-seq expression values among miRNAs classified into high (H), medium (M), and low (L) expression in corresponding miRNA-seq datasets. The data are summarized as boxplots with boxes indicating the inter-quartile range (IQR), whiskers showing the range of values within 1.5*IQR, and horizontal lines indicating medians. Statistics: Mann–Whitney *U* test, two-tailed. **C** Comparison of miRNA reporter expression with tissue-level miRNA-seq data. Data organization and statistics remain consistent with those presented in (**B**). **D** Comparison of cellular fluorescence intensity of miRNA reporters with endogenous fluorescent knockin (KI) reporters. Each image is a maximum projection of 3D fluorescence images acquired using the laser power (expressed as a percentage of the maximum) listed on the right. Source data are provided as a Source Data file.

A series of quality control measures confirmed the general reliability of the scCAMERA. First, expression quantification was reproducible, producing Pearson correlation coefficients of 0.86 and 0.95 at the strain and cellular levels, respectively (Supplementary Fig. 2). Second, using seven cell-specific markers^52–58^, we verified the high accuracy of cell identity annotation at the embryonic and L1 stage. In all 140 examined cases involving 13 miRNAs, the expression status of miRNA reporters in marker-positive cells was consistent with that determined by lineage tracing or cell annotation (Fig. 2A and Supplementary Data 4). Third, over 84% of the leaf cells (terminal cells of the traced cell lineage) in which the miRNA-pr was predicted as active exhibited expression in the corresponding cells at the L1 stage (Supplementary Fig. 3). It indicates that the embryonic and L1 data are comparable despite being determined using different microscopy systems. Fourth, miRNA-pr expression was generally consistent with miRNA-seq results. We categorized miRNAs into three categories based on global and tissue-level miRNA-pr expression and compared corresponding miRNA-seq expression levels between them. Concordant results were observed for four global^3,44,45,59^ (Fig. 2B) and six tissue-level^13^ miRNA-seq datasets (Fig. 2C). Finally, we compared the expression of six miRNA reporters with endogenous fluorescent labeling, where the endogenous miRNA locus was replaced with mNG::H2B. Endogenous labeling generally resulted in significantly weak or no fluorescence signals (Fig. 2D and Supplementary Fig. 4A, B). Nevertheless, the endogenous expression patterns for three miRNAs with obvious fluorescence signals were highly similar to those of miRNA reporters (Fig. 2D, Supplementary Fig. 4C, and Supplementary Data 3).

### miRNA expression distinguishes cells by anatomy and function

**Fig. 3:**
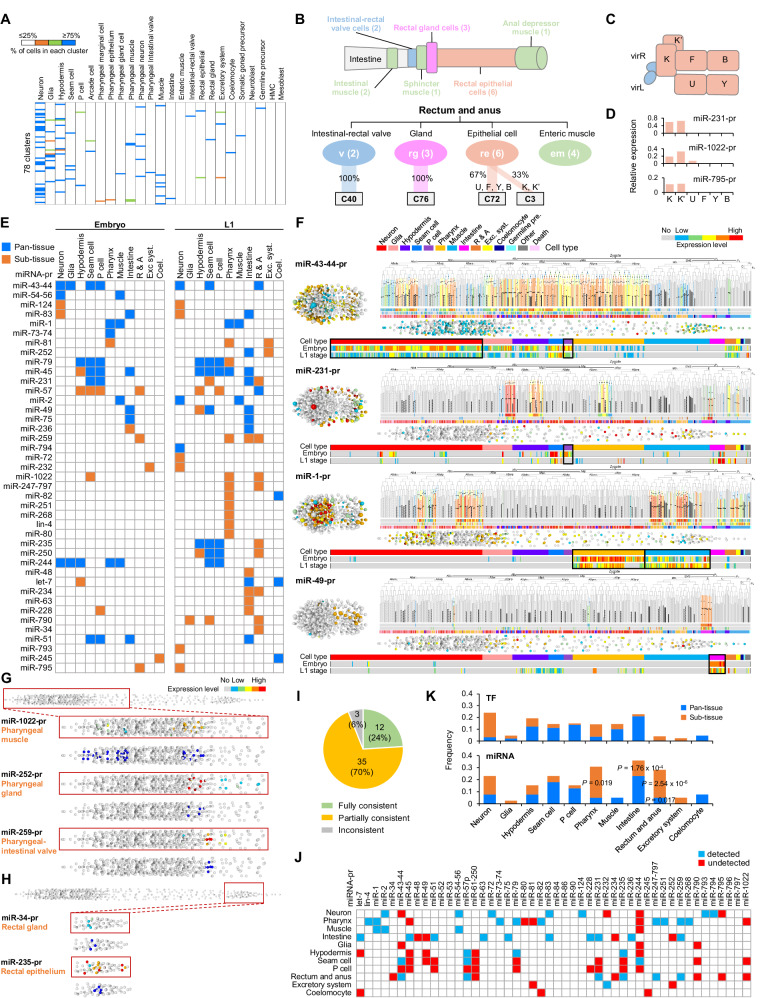
Fine patterning of cells by combinatorial expression of miRNAs. **A** Distribution of tissue types (columns) of cells in each cell cluster (row). Different colors indicate the percentage of cells in each cell cluster. Only cell clusters with more than one cell are shown. **B** Top: Schematic representation of the anatomy of the rectum-anus. Bottom: Cell cluster (box) distribution of cell types (oval) constituting the rectum-anus. Numbers in ovals indicate cell number, and the thickness of lines connecting ovals and boxes indicate the percentage of cells within the type that is classified into a given cluster. **C** Schematic representation of the position of six rectum epithelial cells relative to the intestinal-rectal valve. **D** Representative miRNA-prs that exhibited selective expression enrichment in K and K’ cells among all six epithelial cells. **E** Pan- and sub-tissue-specific miRNA-prs identified in embryonic and L1 stages. R & A indicates rectum-anus. **F** Single-cell, spatiotemporal expression of representative pan-tissue miRNA-prs. For each miRNA-pr, the expression visualization is identical to Fig. 1G. The set of barcodes at the bottom illustrates embryonic and L1 expression in cells ordered by tissue type, with the black box highlighting the enriched tissue type. 3D rendering showing sub-tissue-specificity of representative miRNAs in the pharynx (**G**) and rectum-anus (**H**) at the L1 stage. Blue highlights the cell location of a specific cell type (indicated on the left). **I** Summary of the results of comparing scCAMERA to the literature. **J** Heatmap illustrating the detection of tissue enrichment for miRNA-prs identified in this study, compared with findings from previous studies. **K** Frequency of each type of tissue- and sub-tissue-specific TF-pr (top) and miRNA-pr (bottom). Statistics: one-sided hypergeometric test.

Having constructed the scCAMERA, we sought to infer the implications of miRNAs for spatiotemporal fate patterning. Temporally, in line with a previous study^18^, miRNA tended to be activated in late embryos or at the L1 stage (Supplementary Fig. 5 and Supplementary Data 3). This suggests that miRNAs more frequently function in late developmental processes, such as terminal differentiation and tissue morphogenesis. De novo clustering of 558 terminal cells yielded cell clusters corresponding to tissue or cell types (hereafter referred to as tissue types for simplicity, Methods, Supplementary Fig. 6, and Supplementary Data 5). Manual classification of cells into 25 tissue types revealed that in 71 of the 78 (91%) cell clusters with more than one cell, the vast majority of cells (≥75%) were associated with a specific tissue (Fig. 3A and Supplementary Data 5). For example, 27 cell clusters consisted entirely of neurons and 6 were exclusively body wall muscle. Notably, miRNA-pr expression correctly segregated 9 out of 13 rare cell types (those with ≤6 cells) into a single cluster covering most (≥75%) of the cells (Supplementary Data 5). For instance, pharyngeal gland cells, coelomocytes, and somatic gonad precursors were all segregated into one cluster.

The combinatorial expression also distinguished the cells of complex tissues into clusters reflecting their anatomic or functional organization. For example, cells that form the rectum-anus were classified into four clusters according to anatomic and functional characteristics (Fig. 3B). The rectum-anus consists of four cell types: intestine-rectal valve cells that connect the rectum to the intestine, rectal gland cells that secrete digestive enzymes, rectal epithelial cells, and enteric muscle cells that function during defecation^60^. Consistent with this cell composition, except for enteric muscle, all cell types were correctly classified into distinct clusters (Fig. 3B). Interestingly, the rectal epithelial cells were divided into two clusters associated with cell anatomy (Fig. 3C). The anterior cells, K and K’, connect valve cells and are sorted into one cluster, while the other four cells cluster into a separate group. Accordingly, the K and K’ cells were distinctly enriched for the expression of several miRNAs, such as miR-231, miR-1022, and miR-795 (Fig. 3D).

The above results demonstrate that combinatorial expression of a small number of miRNAs is informative for patterning individual terminal cells into distinct classes that accurately reflect the anatomical and functional organization of cell types.

### miRNA expression exhibits high cell specificity and intra-family divergence

Next, we determined the expression specificity of individual miRNAs (Supplementary Data 6). Consistent with previous findings^14,18,19^, more than 80% of miRNA-prs exhibited significant expression enrichment in one or more tissues (tissue-specific miRNA, Fig. 3E). The single-cell resolution of our data allowed a detailed characterization of the cellular context and cell coverage of expression. Some tissue-specific miRNAs were expressed in the vast majority (>70%) of cells within a tissue (termed pan-tissue-specific miRNAs). Examples include the pan-neuron (miR-43-44), pan-seam cell (miR-43-44 and miR-231), pan-pharynx (miR-1), pan-muscle (miR-1), and pan-intestine (miR-49) (Fig. 3F). However, many were expressed only in a subset of cells within a tissue, and hence are termed sub-tissue-specific miRNAs (Fig. 3E). Notably, many were restricted to a particular cell type; for instance, preferential expression of miR-1022, miR-252, and miR-259 in pharyngeal muscle, gland, and pharyngeal-intestinal valve cells, respectively (Fig. 3G). Similarly, miR-34 and miR-235 reporters were preferentially expressed in the rectal gland and epithelium (Fig. 3H). Fate-transformation experiments on progenitors further verified that the expression of tissue-specific miRNAs was coupled to developmental fate since miRNA expression changed accordingly when cell fate was switched (Methods and Supplementary Fig. 7).

A systematic comparison of the enrichment patterns with previous studies, including those utilizing similar promoter-driven reporters or employing endogenous approaches such as tissue-level miRNA-seq, revealed that over 90% of patterns were partially consistent with previous findings^13,15,18,37,40,43,61–69^ (Methods, Fig. 3I, and Supplementary Data 7), confirming the biological relevance of the atlas. Notably, a considerable portion (55.3%) of the tissue-specific patterns were not previously documented (Fig. 3J, Supplementary Fig. 8, and Supplementary Data 8), thus highlighting the utility of cellular resolution expression analysis.

Interestingly, a few specific tissues accounted for a large fraction of sub-tissue miRNAs, particularly the neuron, pharynx, intestine, and rectum-anus; this was mainly evident at the L1 stage (Fig. 3K). For example, as much as 26% of miRNA-prs exhibited sub-pharynx-specific expression. To exclude the possibility that this expression pattern is generally found for regulatory genes rather than being miRNA-promoter specific, we calculated the distribution of tissue-specific transcription factor (TF) reporters, another regulatory gene category. As a relevant comparison, we used a single-cell expression atlas at the L1 stage, also constructed by the same method using promoter-driven fluorescent reporters (Supplementary Data 9)^50^. The result verified that sub-tissue specificity in the pharynx, intestine, and rectum-anus, collectively constituting the digestive system, are present at significantly higher frequencies than TFs (Fig. 3K). This analysis suggests that miRNAs might contribute to specific properties of the digestive system.

In addition to pervasive tissue-specific expression, miRNA-pr expression was highly restricted. Notably, 16 were restricted to less than 20 cells (Supplementary Fig. 9 and Supplementary Data 3). For example, miR-232 is expressed in a pair of sibling cells that develop into the RMEV motor neuron and excretory canal cell, miR-228 in a pair of left-right symmetric intestinal cells (int9L and int9R), and miR-795 in three neurons (AWAL, AWAR, ASIL). The late and highly restricted expression suggests that these miRNAs regulate context-specific cellular processes.

Different miRNAs that share a “seed” sequence (nucleotides 2–7) for target recognition are classified into the same family as they likely recognize similar target genes^70^. Previous tissue-level analyses show that miRNA family members tend to exhibit similar expression patterns, although specific differences are recognized^14,18,71^. However, evaluation at the single-cell level found most family members to exhibit highly divergent expression (Supplementary Fig. 10A). We compared the intra-family miRNA-pr expression divergences relative to those between inter-families and found that only 45% (embryos) and 27% (L1 stage) intra-family miRNA-prs displayed high similarity, defined as the similarity being ranked among the top 20% of corresponding inter-family comparisons (Supplementary Fig. 10B, C). This finding was further supported by the analysis of tissue-level miRNA-seq data^13^, where only 26% of intra-family miRNAs exhibited high expression similarity across tissues (Supplementary Fig. 10D). Such high expression divergence may enable a given miRNA family to regulate different targets in different contexts, likely increasing the functional repertoire of miRNAs.

Collectively, the single-cell annotation and whole-body cell coverage of the scCAMERA expand the catalog of tissue-specific miRNAs, provide precise cellular contexts for each miRNA, and reveal general properties of miRNA expression. Below, we present examples demonstrating the effectiveness of cellular-level expression in guiding the functional analysis of individual miRNAs.

### miR-1 modulates pharynx development and function

**Fig. 4:**
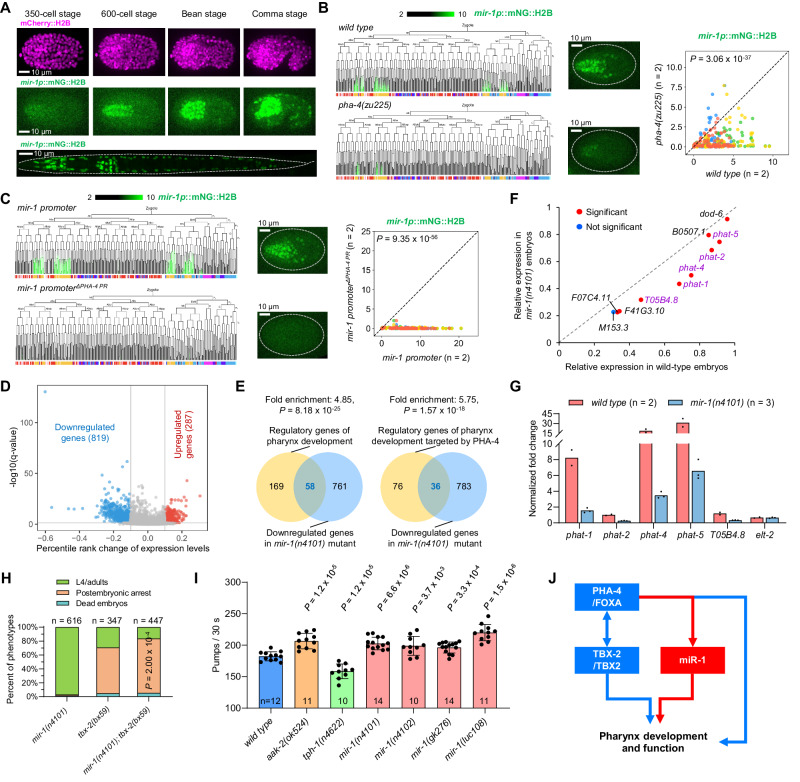
miR-1 functions in pharynx development and physiology. **A** Micrographs showing miR-1-pr expression during embryogenesis and at the L1 stage. **B** Left: Changes in miR-1-pr expression in *pha-4(zu225)* embryos at 20 °C. The barcode indicates cell type of each traced terminal cell with a color scheme identical to Fig. 3F. Middle: Micrographs comparing miR-1-pr expression at the 350-cell stage. Right: Comparison of miR-1-pr expression levels in equivalent cells (dots) of the *wild type* and *pha-4(zu225)* embryos. The dashed diagonal indicates equality of X and Y. Only wild-type/*mir-1* embryo pairs with an identical orientation were compared; different colors indicate different embryo pairs. Only cell tracks with both PHA-4 and miR-1-pr expression were included. Statistics: Wilcoxon rank sum test, two-tailed. **C** Comparison of miR-1-pr expression after removing the PHA-4-bound region. **D** Expression changes and *Q* values of genes exhibiting differential expression in *mir-1(n4101)* embryos. **E** Overlap of the set of downregulated genes in *mir-1(n4101)* with gene sets relating to pharynx development. Statistics: one-sided hypergeometric test. Peak regions of PHA-4 ChIP-seq from two sources were used to identify PHA-4 target genes^76,160^. **F** Changes in the expression of genes related to pharyngeal gland development. Purple indicates genes selected for qRT-PCR verification. **G** Quantitative RT-PCR comparing the expression levels of five selected gland-related genes and a negative-control gene (*elt-2*) relative to the *tba-1* gene between wild-type and *mir-1(n4101)* embryos. Each bar represents the mean value. **H** Comparison of embryonic and postembryonic arrest rates between single and double mutants of *mir-1* and *tbx-2*. L4 stage P0 animals were cultured at 25 °C, and their F1 progeny were subsequently utilized for analysis. The *P* value (two-tailed Chi-square test) compares the observed L1-L3 arrest rate to that predicted based on the additive effects of single mutants. **I** Comparison of pharynx pumping rates between *wild type*, *mir-1* mutants, and two known mutants that exhibit abnormal pumping rates at the young adult stage. Data are represented as mean ± SD. Statistics (Mann–Whitney *U* test, two-tailed) were performed between a mutant and the *wild type*. **J** Proposed action model of miR-1 in the pharynx. Source data are provided as a Source Data file.

The first case concerns the role of miR-1 in pharynx development. Consistent with previous findings^15,18^, miR-1 was identified as a pan-pharynx and pan-muscle miRNA (Fig. 3E). While the role of miR-1 in muscle physiology and synaptic transmission at neuromuscular junctions has been reported^36,72,73^, its function in pharynx development remains elusive, although phenotypic defects have been observed in pharyngeal muscle cells and pharynx pumping rate in *mir-1* mutants^73^. We found that pharyngeal expression of miR-1-pr was initiated at ~200-cell-stage embryos and covered 97% (92/95) of pharyngeal cells (Fig. 4A). Except for the pharyngeal neurons and gland cells, most expression signals persisted at the L1 stage (Supplementary Fig. 11A). Since pharyngeal expression of miR-1-pr is similar to but initiates later than the pharynx-fate specification TF PHA-4/FOXA1^74^, we determined whether PHA-4 activates miR-1. Previous generated Chromatin immunoprecipitation sequencing (ChIP-seq) data^75,76^ indicated that PHA-4 binds to the *mir-1* promoter (Supplementary Fig. 11B), and we further confirmed that pharyngeal expression of the miR-1-pr was significantly reduced in a *pha-4* mutant (Fig. 4B). In addition, removing the PHA-4-bound region (264 bp) from the promoter abolished pharyngeal expression of miR-1-pr (Methods and Fig. 4C), supporting an indispensable role of PHA-4 in activating miR-1 transcription.

We then examined the molecular function of miR-1. RNA-seq of a *mir-1* mutant identified 1,106 differentially expressed genes, including 819 downregulated and 287 upregulated (Fig. 4D and Supplementary Data 10); the entire locus was deleted in this allele, and no transcripts were detectable^8,20^. Functional analysis of the downregulated genes revealed significant enrichment of ontology terms related to pharynx development and function^77^ (Supplementary Fig. 11C) along with significant enrichment (4.85-fold) of genes previously defined as participating in pharynx development^74^, especially those also bound by PHA-4 (5.75-fold) (Fig. 4E). Notably, all detected genes previously defined as involved in pharyngeal gland morphogenesis were downregulated^78^ (Fig. 4F and Supplementary Data 10). We selected five gland-related genes highly expressed in wild-type embryos but significantly reduced in the mutant and validated reduced expression using quantitative RT-PCR (Fig. 4G and Supplementary Fig. 11D). Thus, PHA-4 activates miR-1 as a helper for activating its direct targets. Since miRNAs primarily inhibit translation or reduce mRNA stability, the downregulation of pharynx-related genes likely reflects an indirect role of miR-1.

We finally examined the developmental and physiological roles of miR-1. Lineage tracing of the *mir-1* mutant detected no discernible changes in the relative positions of pharyngeal cells and only mild changes in cell lineage pattern (Supplementary Fig. 11E–G and Supplementary Data 11), implying that miR-1 alone is not essential for pharyngeal lineage specification. However, we found miR-1 to function synergistically with a temperature-sensitive allele of *tbx-2*, a regulator of pharynx development that presumably acts as a repressor^79–81^. Incubated at the restrictive temperature, double mutants lacking *mir-1* and *tbx-2* caused significantly higher levels of larval arrest compared with that expected from additive effects of the single mutants (Fig. 4H). Moreover, double mutants exhibited a more severe pharynx morphological defect than expected, based on the size of the metacorpus relative to that of the terminal bulb (Supplementary Fig. 11H–J). These findings indicate that miR-1 interacts genetically with developmental regulators of the pharynx.

In addition, we found that miR-1 is required for pharynx function under normal feeding conditions. All tested *mir-1* mutants at the young adult stage exhibited an abnormally high pumping rate (Fig. 4I); meanwhile, expected increases and decreases were observed in well-characterized mutants employed as controls (*aak-2* for increased and *tph-1* for decreased pumping rate)^82^. This result contrasts with two recent studies, one reporting a decreased pharynx pumping rate in *mir-1* mutants compared to wild-type animals at the young adult stages^73^, and the other reporting an unaffected rate at Days 8 and 14 of adult stages^72^. Differences in developmental stage or methodology may explain the discrepancy. For example, the study reporting a decreased pumping rate treated immobilized animals with serotonin, while our study directly observed freely moving animals.

Together, we reveal that miR-1 functions in the PHA-4-dependent pathway in modulating pharyngeal development and function (Fig. 4J), demonstrating the utility of the expression information in discovering miRNA function.

### miR-232 inhibits NHR-25/Ftz-F1/NR5A to promote excretory canal morphogenesis

**Fig. 5:**
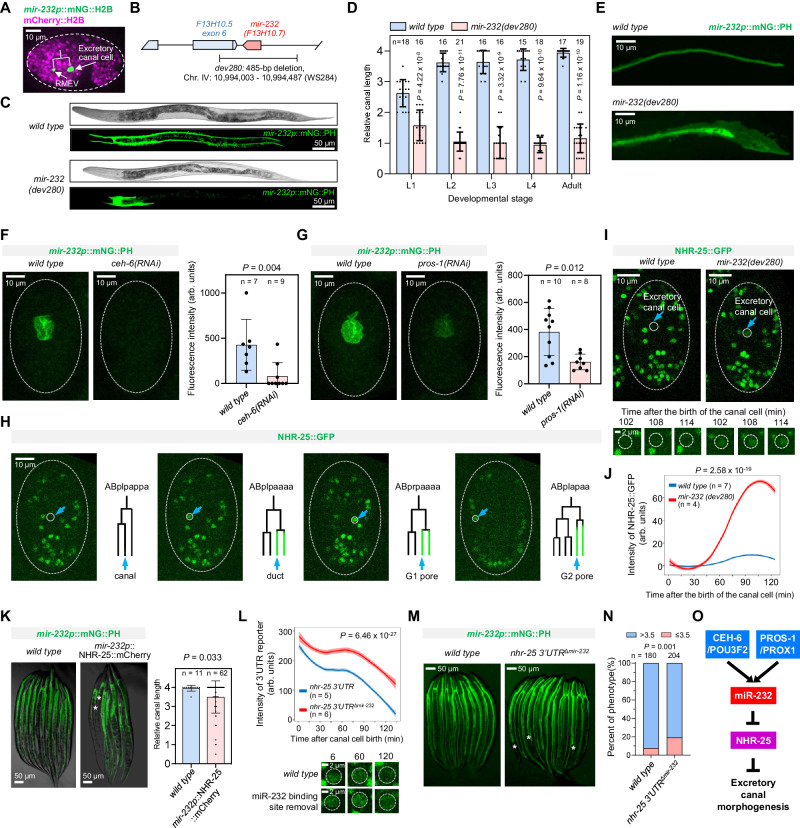
miR-232 regulates excretory canal morphogenesis by repressing NHR-25. **A** Expression of miR-232-pr. **B** *mir-232* deletion allele. **C** Comparison of excretory canal length (visualized by a membrane-localized mNG) between wild-type and *mir-232(dev280)* animals at the L4 stage. **D** Quantitative comparison of excretory canal length at postembryonic stages. Data are represented as mean ± SD. Statistics: Mann–Whitney *U* test, two-tailed. **E** Micrograph showing the cystic canal phenotype of the *mir-232* mutant. **F**, **G** Changes of miR-232 reporter expression upon RNAi against *ceh-6* and *pros-1*. Data are represented as mean ± SD. Statistics: Mann–Whitney *U* test, two-tailed. **H** Micrographs showing the expression of a protein-fusion reporter of NHR-25 in the four excretory cells with the epithelial identity. The cell lineage diagram on the right shows the corresponding cells’ developmental origin and NHR-25 expression status. **I** Top: Micrographs illustrating the difference in NHR-25 protein expression in the excretory canal cell between the wild-type and *mir-232(dev280)* embryos. Bottom: Enlarged views of NHR-25 expression in the canal cell. **J** Quantifying NHR-25::GFP expression in the excretory canal of wild-type and *mir-232(dev280)* embryos. Data are presented as mean ± 95% CI, Loess smoothed. Statistics: Wilcoxon rank sum test, two-tailed. **K** Left: Changes in canal length after ectopic expression of NHR-25 protein in the excretory canal at the L4 stage. Stars indicate animals with an abnormally short canal. Right: quantification of excretory canal length. Data are represented as mean ± SD. Statistics: Mann–Whitney *U* test, two-tailed. **L** Top: Quantifying mNG intensity expressed from the *nhr-25* 3′UTR reporter with or without miR-232 binding sites driven by a ubiquitous promoter (*ubq-1*). Data presentation and statistics are identical to (**J**). Bottom: mNG expression in the excretory canal at representative times. **M** Changes in excretory canal length at the L4 stage after mutating miR-232 binding sites in the endogenous *nhr-25* 3′UTR. Stars indicate animals with an abnormally short canal. **N** The proportion of animals with a shortened canal (≤3.5) after mutating miR-232 binding sites in the endogenous *nhr-25* 3′UTR. Statistics: Chi-square test, two-tailed. **O** Proposed function model of miR-232. Source data are provided as a Source Data file.

In the second case, we focused on miR-232, which exhibited cell-specific expression in the excretory canal cell (Fig. 5A). Expression of miR-232-pr was initiated late in embryogenesis in a pair of sibling cells: the RMEV neuron and the canal cell, with expression in the latter persisting to the L1 stage (Supplementary Fig. 12A, B). The canal cell develops into an H-shaped structure with two tubules that extend nearly the entire body length in wild-type animals^83–85^; along with the duct, pore, and gland cells, it forms the *C. elegans* excretory system, which functions in osmoregulation and is analogous to the vertebrate kidney.

We generated a deletion allele of *mir-232* and observed a significantly shortened canal across all postembryonic stages (Fig. 5B–D), with less than half the length in wild-type animals after the L1 stage. The mutant displayed defects at the canal cell terminus reminiscent of previously analyzed cystic canal mutants^84^ (Fig. 5E). Because the mutant also lacks part of exon 6 of the nearby gene *F13H10.5* (Fig. 5B), we performed a rescue experiment by injecting a fragment containing the entire *mir-232* locus; this rescued the short canal phenotype, suggesting that the defect was caused by loss of miR-232 (Supplementary Fig. 12C–E). In contrast, the development of the RMEV neuron in the *mir-232* mutant appeared normal, as judged by its length and morphology (Supplementary Fig. 12F, G). These results argue that miR-232 is required for canal cell morphogenesis.

Two homeobox TFs, PROS-1/PROX1, and CEH-6/POU3F2, are known to initiate canal cell differentiation and morphogenesis, and their loss results in canal cell shortening^86^. These proteins are respectively first observed in the mother and grandmother of the canal cell^31^, preceding the expression of miR-232-pr in the mother cell of the canal cell (Supplementary Fig. 12H). Knocking down either TF caused a loss or significant reduction of miR-232-pr expression in the canal cell (Fig. 5F, G), indicating that both TFs are required to activate miR-232.

Among the potential targets of miR-232, the nuclear receptor TF NHR-25 was of particular interest. *nhr-25* is the *C. elegans* ortholog of *Drosophila ftz-f1* and human *NR5A*, and is critical for epidermal differentiation and embryonic elongation^87,88^. Analysis of previous TF expression atlas showed that NHR-25 protein is expressed in all epithelial cells that constitute the excretory system, except the canal cell (Fig. 5H)^31^. We hypothesized that miR-232 might inhibit NHR-25 protein production in the canal cell to ensure normal morphogenesis. Four lines of evidence supported this hypothesis. First, the fluorescence intensity of an NHR-25::GFP protein-fusion reporter in the canal cell was significantly elevated in the *mir-232* mutant (Fig. 5I, J). Second, ectopic expression of NHR-25 in the canal cell led to a shortened excretory canal, albeit at a relatively low frequency (15%, 9/59, Fig. 5K). Third, a 3′UTR reporter assay validated that miR-232 binding sites inhibit mNG::H2B expression since reporter intensity in the canal cell significantly increased after their removal (Fig. 5L and Supplementary Fig. 12I). Finally, mutating two miR-232 binding sites in the endogenous *nhr-25* 3′UTR (*nhr-25 3*′*UTR*^*Δmir-232*^) induced a low-penetrant but reproducible shortening of the excretory canal (Fig. 5M, N and Supplementary Fig. 12J). Since the phenotype of *nhr-25 3*′*UTR*^*Δmir-232*^ was weaker than that of the *mir-232* mutant, additional factors function downstream of miR-232. Altogether, we have uncovered a miRNA-dependent pathway that regulates the development of the excretory system. PROS-1 and CEH-6 activate miR-232, which then functions in part through NHR-25 to ensure canal cell extension (Fig. 5O).

**Fig. 6:**
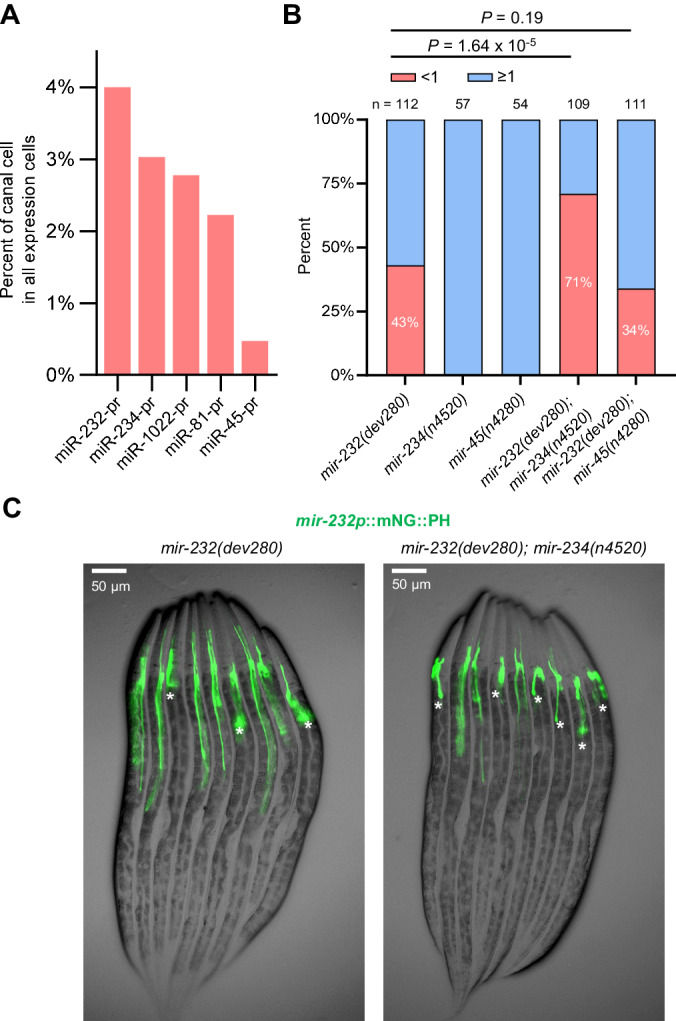
Genetic interaction between miR-232 and miR-234 in excretory canal development. **A** Top five miRNA-prs with expression enrichment in the excretory canal cell. The enrichment was calculated as the percentage of excretory canal cells among all miRNA-pr-expressing cells. **B** Comparison of the proportion of animals with a dramatically shortened canal (<1) between genotypes. Statistics: Chi-square test, two-tailed. **C** Micrographs showing excretory canal length in *mir-232* single mutants and *mir-232, mir-234* double mutants at the L4 stage. Stars indicate animals with a dramatically shortened canal. Source data are provided as a Source Data file.

In addition to miR-232, we identified several miRNA-prs exhibiting enriched expression in the excretory canal cells (Fig. 6A), suggesting additional miRNAs function synergistically with miR-232 to regulate canal morphogenesis. We focused on miR-234 and tested this possibility. Interestingly, while the *mir-234* mutant did not elicit canal shortening, double loss of *mir-232* and *mir-234* led to significantly more severe phenotypes than respective single mutants (Fig. 6B, C). As a control, we also examined miR-45, which was ubiquitously expressed in all six excretory cells, and observed no synergistic effects with miR-232 (Fig. 6B). This result suggests that miR-232 and miR-234 function in concert to regulate excretory canal elongation.

In conclusion, the above case studies demonstrate that the scCAMERA provides valuable guidance in dissecting the individual and combined role of miRNAs in normal development.

### Fate determinants activate miRNAs

Having characterized cellular expression features and developmental functions of individual miRNAs, we next explored the general scheme of miRNA-mediated developmental regulation. To begin, we examined the regulatory relationship between miRNAs and fate-determining TFs.

**Fig. 7:**
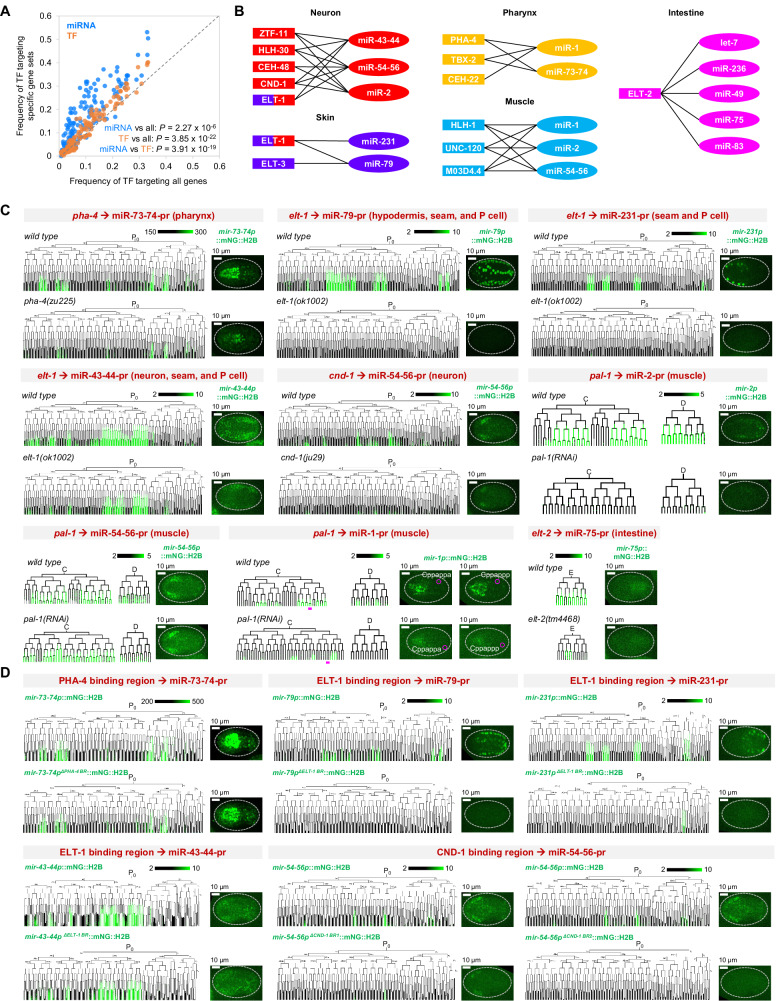
Fate determinants activate tissue-specific miRNAs. **A** Comparison of the frequency with which each TF (dot, *n* = 143) binds at the promoters of all protein-coding genes to that at the promoters of specific gene classes (Y-axis). The dashed diagonal indicates equality of X and Y. Statistics: Wilcoxon rank sum test, two-tailed. **B** Tissue fate determinants (box) frequently bind at (line) the promoters of tissue-specific miRNAs (oval) that are active in the same tissue (indicated by color). Two colors are assigned to ELT-1 because it is reported to function in specifying both skin and neuron fate^31,93^. **C** Changes in the expression of tissue-specific miRNAs after perturbing fate determinants for which ChIP-seq peaks were detected in the miRNA promoter. In each figure, the left shows the cell lineage expression of a miRNA reporter up to the 350-cell embryonic stage, before (top) and after (bottom) perturbing a fate determinant at 20 °C. Micrographs show the fluorescence intensity of miRNA reporters in bean-stage embryos. Magenta highlights the lineage and embryonic location of a pair of cells. **D** Changes in the expression of tissue-specific miRNAs after removing the regions bound by the indicated fate determinants from miRNA promoters. Figure organization is as in (**C**). In (**C**) and (**D**), results from one representative embryo are shown. Two embryos were analyzed, yielding similar results.

Using previously generated ChIP-seq data^75,76^, we found that TFs more frequently bound miRNA promoters (TF→miRNAp) than those of protein-coding genes and that the TF→miRNAp frequency even surpassed that of TF→TFp (Fig. 7A, Supplementary Fig. 13A, and Supplementary Data 12). Notably, we found that the promoters of tissue-specific miRNAs were significantly more frequently bound by TFs exhibiting the same tissue specificity than by other TFs (Supplementary Fig. 13B). Similarly, tissue-specific TFs, including fate determinants, significantly more frequently targeted promoters of miRNAs exhibiting the same tissue specificity than other miRNAs (Supplementary Fig. 13C). For example, miR-43-44, a neuron-specific miRNA, was bound by CND-1/NEUROD1, a motor neuron fate determinant^89,90^, while miR-2, a muscle-specific miRNA, was bound by HLH-1/MYF6 and HND-1/PTF1A, two critical TFs that specify body wall muscle fate^91^ (Fig. 7B, Supplementary Fig. 13D, and Supplementary Data 12). Together with the findings that PHA-4/FOXA1 activates miR-1 and PROS-1/PROX1 and CEH-6/POU3F2 activate miR-232, these data suggest that tissue fate-determining TFs preferentially target miRNAs during tissue differentiation.

To validate whether tissue fate determinants are required for miRNA expression, we focused on known determinants of five major tissue types: CND-1 (a subset of neurons)^89,90^; PHA-4 (pharynx)^74,92^; ELT-1 (skin)^93^; HLH-1, HND-1, and UNC-120 (muscle)^91^; and ELT-2 (intestine)^94^. We perturbed these TFs using loss-of-function alleles or RNAi and quantified in nine experiment sets the cellular expression of tissue-specific miRNAs. Because HLH-1, UNC-120, and HND-1 function redundantly^91^, we used RNAi against *pal-1*/*Caudal/CDX1*, a TF that functions upstream of all three, to disrupt the entire cascade and other cell differentiation programs in the C and D lineages^95,96^. In all but one experiment, miRNA-pr expression in all or a subset of the cells belonging to the corresponding tissues was abolished or significantly reduced (Fig. 7C, Supplementary Fig. 13E, and Supplementary Data 13). For example, neuronal expression of miR-54-56-pr was significantly reduced in the *cnd-1* mutant, and loss of *elt-1* resulted in complete loss of miR-79-pr and miR-231-pr expression. The only exception was miR-43-44-pr, whose expression was not reduced upon *cnd-1* loss (Supplementary Fig. 13F), suggesting that another factor or a combination of multiple factors is required for its activation. Both gain and loss of miR-54-56-pr expression were observed upon *pal-1* loss (Fig. 7C), likely reflecting complex regulation downstream of PAL-1^95^.

To further verify the above findings, we selected five determinant-miRNA pairs with considerable expression reduction and deleted the corresponding peak regions defined by ChIP-seq from the miRNA promoters. We generated single-copy integration lines of fluorescent reporters (Methods), both with and without the peak region, integrated into the identical genomic region on Chr V (*oxTi365*, 8.64 Mb). We found that in all but one case (*elt-1*→miR-43-44p), loss of peak regions corresponded to significant downregulation or loss of expression (Fig. 7D, Supplementary Fig. 13G, and Supplementary Data 14). It should be noted that different TFs might function redundantly or as a module to regulate miRNA promoter activity by binding at identical peak regions, a possibility that requires further validation.

Collectively, the above findings reveal a general regulatory scheme in which tissue-fate determinants act alone or in conjunction with other TFs to activate miRNAs during tissue differentiation.

### miRNAs increase differentiation fidelity by targeting leaky transcripts

We next explored the general function of miRNAs in cell differentiation by examining the expression and function of their predicted target genes across the cell lineage (Supplementary Data 15). Using the single-cell transcriptomic atlas of *C. elegans* embryogenesis^25^, we confirmed the previous findings that miRNAs and their targets were frequently co-expressed (Supplementary Fig. 14A), though the transcriptional levels of their targets were significantly lower in miRNA-pr-expressing cell tracks (Supplementary Fig. 14B)^97–100^. This finding validates that the miRNA-mediated repression of their targets reinforces the transcriptional program regulating the transcription of miRNA’s target, known as the coherent model^98^.

**Fig. 8:**
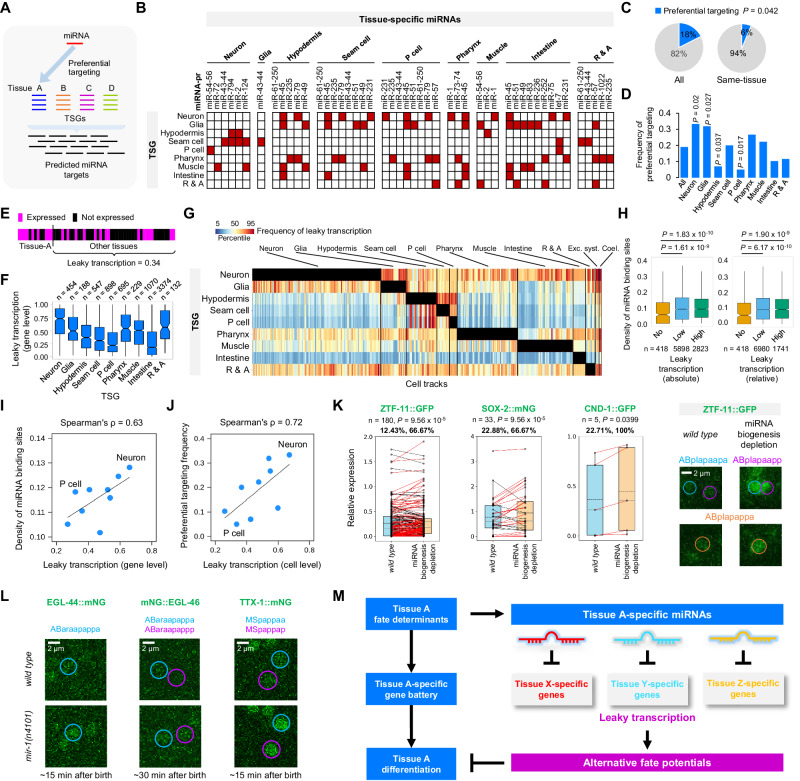
miRNAs target leaky transcripts to increase differentiation fidelity. **A** Preferential targeting of miRNAs against TSGs. **B** Preferential targeting of tissue-specific miRNAs against different types of TSGs. **C** Comparison of the expected and observed frequencies of same-tissue preferential targeting. Statistics: Fisher’s exact test, two-tailed. **D** Comparison of the frequencies of preferential targeting with which TSGs of the indicated tissue are targeted by miRNAs specific to other tissues. Statistics: Fisher’s exact test, two-tailed. **E** Quantification of leaky transcription. **F** Comparison of leaky transcription of TSGs. The data are summarized as boxplots with boxes indicating the IQR, whiskers showing the range of values within 1.5*IQR, and horizontal lines indicating medians. **G** Expression frequency of TSGs (rows) in all cell tracks (columns, ordered by cell type). Black boxes indicate the focus tissue of each TSG. **H** Comparison of the density of miRNA binding sites in the 3′UTRs of TSGs exhibiting different levels of leaky transcription. Leaky transcription was classified either by the observed expression frequency in cell tracks differentiating into alternative tissues (left) or by the expression levels in alternative tissue types relative to that in the focus tissue (right). The density was calculated based on all reliable miRNAs, and multiple binding sites of the same miRNA were calculated only once. Data presentation is identical to (**F**). Statistics: Mann–Whitney *U* test, two-tailed. **I** Scatter plot of the density of miRNA binding sites of TSGs versus the frequency of leaky transcription. **J** Scatter plot showing the frequency of leaky transcription and cross-tissue preferential targeting by miRNAs for different types of TSGs. **K** Comparison of protein levels of three neuronal-specific TFs in non-neuronal cells relative to neuronal cells, before and after depleting miRNA biogenesis. Data presentation is identical to (**F**). The two percentages listed above indicate the increased level and the fraction of cells with an increase after perturbation (red lines). Statistics: paired *t* test, two-tailed. Micrographs show expression changes in representative cells. **L** Changes in protein expression levels of ELG-44, EGL-46, and TTX-1 in representative cells. **M** Model of the role of miRNA regulation in increasing differentiation fidelity.

Subsequently, we examined the properties of miRNA targets. To elucidate cell differentiation, we identified genes with enriched transcription in each tissue type, referred to as tissue-specific genes (TSGs). We then analyzed the miRNA targeting preferences towards different types of TSGs (TSGs associated with different tissues, Fig. 8A). Specifically, within each miRNA-pr-expressing cell track, we calculated the preferential targeting of miRNAs against different TSGs by determining the ratio of observed to expected (O/E) targeting frequency (Methods). As depicted in Supplementary Fig. 15A, each miRNA displayed distinct O/E targeting frequencies against different types of TSGs, with varying magnitudes across cell tracks leading to different terminal cells (Supplementary Data 15). Hence, different miRNAs exhibit varying targeting preferences against distinct cell differentiation programs (represented by TSGs) in a context-specific manner.

We integrated the preferential targeting profile with the tissue specificity of miRNAs and globally determined which tissue-specific miRNAs preferentially target which types of TSGs (Fig. 8B and Supplementary Fig. 15B). We identified three general patterns. First, same-tissue preferential targeting, where a tissue-specific miRNA preferentially targets TSGs in the same tissue, was significantly less common; the observed frequency was only 36% of the expected (Fig. 8C). Second, although cross-tissue preferential targeting was prevalent, miRNAs sharing the same tissue specificity tended to target different types of TSGs. For example, even though miR-235 and miR-79 are both specific to the hypodermis, miR-235 primarily targets pharynx-TSGs, while miR-79 targets TSGs related to both pharynx and neurons (Fig. 8B). Third, TSGs specific to certain tissues were globally enriched or depleted as preferential targets of miRNAs (Fig. 8D). Notably, TSGs associated with neurons and glia exhibited significantly higher frequencies of preferential targeting, whereas those related to the hypodermis and P cells showed much lower frequencies. Many neuron-TSGs, including known fate determinants, contain one or more binding sites predicted to be targeted by non-neuronal miRNAs (Supplementary Fig. 15C). These findings predict that tissue-specific miRNAs repress regulatory programs associated with different tissue types.

As proposed in previous studies^11,97,99,101–103^, one possible role of cross-tissue preferential targeting of miRNAs in a tissue is to repress the translation of leaky transcripts associated with alternative fates. Although transcripts of TSGs were significantly more abundant in the focus tissue, they were also present across many other tissues, termed “leaky” transcription (Methods and Fig. 8E). This phenomenon was highly pervasive and variable among different types of TSGs. On average, a TSG was detected in 33% of cell tracks that differentiate into other tissue types, with the highest level detected in neuron-TSGs and low levels in P cell- and intestine-TSGs (Fig. 8F and Supplementary Fig. 16A). Across the cell lineage, 43% of TSGs exhibited leaky transcription in cell tracks differentiating into a different tissue (Fig. 8G). For example, 67% of neuron-TSGs were also expressed in non-neuronal cell tracks. We verified that leaky transcription was prevalent even when using highly stringent definitions of TSGs (Supplementary Fig. 16B, C). This pervasive leaky transcription can potentially compromise differentiation fidelity by co-expressing genes promoting distinct fates. While we cannot rule out the alternative explanation that leaky transcription might suggest that a TSG associated with one tissue exerts functions in other tissues, we observed that the protein production of leaky transcripts was significantly lower than mRNAs present in the focus tissue (Supplementary Fig. 16D, E). This observation implies that many leaky transcripts do not result in the production of corresponding proteins.

Notably, we found that miRNA expression and preferential targeting were associated with repressing leaky transcripts. First, the 3′UTRs of TSGs with high levels of leaky transcription exhibited a significantly higher density of miRNA binding sites (Fig. 8H), and the level of leaky transcription positively correlated with binding site density (Fig. 8I). More explicitly, the leaky transcription of a TSG was positively correlated with its tendency to be preferentially targeted by miRNAs (Fig. 8J). For example, neuron-TSGs exhibited the highest frequency of leaky transcription and cross-tissue preferential targeting by tissue-specific miRNAs.

Finally, we focused on neuron-TSGs, which exhibited the most prevalent leaky transcription and miRNA preferential targeting, and examined the role of miRNAs in repressing protein production of leaky transcripts. We globally perturbed the activity of miRNA biogenesis by depleting the Microprocessor complex (Drosha and Pasha/DGCR8) using a previously described method (Supplementary Fig. 17A)^3^. We compared the protein expression levels of four neuronal-specific TFs exhibiting leaky transcription (ZTF-11/MYT1L, SOX-2/SOX2, CND-1/NEUROD1, and HLH-3/ASCL1) and observed an increase in the protein expression levels of three TFs in certain non-neuronal cells relative to neuronal cells (Fig. 8K and Supplementary Data 16). HLH-3 showed a significant global reduction in protein expression upon miRNA perturbation and was consequently excluded from the analysis (Supplementary Fig. 17B). It suggests that miRNA-mediated regulation represses the ability of certain neuronal leaky transcripts to produce proteins. Additionally, we perturbed a pharynx and muscle-specific miRNA, miR-1, and found that the protein expression levels of three neuronal-specific TFs (EGL-44/TEAD1, EGL-46/INSM2, and TTX-1/OTX1) predicted to be miR-1 targets were significantly increased in specific non-neuronal cells (mostly pharyngeal cells) (Fig. 8L, Supplementary Fig. 17C–E and Supplementary Data 17). 3′UTR reporter assay confirmed that ELG-46 and TTX-1 are likely miR-1 targets. Upon removal of miR-1 binding sites from the 3′UTR, protein levels of corresponding reporters were significantly increased (Supplementary Fig. 17F and Supplementary Data 18). These findings suggest that an individual miRNA represses protein production of leaky transcripts of neuron-TSGs in non-neuronal cells.

In summary, our analysis of transcriptional regulation and the leaky expression of predicted miRNA targets reveals a regulatory framework for miRNAs in promoting differentiation fidelity (Fig. 8M). Tissue fate determinants activate both miRNAs and sets of tissue-specific genes to initiate cell differentiation programs. Subsequently, miRNAs function coherently with fate determinants, guiding differentiation by avoiding targeting tissue-specific genes responsible for the original fate. Instead, they preferentially target leaky transcription of genes associated with alternative fates, thereby repressing protein expression and enhancing differentiation fidelity.

## Discussion

We present scCAMERA, a lineage-resolved cartography of miRNA promoter activity with single-cell annotation clarity that encompasses *C. elegans* embryogenesis. It fills a gap by providing a whole-body, cellular-resolution developmental expression atlas of miRNAs in a metazoan species. Our approach has unavoidable drawbacks, particularly the following caveats when interpreting specific expression patterns. First, the promoter sequence included in the reporter may not fully recapitulate endogenous gene expression, though many studies have validated that a 2 kb promoter satisfactorily recapitulates miRNA expression and function^104–109^. In addition, the 3′ sequence of endogenous miRNA locus, which might regulate its transcription, was not included in the reporter. Second, transgenes are prone to germline silencing^110,111^, which causes false negatives in detecting some maternally-expressed miRNAs, such as miR-51^112^. Third, the limited detection sensitivity of mild imaging parameters applied for long-term imaging may neglect weak fluorescence signals. Fourth, certain miRNAs may be subject to specific turnover mechanisms after transcription^113^, and the reporter approach will miss such processes. Finally and less frequently, some miRNAs expressed in one cell are secreted and transported into another^114,115^; our approach cannot detect such nonautonomously-expressed miRNAs. Although miRNA secretion has been observed in aging^116,117^, its prevalence in normal embryogenesis remains to be formally assessed. Despite the caveats, as systematically validated by a series of quality assessments and case studies, the scCAMERA generally demonstrates good expression accuracy and high annotation clarity.

We reveal general properties of miRNA expression that are not immediately evident from tissue-level analyses. Many miRNAs exhibited sub-tissue and cell-specific expression, which partially explains the general lack of organismal phenotypes upon individual miRNA perturbation^8,9^ and underscores the value of scCAMERA in illuminating cellular and developmental contexts to miRNAs. Furthermore, we find that the number of tissue-specific miRNAs varies considerably across tissues. For example, pan-muscle-specific miRNAs are scarce, whereas sub-digestive-tissue-specific miRNAs are abundant (Fig. 3K). Besides food intake and metabolism, *C. elegans* digestive tissues participate in diverse processes, including the starvation stress response, cross-tissue signaling, and aging^118–122^. Whether this digestive tissue enrichment is conserved across species and whether miRNA-dependent pathways broadly define specific properties and functions of the digestive system await further investigation.

A broadly applicable feature of the scCAMERA is to allow expression-informed functional analysis, which is particularly valuable given that the loss of individual miRNAs or even an entire family usually does not elicit prominent phenotypes^8,9,12^. As demonstrated in the case studies, the cellular expression-informed strategy effectively revealed unrecognized roles of miRNAs in the pharynx and excretory canal development (Figs. 4–6). Interestingly, although predicted by expression, both phenotypes are relatively mild or restricted to a specific cell, which could easily be overlooked if the phenotypic analysis is not guided by high-resolution expression. Although miRNAs have been widely implicated in stress or pathological conditions^123,124^, the functions of individual miRNAs in normal development remain largely unknown^12^. We envisage that scCAMERA and the expression-informed strategy could facilitate the dissection of miRNA-dependent developmental regulation by (i) providing the single-cell developmental contexts of miRNA functions, (ii) guiding the design of context-specific phenotypic assays, (iii) narrowing down candidate genetic interactors of miRNAs (e.g., another miRNA or other regulatory genes), and (iv) enabling systems-level integrative analysis of miRNA functions during in vivo development.

The fact that miRNAs function to suppress leaky transcripts and thus reduce the noise of cell differentiation programs has been documented in multiple organisms^97,99,101,102^ and proposed as a general scheme for increasing developmental robustness^11,103^. In these reports, miRNAs repress leaky gene transcription from neighboring tissues derived from the same progenitor cells. Our single-cell analyses corroborate and extend the above model in several ways. First, we explicitly define leaky transcription of genes associated with one differentiation program in other tissues and predict that miRNAs preferentially target leaky transcripts. This finding suggests that the combinatorial interaction of TFs alone may be insufficient to generate exclusive tissue- or cell-type transcriptional programs, although we cannot exclude the possibility that some seemingly leaky transcripts have a relevant biological function. Our data suggest that miRNAs may function post-transcriptionally by targeting leaky transcription to increase specificity. Second, we found that at least in *C. elegans*, leaky transcription, and miRNA preferential targeting are not necessarily restricted to tissues with common lineage origin. It suggests that leaky transcription may be caused by complex relationships of transcriptional regulators rather than strictly through cell lineage, though the latter offers a simple explanation of how leaky transcription could occur. Third, we find that different tissue-specific miRNAs exhibit preferential targeting against neuronal-specific genes, which exhibit widespread leaky transcription (Fig. 8J), suggesting that one function of miRNAs might be to repress neuronal fate potential. This is consistent with the hypothesis that neuronal fate might be the default during development^125–128^. While non-neuronal miRNAs often target genes associated with neuronal differentiation, the impact of individual miRNAs appears to be modest. This is indicated by only a slight increase in protein expression from seemingly leaky transcripts following individual miRNA depletions. It suggests that either a synergistic effect of multiple miRNAs or the involvement of other factors might be at play. Finally, we show that tissue-specific miRNAs are activated by fate determinants (Fig. 7), and, as described previously, these miRNAs tend not to target TSGs of the same tissue (Fig. 8C)^97–99^. This general principle reveals a coherent function of fate-determining TFs to co-activate regulatory programs that promote specific cell fates and miRNAs that inhibit alternative fates.

## Methods

### ***C. elegans*culture**

Unless otherwise indicated, all *C. elegans* strains were maintained at 21 °C on nematode growth media (NGM) plates seeded with OP50 bacteria. The complete list of *C. elegans* strains used in this study and their genotypes is provided in Supplementary Data 1.

### Selection of miRNAs

We selected 54 miRNAs based on conservation, expression status, and suitability for constructing transcriptional reporters. We focused on the 73 miRNAs encoded in the *C. elegans* genome that are conserved in *Drosophila* and humans^42^, and then integrated the expression status^18,43–45^, which revealed 61 conserved miRNAs that are expressed during embryogenesis or at the L1 stage. Finally, we excluded seven miRNAs that are located in introns and transcribed in the same direction as the surrounding protein-coding gene (host gene, Supplementary Fig. 1A), as their transcription is likely controlled by the host gene^129^.

### Generation of reporter strains to indicate transcriptional activity of miRNAs

The minimal Mos1 transposon (miniMOS) was used to generate transgenic *C. elegans* strains, each with a single copy of mNG::H2B driven by a miRNA promoter integrated into a random genomic position^46^. First, a backbone plasmid (pXWN322, mNG::H2B::*tbb-2* 3′UTR) for generating miRNA reporters was constructed by replacing the *eft-3p*::GFP region (position 340–1843 bp) of the pCFJ914 plasmid (*eft-3p*::GFP::H2B::*tbb-2* 3′ UTR, Addgene plasmid #44489) with a multiple cloning site (MCS, AscI-AflII-ApaI-MluI-SacII) followed by the mNG::H2B coding sequence^130^. Second, the promoter sequences of all selected miRNAs were synthesized or PCR-amplified and cloned into the MCS to generate transcriptional reporters for each miRNA (*miRNAp*::mNG::H2B::*tbb-2* 3′UTR). The promoter sequences and restriction sites used are listed in Supplementary Data 2. Third, microinjection and selection of miniMOS transgenesis were performed following a previous protocol^46^. The JIM113 strain carrying a ubiquitously expressed histone::mCherry transgene for cell lineage tracing was used as the background for microinjection. A mixture consisting of 10 ng/µL of miRNA reporter plasmid, 50 ng/ µL of pCFJ601 (*eft-3p*: Mos1 transposase, Addgene plasmid #34874), 10 ng/µL of pCFJ104 (*myo-3p*::mCherry, Addgene plasmid #19328), 10 ng/µL of pGH8 (*rab-3p*::mCherry, Addgene plasmid #19359), 2.5 ng/µL of pCFJ90 (*myo-2p*::mCherry, Addgene plasmid #19327) and 10 ng/µL of pMA122 (*hsp-16.41p*::peel-1, Addgene plasmid #34873) was injected into worm gonads, after which the injected animals were incubated at 25 °C on NGM plates seeded with HB101 bacteria. The day after injection, 200 µL of G418 at 25 mg/mL was added to the NGM plates (35 mm diameter), and the worms were allowed to grow until starvation. Animals without the fluorescent co-injection markers that survived heat shock (34 °C for 2 h) were picked to a new plate, propagated, and genotyped to obtain homozygous transgenic strains. To overcome position effects on transgene expression^110,131^, at least two independent integration strains were obtained for each miRNA transcriptional reporter.

### Embryo mounting and 3D time-lapse imaging of embryogenesis

Collection and mounting of early embryos were performed as done previously, with minor modifications^132^. Six to ten young-adult-stage worms with one row of embryos in the gonads were transferred by a worm picker onto a Multitest slide (MP Biomedicals) with a droplet of egg buffer. Then, the worms were cut open under a dissecting microscope (Nikon, SMZ745) to release the embryos, and embryos at the two- to four-cell stage were transferred using an aspirator tube assembly (Sigma–Aldrich) into a small droplet (~2 µL) of egg buffer containing 20 μm polystyrene microspheres (PolyScience) on a coverslip (Fisherbrand). Next, the positions of embryos on the slide were adjusted with an eyelash to form three clusters, each with two to three embryos. Finally, the slide was covered with an 18 × 18 mm coverslip, which was sealed with Vaseline.

3D time-lapse imaging of *C. elegans* embryogenesis was performed using a spinning-disk confocal microscope (Revolution XD). The system is equipped with an Olympus IX73 inverted microscope body, a Yokogawa CSU-X1spinning-disk unit, an ASI PZ-2150 XYZ stage with Piezo-Z positioning, an integrated solid-state laser engine (Coherent, 50 mw at 488 nm and 50 mw at 561 nm), and an Andor iXon Ultra 897 Electron Multiplying Charge-Coupled Device. Images were acquired under a PLAPON 60XO objective (N.A. = 1.42) using the multidimensional acquisition module of MetaMorph software (Molecular Devices) to scan three slide positions, and for each position, 30 Z focal planes were scanned with 1 µm spacing. Images were taken over 350–400 consecutive time points for most strains at a time interval of 75 s to capture embryogenesis up to the comma or 1.5-fold stage. Image parameters were similar to those used in previous studies^31,133^ for which laser power and exposure time were optimized to obtain images with a high signal-to-noise ratio while minimizing photodamage to embryos. To compensate for the decay of fluorescence signal across Z focal panes, laser power was increased by 3% for every Z plane for both mCherry and mNG when the focal plane went deeper into the embryo. After long-term imaging under the above settings, all wild-type embryos hatched without apparent morphological abnormalities. Unless otherwise noted, all live imaging was performed at 20 °C ambient temperature.

### Cell lineage tracing and reporter expression quantification

The StarryNite software was used to recognize and trace all cells and reconstruct the cell lineage. Briefly, a hybrid 2D/3D algorithm was deployed to segment all nuclei from the 3D image stacks using the nucleus-localized, ubiquitously-expressed histone::mCherry signals^134^. Next, a semi-local neighborhood-based framework was applied to match the nuclei detected in a previous time point to those in the next time point; one nucleus was linked to two successive nuclei (daughter cells) if a cell division was detected^135^. To construct the cell lineage, the automatically generated cell identification and tracing results were manually inspected and curated using AceTree, a cell lineage navigation, editing, and visualization software^136,137^. After lineage tracing, each cell was assigned a unique name following Sulston’s nomenclature. The initial identity of each cell was determined at the two- or four-cell stage according to stereotypic localization and division timing differences. Then, the full name of each mother cell was propagated to its daughters, and a new letter was added to specify the position of the cell relative to the body axis after division. There are three types of cell divisions: anterior-posterior (a/p), left-right (l/r), and dorsal-ventral (d/v); accordingly, the letters appended to daughter cell names were a/p, l/r, and d/v. For example, ABp is the daughter of AB that localizes more posteriorly, while ABpr is the daughter of ABp positioned on the right side. The naming of most cells follows the above rules, with a few exceptions (EMS, MS, E, C, D, P_1_, P_2_, P_3_, P_4_, Z2, and Z3) whose names recognize their developmental characteristics. Details of the nomenclature rule^47,51,134^ and cell lineage error detection, correction, and quality control^31,138^ are described elsewhere. For each reporter strain, cell lineages of at least two embryos were traced until the 350-cell stage, and one was selected to be traced until the bean-to-comma stage, at which ~90% of all embryonic cells have been generated.

Quantifying miRNA reporter expression in each cell was performed as done previously^31^. Each miRNA reporter strain expresses two fluorescent proteins: the ubiquitously expressed histone::mCherry, used for cell lineage construction as described above, and the nucleus-localized mNG::H2B driven by a miRNA promoter, used for miRNA expression quantification. Identifying and tracing nuclei enabled a straightforward quantification of mNG intensity in each nucleus at each time point. For this quantification, mNG expression was taken as the average fluorescence intensity of all pixels within the nucleus after subtracting the local background fluorescence intensity. Fluorescence intensity in the center z plane of the nucleus was used to approximate the intensity in the entire nucleus. Local background fluorescence intensity was estimated using a previously established method, which quantifies the average fluorescence intensity within an annular area between radii of 1.2*x* and 2*x* the nuclear radius from the centroid of each target nucleus, excluding nearby nuclei that overlap the area^30^. To describe miRNA reporter expression in a cell, the background-subtracted mNG intensity was averaged across all traced time points of that cell. Because fluorescence intensity attenuates when the Z plane goes deeper in sample^139^, which might affect comparisons between embryos with different orientations, a previously described decay-correction approach was applied^31^. To identify the actual expression signals, a reporter strain carrying the mCherry but not the mNG transgene was imaged and processed with the identical method to approximate the background fluorescence intensity. An expression cutoff of 3.2 was applied to call expression, under which the false discovery rate of mNG expression was 0.01% across all cells at all time points; values below this cutoff were set to zero.

Due to the cumulative nature of mNG::H2B intensity over time, we investigated whether fluorescence intensity increased at all traced time points for each cell to predict the activity of a miRNA promoter in the cell. We made the assumption that the increased intensity resulted from newly translated fluorescent protein in the cell. To accomplish this, we divided the intensity values into early and late time windows and identified cases where the intensity in the late time window was significantly higher (Mann–Whitney *U* test, *Q* corrected by the Benjamini-Hochberg procedure <0.05) than in the early time windows, indicating an increase in expression. To ensure a reliable comparison, the analysis was limited to cells with 12 or more traced time points. If more than 20 time points were traced, the first 10 time points were classified as the early time window; otherwise, the first half was considered the early time window. Lastly, the onset cell in a cell track showing expression was automatically classified as having increased expression. It should be noted that the prediction of transcriptional activity of a miRNA-pr in a cell should be treated with caution as a highly stable mRNA of the reporter inherited from an earlier cell could also cause an increased fluorescence intensity. Interpreting the results of inferred transcriptional activity of a miRNA-pr in a cell requires careful consideration. The potential presence of a highly stable mRNA from the reporter, inherited from a preceding cell, may lead to elevated fluorescence intensity. This factor could obscure the actual transcriptional activity, thereby complicating the interpretation of results.

### Identification of reporter strains with the most representative expression patterns

For multiple transgenic strains bearing reporters for the same miRNA, we analyzed their embryonic expression patterns to select the strain with the most representative expression. The following rules were applied under the principle of selecting a highly representative, informative pattern (Supplementary Fig. 1E). First, if all reporter strains exhibited expression (identical or distinct patterns), we selected the one with the highest expression and predominant pattern (if multiple patterns were observed) (type 1; 22 miRNAs, see Supplementary Fig. 1F, G for two representative examples). Second, if both expression and non-expression were detected and the non-expression strain was the predominant pattern, we performed a literature search to check whether this miRNA was previously reported to be expressed during embryogenesis (by reporter assay or sequencing) and determine which pattern was more representative (type 2; 8 miRNAs). This treatment is meant to maximize the expression information provided in this study. Third, if all strains exhibited non-expression during the lineage tracing time window (until the bean-to-comma stage), we further visually examined the images of later-stage embryos (from the comma to the 3-fold stage) and selected the strain exhibiting the highest later expression as the representative pattern (type 3; 10 miRNAs). Finally, if all strains exhibited non-expression in the time window of lineage tracing and at later stages, we randomly selected one as the representative pattern (type 4; 8 miRNAs were in this category). If expression patterns in more than one strain were identified as representative, all of them were kept (only miR-236 fell in this category). The strains exhibiting representative expression were then used to analyze cellular expression throughout this study.

### Quantification of miRNA expression levels in lineage-resolved cells at the L1 stage

Worm collection, fixation, and DAPI-staining were performed using a protocol modified from a previous study^32^. Briefly, larvae hatched within three hours were washed by M9 buffer, spun down, and then re-suspended in a 4% paraformaldehyde solution prepared in modified Ruvkun’s witches brew (MRWB) and frozen in liquid nitrogen. Worms were kept in liquid nitrogen for 24 h, thawed at 4 °C, and then kept in rotation for more than eight hours, after which they were washed using Tris–Triton Buffer with 100 mM DTT for 5 min and then stained by DAPI or Hoechst at 1 μg/mL for 3 h. After staining, worms were washed with TTB five times and mounted in 60% glycerin for microscopy.

A Zeiss confocal microscope with a 63× oil lens was used to scan stained L1 larvae with X, Y, and Z dimension sampling respectively set at 0.116 µm and 0.122 µm per pixel. Then, the image analysis pipeline CellExplorer was used to computationally straighten these 3D image stacks^49^. DAPI-stained nuclei in each image stack were segmented automatically^49^ and then manually curated using the VANO interactive interface. Manual annotation of nuclear identities was based on the prototypical morphology and relative spatial positions of nuclei in *C. elegans* previously described in the WormAtlas website (http://www.wormatlas.org) and literature^140,141^.

### Comparison of miRNA reporter expression to miRNA-seq results

To facilitate comparison with global miRNA-seq data, we classified miRNAs into three categories based on the number of miRNA-pr expression cells (*N*_exp_) observed in this study: high (*N*_exp_ > 200 cells), medium (10 < *N*_exp_ ≤ 200), and low (*N*_exp_ ≤ 10 cells). Subsequently, miRNA-seq expression levels (counts per million, CPM) were compared between miRNAs within each category.

Similarly, to compare with tissue-level miRNA-seq data, we categorized miRNAs into three categories based on their expression frequency (*F*_exp_) in cells from corresponding tissues detected in this study: high (*F*_exp_ > 0.7), medium (0.3 < *F*_exp_ ≤ 0.7), and low (*F*_exp_ ≤ 0.3). Following this classification, miRNA-seq expression levels in corresponding tissues were compared between miRNAs within each category.

### CRISPR/Cas9-mediated gene editing

Gene deletion: The pDD162 plasmid (Addgene plasmid #47549), which contains the expression cassettes for single-guide RNA (sgRNA) and Cas9 protein, was used as the backbone to construct gene-editing plasmids. Two sgRNAs that target selected regions of the gene of interest were designed by the CRISPR Design Tool (http://zlab.bio/guide-design-resources), and the DNA sequences were synthesized and cloned into the pDD162 plasmid (Addgene plasmid #47549) under the control of the U6 promoter. The plasmid pRF4, which induces a dominant roller phenotype, was used as the transgenesis reporter. The above plasmids were purified using the PureLink PCR Micro kit (Invitrogen, K310050), mixed at a final concentration of 50 ng/µL for each, and injected into the gonads of worms. F1 worms exhibiting a roller phenotype were transferred to new NGM plates with one worm on each plate and allowed to produce a sufficient number of F2 worms. Then, the F1s were lysed and used for detecting gene-editing events by PCR with primers flanking the expected deletion sites. Finally, homozygous F2s generated by F1 worms with the expected gene editing were identified by PCR and sequencing.

DNA sequence replacement: Except for utilizing an additional homologous recombination (HR) repair template plasmid, the procedure for performing CRISPR/Cas9-mediated DNA replacement was identical to the gene deletion experiments. The DNA sequence to replace the endogenous regions and the 5′ and 3′ sequences (~800–1000 bp each) homologous to those flanking the endogenous region were synthesized and cloned into the pPD95_77 vector (Addgene plasmid #1495). Point mutations were introduced into the HR template sequences recognized by the sgRNAs to preclude cutting of the sequence by Cas9; these mutations were also designed to introduce a new restriction endonuclease recognition site to facilitate genotyping.

The sequences of sgRNAs, the HR template, and primers used for genotyping are provided in Supplementary Data 1.

### Clustering of cells based on miRNA expression

The cellular expression of all miRNA reporters during embryogenesis and at the L1 stage was combined to perform cell clustering. First, to compare the embryonic and L1 expression of the same reporter, absolute expression levels during embryogenesis and at the L1 stages were normalized to the reporter’s respective percentile rank. For the embryonic dataset, cellular expression was averaged along the cell tracks (a sequence of temporally ordered mother-daughter cells) that generate the 558 embryonic terminal cells to represent miRNA expression during the development of each terminal cell. Cells at the 4-cell stage were used as the origins of cell tracks, and only miRNAs that were continuously expressed in at least two consecutive cell generations within a cell track were considered to be expressed and hence averaged. Then, the maximum percentile rank expression among cell tracks during embryogenesis and cellular expression in the equivalent terminal cell at the L1 stage was determined, and the embryonic and L1 expression of each miRNA was integrated for each terminal cell. Second, unsupervised hierarchical clustering was employed to group cells, utilizing Kendall’s Tau as the distance metric and average linkage clustering as the agglomeration method. Finally, the confidence level for each cluster was evaluated via bootstrap clustering with 1000 iterations, utilizing the R package “pvclust”^142^. For determining cell clusters, a confidence score of AU (Approximately Unbiased) ≥90, as previously applied^143^, was adopted.

### Identification of tissue-specific miRNAs, TFs, and protein-coding genes

Identification of tissue-specific miRNAs at embryonic and L1 stages: All terminal cells were classified into the following 11 tissues or cell types: neuron (202 cells, pharyngeal neurons not included), glia (40 cells), hypodermis (48 cells), seam cell (20 cells), P blast cell (P cell, 12 cells), pharynx (95 cells), intestine (20 cells), rectum and anus (15 cells), body wall muscle (87 cells, including the GLR cells), coelomocyte (4 cells), and excretory system (6 cells). Both quantitative and binarized expression were used to identify the preferential expression of miRNAs in tissue or cell types. A miRNA was identified as exhibiting tissue-specific expression if it met one of the following criteria. First, quantitative miRNA expression in all cells of a tissue type was significantly higher than that in the rest of the cells (fold change >2 and *Q* < 0.01, two-tailed Mann–Whitney *U* test). Second, the fraction of all cells in a tissue type with miRNA expression (binarized) was significantly higher than expected (fold change >1.25, *Q* < 0.01, two-tailed Fisher’s exact test). Third, all cells (≥3) expressing the miRNA belonged exclusively to a tissue type, even if the *Q*-value did not reach statistical significance. All tissue-specific miRNAs were further classified as exhibiting pan- or sub-tissue preference based on whether the fraction of cells with miRNA expression in a tissue type exceeded 70%.

Identification of tissue-specific TFs at the L1 stage: The above method was also applied to identify tissue-preferential TFs using a single-cell expression atlas of TFs at the L1 stage based on promoter-fusion fluorescent reporter and image analysis^50^. If multiple promoter-driven reporter strains and, hence, expression patterns existed for the same TF, tissue specificities detected in all strains were combined. During the process, a TF was classified as pan-tissue-specific if it exhibited both pan-tissue and sub-tissue enrichment patterns, as evidenced by different reporters for the same TF.

Identification of TSGs during late embryogenesis: Transcript per million (TPM) values of a recently published lineage-resolved single-cell transcriptome of *C. elegans* embryogenesis were used to identify embryonic TSGs^25^. For all cell tracks leading to the 558 terminal cells, the maximum expression between a terminal cell and its mother cell was calculated and taken to represent the expression of the given gene in late embryos. If a terminal cell was not covered by the study, the latest cell was used as a proxy. In many cases, a transcriptome was assigned a cell lineage identity corresponding to more than one cell; these cells were therefore considered as having identical transcriptomes. Then, the identical method used to identify tissue-specific miRNAs was used to identify TSGs.

### Progenitor fate transformation

To determine whether the tissue-specific expression of miRNAs is coupled to the developmental fates of progenitor cells, we transformed the fates of progenitor cells and determined whether the cellular expression of relevant miRNAs changed accordingly (Supplementary Fig. S7). Two sets of fate transformations were performed. First, we changed the fate of the ABalp progenitor cell to that of the wild-type ABarp progenitor cell by knocking down *lag-1/CLS*, a Notch signaling effector (Supplementary Fig. 7A)^48,144^. To evaluate the coupling of miRNA-pr expression pattern to developmental fate, we analyzed two miRNA reporters (miR-1-pr and miR-79-pr) that exhibited differential expression between ABalp- and ABarp-derived cells in wild-type embryos. The miR-1 reporter is normally expressed in ABalp- but not ABarp-derived cells, whereas miR-79-pr is mainly expressed in ABarp- but not ABalp-derived cells. Concomitantly, we found in *lag-1(RNAi)* embryos that the expression of both reporters in ABalp-derived cells resembled that of wild-type ABarp-derived cells (Supplementary Fig. 7B), consistent with the expectation that tissue-specific miRNA expression is coupled to the developmental fate of progenitor cells.

Second, we also assessed the expression-fate coupling by transforming the fate of the MS progenitor cell to that of the wild-type E cell by performing RNAi against *pop-1/TCF*, a Wnt signaling effector (Supplementary Fig. 7C)^48,145^. In this transformation experiment, we analyzed the expression of miR-2 and miR-75 reporters, of which the former is expressed in MS- but not E-derived cells and the latter in E- but not MS-derived cells. Again, expected fate-dependent expression changes were concomitantly observed, in which expression of both reporters in MS-derived cells of *pop-1(RNAi)* embryos resembled that in wild-type E-derived cells (Supplementary Fig. 7D).

### Comparison of miRNA expression to the literature

For each of the 54 miRNAs, we systematically checked whether its expression was previously analyzed and to what extent our expression pattern was consistent with previous reports^13,15,18,37,40,43,61–69^. Because most previous analyses of miRNA expression specificity did not compare expression levels across all cells, we standardized previous expression patterns into three categories reflecting the expression status: (1) expression is seen or enriched in a tissue type that was also defined in this study when identifying tissue-specific miRNAs (tissue-level expression), (2) expression is seen in specific cells or a subset of cells of a tissue type (cell-level expression), and (3) ubiquitously expression was observed. Only embryonic and L1 expression information was considered and combined. If a miRNA was described as expressed in a minority of the cells constituting a tissue (e.g., head neurons, amphid neurons, a subset of pharyngeal muscles, and posterior intestine), it was classified into a cell-level category. All expression descriptions that could not be classified into any of the above categories or for which no developmental stage information was provided were discarded to ensure a fair and accurate comparison.

Second, we classified the expression patterns obtained in this study into three categories reflecting enrichment and expression status: (1) enrichment in specific tissue types with embryonic and L1 results combined and pan- and sub-tissue enrichment distinguished, (2) expression coverage in different tissue types (cellular expression at the L1 stage was used for better representation of cell type), and (3) ubiquitous expression if the miRNA was expressed in 90% of embryonic cell tracks or L1-stage cells.

Third, each literature-described expression pattern was compared to the corresponding pattern obtained in this study, and the consistency between them was described categorically as “consistent”, “partially consistent”, or “not consistent” based on the following criteria. For a tissue-level description in the literature, the corresponding pattern was deemed “consistent” if pan-tissue enrichment was detected, “partially consistent” if sub-tissue enrichment was detected or if expression was detected in cells of the tissue, and “not consistent” if no enrichment or expression was observed. For a cell-level description in the literature, the corresponding pattern was deemed “consistent” if pan-tissue enrichment was observed, “partially consistent” if the expression was detected in at least one of the literature-documented cellular contexts, and “not consistent” if the cellular expression was not observed. For a ubiquitous expression description in the literature, the corresponding pattern was deemed “consistent” if the ubiquitous expression was also detected in this study.

Finally, the consistency categorizations of all expression descriptions from the literature were combined to determine overall consistency. If all descriptions were deemed “consistent” or “not consistent”, we accordingly concluded that our expression was consistent or not consistent with the literature. Otherwise, we deemed our results partially consistent. If a miRNA was previously analyzed in multiple studies, the best consistency categorization was used.

### RNAi

RNAi-mediated gene knockdowns were performed using a previously described feeding or injection procedure^146,147^. For feeding, L1-stage worms (10–15) were fed with bacteria expressing double-stranded RNAi against a target gene on RNAi plates containing 3 mM isopropyl-β-D-thiogalactoside. For injection, double-stranded RNAs were prepared using the HiScribe T7 Quick High Yield RNA Synthesis Kit and injected into worm gonads at a concentration of 1000 ng/μL. All RNAi clones were from the *C. elegans* RNAi library constructed by J. Ahringer’s group (Source BioScience) or the *C. elegans* ORFeome Library v1.1 (Horizon Discovery)^148^. The correctness of inserts of all clones used in this study was confirmed by sequencing.

### TF binding data

Genome-wide binding of TFs determined by ChIP-seq was obtained from the model organism Encyclopedia of Regulatory Networks (modERN) database^75,76^. Only ChIP-seq peaks of a TF located within −2000 bp to +100 bp relative to the transcription start site of a miRNA gene were considered. Only ChIP-seq experiments performed using embryonic or L1 samples were included, and those datasets with less than 100 peaks identified were excluded.

### Generation of miRNA reporters with TF-bound regions removed

To directly compare the influence of TF binding sites/regions on miRNA reporter expression, mNG::H2B reporters driven by miRNA promoters with and without the binding sites or peak regions of a TF were integrated into the same genomic region by the universal Mos1-mediated Single Copy Insertion (MosSCI) method^46,149^. The expression cassettes of corresponding miRNAs were cloned into the pCFJ350 plasmid (Addgene plasmid #34866) that enables the targeted insertion. Each miRNA reporter plasmid (50 ng/µL) was then mixed with plasmids pCFJ601 (50 ng/µL), pCFJ104 (10 ng/µL), pGH8 (10 ng/µL), pCFJ90 (2.5 ng/µL), and pMA122 (10 ng/µL) and injected into a strain that harbors a universal MosSCI insertion site oxTi365 on Chr. V, along with a lineaging marker. The injected animals were grown at 25 °C on NGM plates seeded with HB101 bacteria until starved. Plates were then incubated at 34 °C for 2 h, and putative insertion worms, as judged by moving like wild-type animals (with the uncoordinated phenotype rescued) and lacking the fluorescent co-injection markers, were picked to a new plate, allowed to propagate, and genotyped to obtain homozygous transgenic strains.

### RNA-seq and differential expression analysis

Embryos at the bean-to-comma stage were harvested using the alkaline hypochlorite bleaching procedure. Total RNA was extracted using the Trizol protocol, the purity and concentration were checked using NanoDrop 2000, and the integrity and quantity were measured using the Agilent 2100/4200 system. mRNA was purified from total RNA using polyT oligos and then fragmented into 300~350 bp fragments, which were then used as the template for reverse transcription to synthesize cDNA. Overhangs on the double-stranded cDNA were converted into blunt ends by exonuclease/polymerase activity. The 3′ ends of the DNA fragments were then adenylated, ligated with sequencing adaptors, and purified. Finally, the library fragments were amplified by PCR, and the products were purified to obtain the final library for Illumina sequencing. mRNA preparation, library preparation, sequencing, and quality control services were provided by Berry Genomics Co., Ltd.

All raw reads were processed to remove low-quality reads using fastp with default parameters (cut_window_size = 4 and cut_mean_quality =  20)^150^. Clean reads were mapped onto the *C. elegans* genome (WBcel235, https://genome-idx.s3.amazonaws.com/hisat/wbcel235.tar.gz) using the hisat2 software^151^, and duplicated reads caused by PCR amplification were further removed by sambamba markdup^152^. Read count per gene was determined by featureCount based on the genome annotation of WormBase (Caenorhabditis_elegans.WBcel235.75.gtf.gz)^153^, which was then converted to TPM values to quantify the expression of all protein-coding genes. Only those top-ranked genes that collectively constituted 99.9% of all mapped reads were included in the expression quantification to remove genes with extremely low expression. The TPM scores in each sample were then converted to rank percentiles to better represent expression changes in weakly expressed genes. Differential expression analysis was performed by DEseq2^154^, and a gene was identified as differentially expressed if it exhibited an expression rank percentile change greater than 10% and a *Q* value less than 0.01.

### Real-time quantitative reverse transcription PCR

The total RNA of embryos at the bean-to-comma stage was extracted using the standard TRIzol (Invitrogen, cat #15596026) protocol, and the mRNAs were reverse transcribed using the ReverTra Ace qPCR RT Master Mix containing gDNA Remover (TOYOBO, cat #FSQ-301). qRT-PCR was performed in triplicate on a CFX384 Real-Time System (BIO-RAD) using iTaq Universal SYBR Green Supermix (BIO-RAD). All primer sequences are provided in Supplementary Data 1. Relative expression levels of examined genes and internal controls (*tba-1* and *ubc-2*) were calculated by the 2^−ΔΔCt^ method, where Ct (cycle threshold) is the number of PCR cycles required for the fluorescent signal to exceed background^155^.

### Phenotype analysis

Quantification of cellular phenotypes: Cell cycle length, division asynchrony, and relative cell position of each traced cell were quantified and compared to the wild-type distribution using a previously established method^31^. A phenotypic change was assigned to a cell if its cellular behavior deviated significantly (q < 0.01) from the values observed in wild-type embryos.

Quantification of pharyngeal pumping rate: Pumping rate measurement was done as previously described^156^. Briefly, 24 h before experiments, L4-stage worms were transferred to and grown on an NGM plate seeded with OP50 bacteria. The feeding behavior of at least ten worms was filmed at 120× magnification for at least one minute using the MshOt MC25-M camera with a stereomicroscope (Leica M165FC). The pumping rate was manually determined under a 0.3× slow-motion play mode in a 30 s time window during which the worm was continuously feeding.

Quantification of canal cell length: Animals carrying a canal cell-specific transgene (*mir-232p*::mNG::PH) were used to visualize and quantify the length of the canal cell. Animals were anesthetized by levamisole (1 mg/mL), picked and arranged on a slide, and imaged using a Leica fluorescent stereo microscope (M165FC) with a MshOt MC25-M camera. As done previously^157^, the cell length was scored by eye on a scale ranging from 0 to 4 (with intervals of 0.5) based on where the canal cell extension ended relative to other body parts. A score of 4 denotes a full-length canal cell extending to the tail of the animal; 3 denotes the canal extending between the vulva (middle body) and tail; 2 denotes the canal cell stops approximately at the vulva; 1 denotes the canal cell extends between the cell body and the vulva; and 0 denotes the cell does not extend past the canal cell body.

### miRNA target prediction

Potential targets of each miRNA were obtained from the TargetScanWorm database (release 6.2), which lists the presence of sites matching a miRNA’s seed sequence in the 3′ UTRs of protein-coding genes^158,159^. Unless otherwise stated, only the 8-mer and 7-mer targets were used throughout this study (Supplementary Data 15).

### Analysis of preferential targeting of tissue-specific miRNAs

We analyzed the preferential targeting of each miRNA against each type of TSG (only pan-TSGs were considered to ensure a better representation of cell differentiation programs) in each cell track that differentiated into one of the 558 terminal cells. First, we determined the frequencies of TSGs within all genes for which 3′UTR sequences have been determined (*n* = 14,173 genes in the *C. elegans* genome). These frequencies served as the expectation of miRNA preferential targeting. Only genes with a 3′UTR ≥ 50 nt were included, and for genes with multiple 3′UTR sequences, the longest one was used. Second, for each miRNA expressed in each cell track (embryonic and L1 expression was combined), we determined the observed frequencies of TSGs among its predicted targets. Third, the ratio of the observed to expected (O/E) frequencies was calculated for each miRNA in each cell track. Since only transcribed genes could be targeted by a miRNA, the analysis was restricted to those genes transcribed in the cell track, and the O/E values varied across cell tracks. Finally, to account for intrinsic discrepancies in miRNA targeting frequency caused by differences in the length and sequence composition of 3′UTRs among different TSG types, the O/E value in each cell track was normalized by dividing the median O/E value across all cell tracks for each type of TSG.

The normalized O/E values were then used to determine the preferential targeting of each miRNA. We compared the normalized O/E values of all cell tracks that differentiate into a given tissue type to those of other tissues and identified preferential targeting if the median O/E value was >1, the fold change of normalized O/E values was >1.25, and the *P* value was <0.01 (two-tailed Mann–Whitney *U* test).

### Leaky transcription

Two different methods were used to quantify the leaky transcription of each TSG. The first method only considered the extent of leaky transcription, measured as the expression frequency of a TSG in all cell tracks that differentiate into tissue types other than the focus tissue (CT_other_). In the second method, expression in cell tracks differentiating into the focus tissue (CT_focus_) was considered, and the relative expression level of a TSG in CT_other_ relative to that in CT_focus_ was considered to represent the relative level of leaky transcription. In addition, to relate leaky transcription to miRNA preferential targeting, it was quantified at the cell track level as the fraction of TSGs exhibiting leaky transcription in the cell track. In this calculation, leaky transcription of each tissue type was measured and averaged. Only pan-TSGs were used to quantify leaky transcription.

### Statistics

All statistical analyses were performed using Python or GraphPad, including the Mann–Whitney U test, *t*-test, Hypergeometric test, Fisher’s exact test, and Wilcoxon Signed-rank test. Unless otherwise specified, all statistical tests were performed in two-tailed mode. The Benjamini–Hochberg correction procedure was applied to adjust the *P* values for multiple comparisons. No statistical method was used to predetermine sample size. The experiments were not randomized. No data were excluded from the analyses. More information on statistics is provided in Figure Legends and Methods.

### Reporting summary

Further information on research design is available in the Nature Portfolio Reporting Summary linked to this article.

### Supplementary information

Supplementary Information

Peer Review File

Description of Additional Supplementary Information

Supplementary Dataset 1

Supplementary Dataset 2

Supplementary Dataset 3

Supplementary Dataset 4

Supplementary Dataset 5

Supplementary Dataset 6

Supplementary Dataset 7

Supplementary Dataset 8

Supplementary Dataset 9

Supplementary Dataset 10

Supplementary Dataset 11

Supplementary Dataset 12

Supplementary Dataset 13

Supplementary Dataset 14

Supplementary Dataset 15

Supplementary Dataset 16

Supplementary Dataset 17

Supplementary Dataset 18

Reporting Summary

### Source data

Source Data

## Data Availability

The data supporting the findings of this study are available from the corresponding authors upon request. The 3D time-lapse image data and cell lineage tracing results used to construct the scCAMERA generated in this study have been deposited in OMIX (Open Archive for Miscellaneous Data), China National Center for Bioinformation, Chinese Academy of Sciences, under accession code OMIX002458. The raw RNA-seq data are available at the Genome Sequence Archive of the National Genomics Data Center of China, under accession code CRA009193. A web interface for navigating and visualizing scCAMERA is available at: https://dulab.genetics.ac.cn/scCAMERA. Source data for the figures and Supplementary Figs. are provided as a Source Data file.
