# Supplementary material for: A lineage-resolved cartography of microRNA promoter activity in C. elegans empowers multidimensional developmental analysis
Source: Nat Commun. 2024 Mar 30;15:2783. doi: 10.1038/s41467-024-47055-4 (PMC10981687; doi:10.1038/s41467-024-47055-4)
Supplement: Supplementary file 1 — Supplementary Information [file 41467_2024_47055_MOESM1_ESM.pdf]

Supplementary Information

A lineage-resolved cartography of microRNA promoter activity in *C. elegans* empowers multidimensional developmental analysis

Weina Xu[1,2,#], Jinyi Liu[1,2,#], Huan Qi[1#], Ruolin Si[3], Zhiguang Zhao[1,2], Zhiju Tao[3], Yuchuan Bai[3,] Shipeng Hu[3,] Xiaohan Sun[1,2], Yulin Cong[1,2], Haoye Zhang[1,2], Duchangjiang Fan[1,2], Long Xiao[1], Yangyang Wang[1], Yongbin Li[3]*, and Zhuo Du[1,2]*

[1] State Key Laboratory of Molecular Developmental Biology, Institute of Genetics and Developmental Biology, Chinese Academy of Sciences, Beijing, China.

[2] University of Chinese Academy of Sciences, Beijing, China.

[3] College of Life Sciences, Capital Normal University, Beijing, China.

[#] These authors contributed equally to this work

* Corresponding author

This PDF file includes:

Supplementary Figures 1-17

Supplementary References

Supplementary Figures

A

miRNA	Host gene/human ortholog
miR-58.1	*Y67D8A.2/PHKB*
miR-229	*gcn-1/GCN1*
miR-50	*tln-1/TLN2*
miR-62	*ugt-50/UGT2B7*
miR-64	*gcn-1/GCN1*
miR-65	*gcn-1/GCN1*
miR-66	*gcn-1/GCN1*

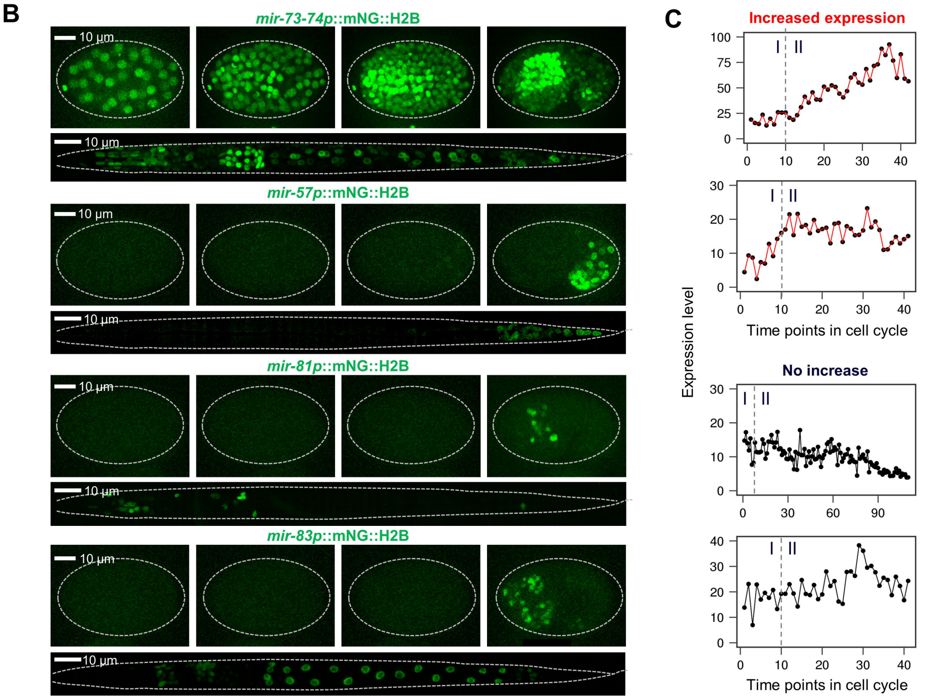

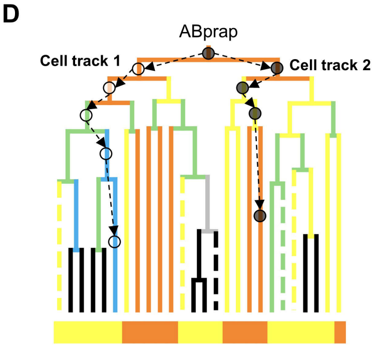

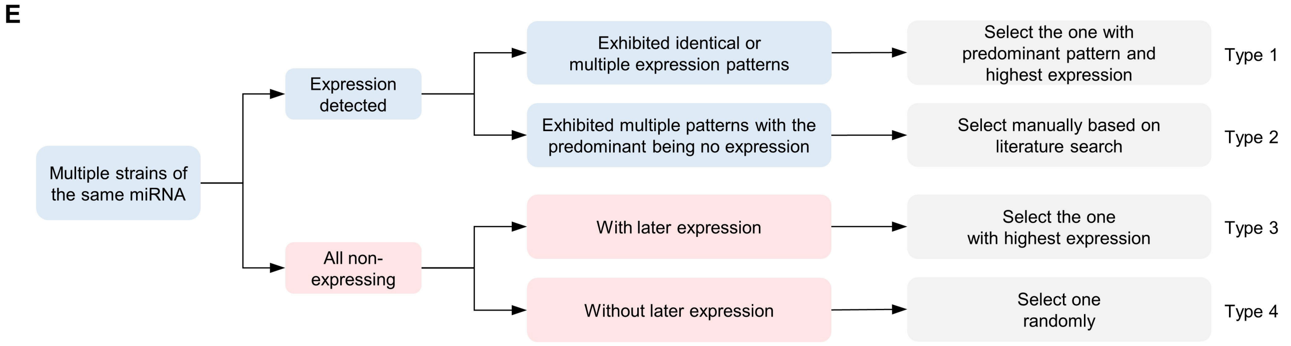

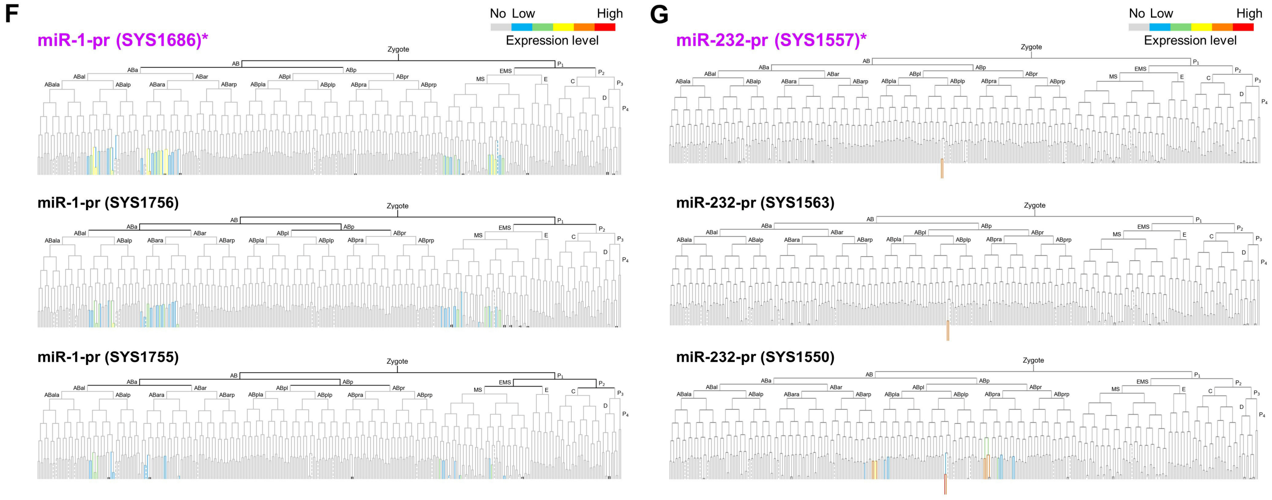

Supplementary Fig. 1. Construction of the scCAMERA.

(A) List of miRNAs whose promoter is located in the intron of another protein-coding gene and is transcribed in the same direction as the host gene. **(B)** Maximum projection images showing expression of representative fluorescence reporters of miRNAs at representative embryonic stages and the L1 stage. **(C)** Representative examples illustrate changes in the expression levels of miRNA-pr across multiple time points in a cell. I and II denote early and later time windows, respectively. The first two cases were annotated as having increased expression, indicating that the promoter is active in the cell. **(D)** Summary of embryonic miRNA-pr expression as cell track expression. A cell track is a sequence of temporally-ordered mother-daughter cells that proceeds from early progenitor cells to individual terminal cells. The figure exemplifies parts of two cell tracks (circles linked by dashed arrows) within the cell lineage tree. miRNA expression levels are averaged across all cells with expression in a track to summarize expression during the development of each terminal cell. Cells are colored according to expression levels, and the barcode on the bottom represents averaged expression along all cell tracks. **(E)** Flow chart of the strategies to select representative expression patterns among multiple reporter strains of the same miRNA. Details on the rules are provided in the Methods. **(F, G)** Two representative examples (F for miR-1 and G for miR-232) illustrate the selection of the most representative expression patterns among all observed patterns in different reporter strains for a single miRNA.

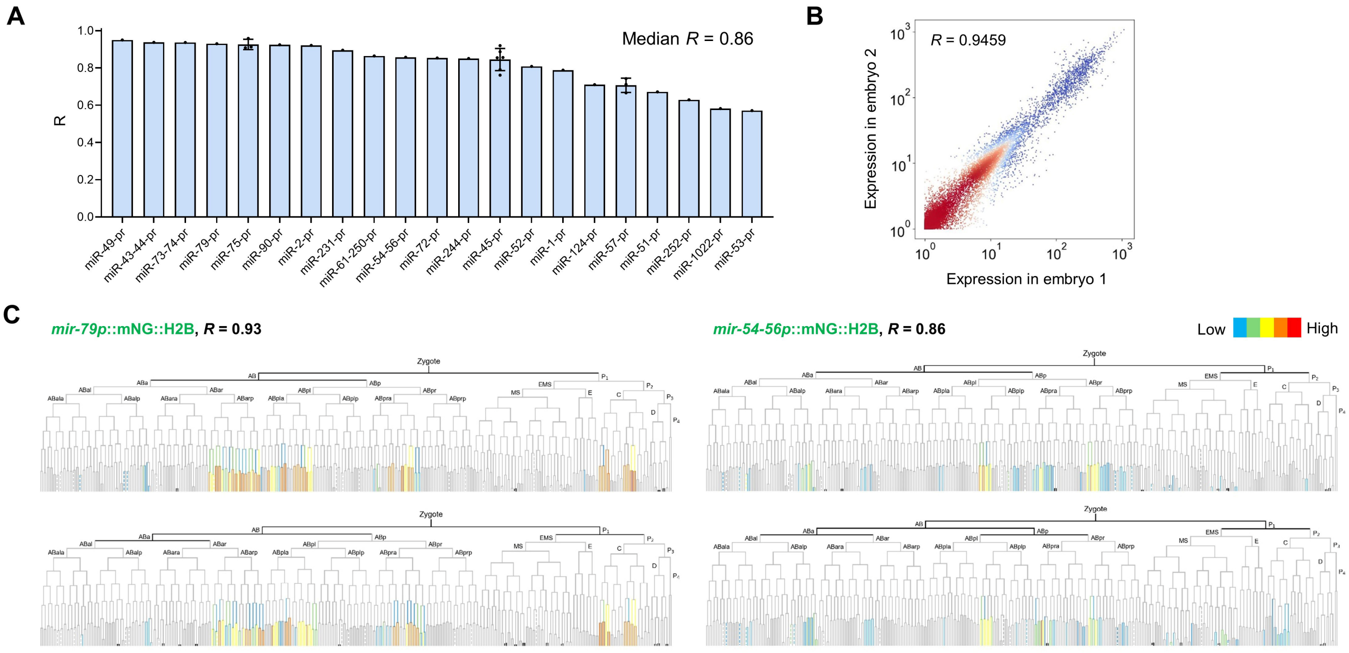

Supplementary Fig. 2. Reproducibility of miRNA expression.

(A) Pearson correlation coefficient (R) of single-cell expression between experimental replicates of the same miRNA reporter. Only reporter strains with the most representative expression and with ≥5 expressing cells in all experimental replicates were included (n = 21). Only embryos with identical orientation (dorsal or ventral side facing the objective at the 350-cell stage) were compared (30 pairs). Each dot represents a comparison. **(B)** Correlation of miRNA-pr expression in equivalent cells (dots, n = 14,935) between experimental replicates. **(C)** Representative examples show the consistency of single-cell miRNA-pr expression during embryogenesis between two experimental replicates. Source data are provided as a Source Data file.

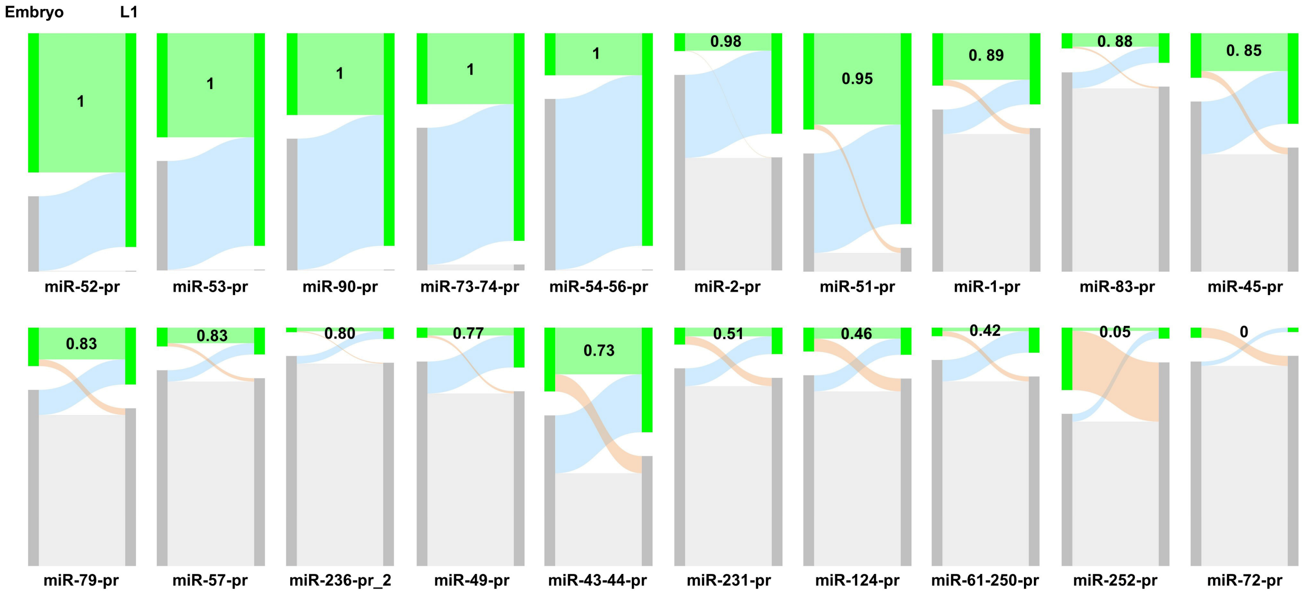

Supplementary Fig. 3. Comparison of miRNA expression between embryos and L1 larvae.

Sankey diagrams show the differences in expression status (green indicates expression and gray indicates non-expression) of each miRNA-pr in equivalent cells of the embryonic and L1 stages. Numbers indicate the fraction of traced terminal cells with miRNA expression for which the equivalent L1 cell also expressed the miRNA. Due to the cumulative nature of the long-lived mNG::H2B fluorescence signal, only cells in the terminal generation of all traced cells (leaf cells) where the promoter is defined as active were included in the comparison. Also, the analysis did not include miRNA reporters with less than 10 expressing cells. Source data are provided as a Source Data file.

A

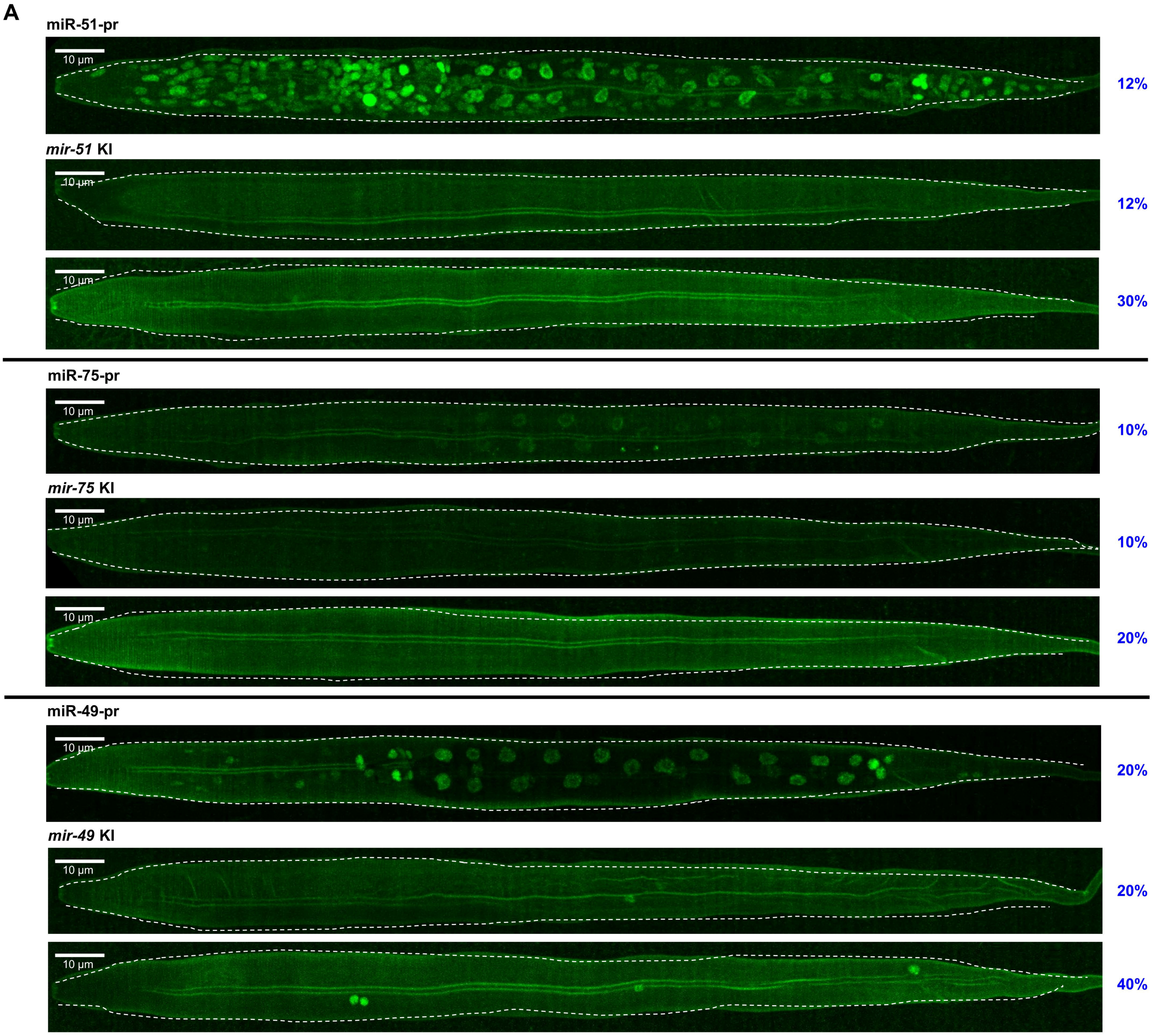

B

miRNA	miRNA-pr expression pattern in this study	miRNA KI reporter expression pattern in this study	Consistency	miRNA-pr expression pattern validated by other studies, including reporter assays or sequencing
miR-51	Widespread expression with particularly strong signals observed in the excretory canal cell	No expression detected	Not consistent	• From late embryo to L1, expression is detected in canal cells and canal nerves. Also, from late embryos to adults, expression is detected in several nerves, including dnc and vnc. In addition, expression is detected in head muscles, coelomocytes and intestine. (Martinez et al., 2008)<br>• miR-51 exhibited high expression level in small RNA-seq at L1 stage (Dexheimer et al., 2020; Kato et al., 2009)
miR-75	Exclusively expressed in intestine cells.	No expression detected	Not consistent	• Expression seen exclusively in the intestine (Martinez et al., 2008)<br>• Expression enriched in intestine (Alberti et al., 2018)
miR-49	Expressed in intestine and epidermis at the L1 stage	Expression detected only in a few unidentified cells at the L1 stage, partially overlapping with miRNA-49-pr-expressing cells	Not consistent	• miR-49 exhibited high expression level in small RNA-seq at L1 stage. (Dexheimer et al., 2020; Kato et al., 2009)

C

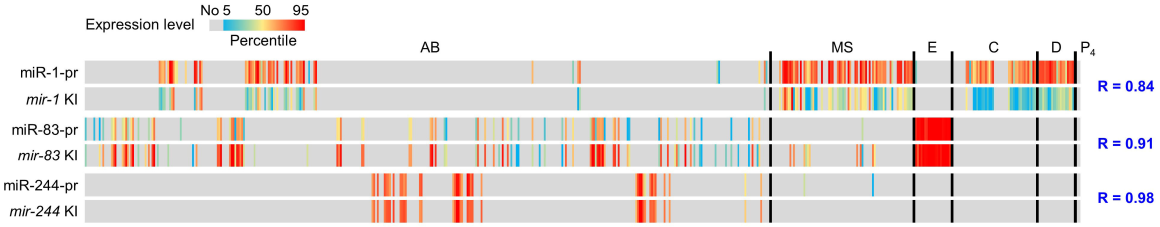

Supplementary Fig. 4. Endogenous fluorescent reporters exhibiting weak or sporadical expression.

(A) Maximum projection images showing cellular mNG::H2B intensity of promoter-driven and KI reporters for three miRNAs. The numbers shown on the right represent the laser power (expressed as a percentage of the maximum). (**B**) Comments on the comparison and supporting evidence for the reliability of the miRNA-pr expression pattern. (**C**) Comparison of cellular expression levels of three miRNA-prs with endogenous fluorescent reporters at the L1 stage. Cells are organized according to lineage, and the Pearson correlation coefficients are indicated on the right.

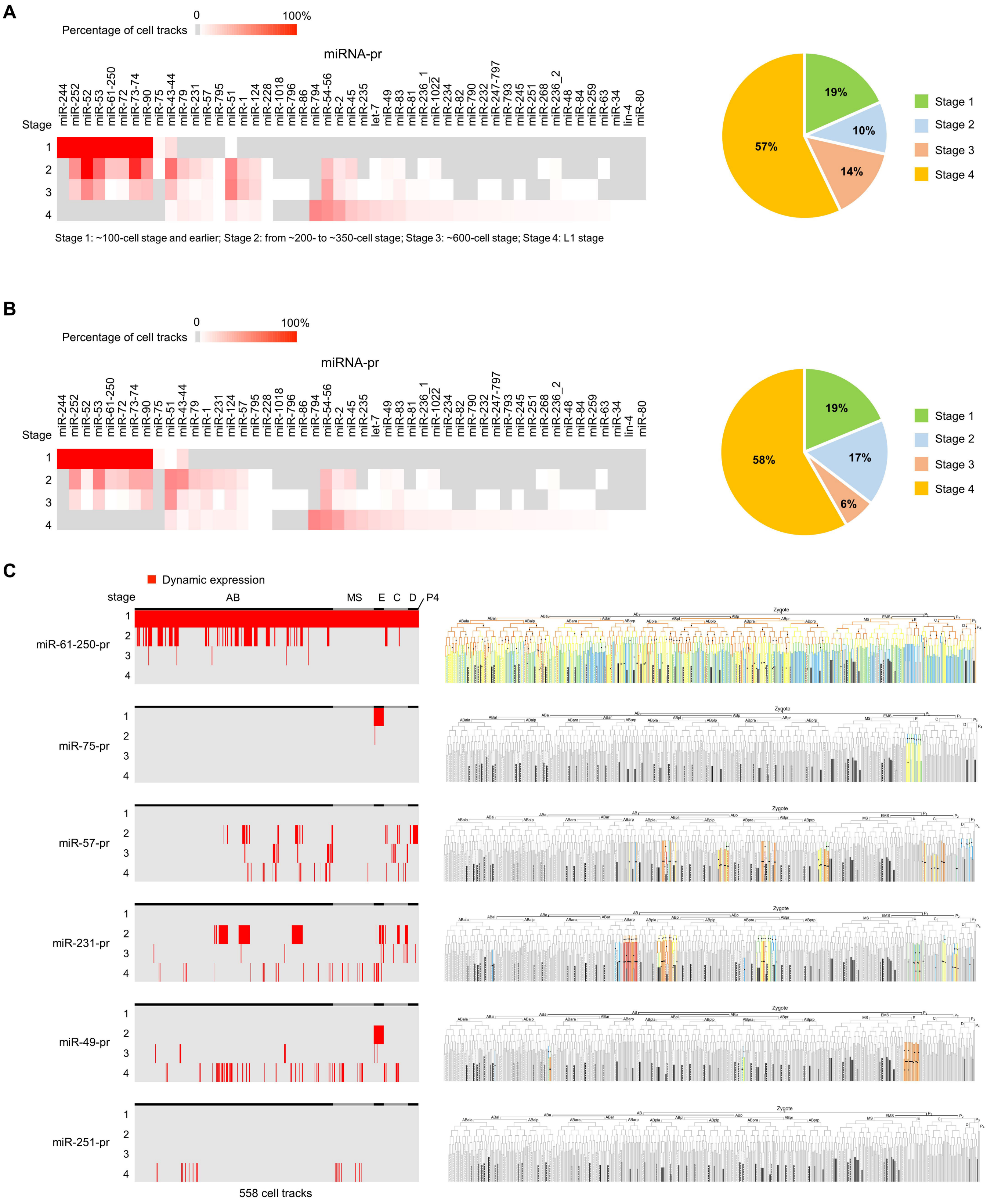
A
Percentage of cell tracks
0
100%
miRNA-pr
Stage
miR-244 miR-252 miR-52 miR-53 miR-61-250 miR-72 miR-73-74 miR-90 miR-75 miR-43-44 miR-79 miR-231 miR-57 miR-795 miR-51 miR-1 miR-124 miR-228 miR-1018 miR-796 miR-86 miR-794 miR-54-56 miR-2 miR-45 miR-235 let-7 miR-49 miR-83 miR-81 miR-236_1 miR-1022 miR-234 miR-82 miR-790 miR-232 miR-247-797 miR-793 miR-245 miR-251 miR-268 miR-236_2 miR-48 miR-84 miR-259 miR-63 miR-34 lin-4 miR-80
1
2
3
4
Stage 1: ~100-cell stage and earlier; Stage 2: from ~200- to ~350-cell stage; Stage 3: ~600-cell stage; Stage 4: L1 stage
19%
10%
14%
57%
Stage 1
Stage 2
Stage 3
Stage 4
B
Percentage of cell tracks
0
100%
miRNA-pr
Stage
miR-244 miR-252 miR-52 miR-53 miR-61-250 miR-72 miR-73-74 miR-90 miR-75 miR-51 miR-43-44 miR-79 miR-1 miR-231 miR-124 miR-57 miR-795 miR-228 miR-1018 miR-796 miR-86 miR-794 miR-54-56 miR-2 miR-45 miR-235 let-7 miR-49 miR-83 miR-81 miR-236_1 miR-1022 miR-234 miR-82 miR-790 miR-232 miR-247-797 miR-793 miR-245 miR-251 miR-268 miR-236_2 miR-48 miR-84 miR-259 miR-63 miR-34 lin-4 miR-80
1
2
3
4
19%
17%
6%
58%
Stage 1
Stage 2
Stage 3
Stage 4
C
Dynamic expression
stage
AB
MS
E
C
D
P4
miR-61-250-pr
miR-75-pr
miR-57-pr
miR-231-pr
miR-49-pr
miR-251-pr
558 cell tracks
Zygote

Supplementary Fig. 5. Temporal dynamics of miRNA.

(A) Left: Heatmap depicting the distribution of transcriptional activity of miRNA-prs (columns) across developmental stages (rows). For each miRNA-pr, cell tracks with increased fluorescence intensity were defined as exhibiting transcriptional activity. All cells were classified into four developmental stages: Stage 1, including all cells at the ~100-cell stage and earlier; Stage 2, including all cells from the ~200- to ~350-cell stage; Stage 3, including all cells at the ~600-cell stage; and Stage 4, including all cells at the L1 stage. For each stage, the fraction of cell tracks with transcriptional activity for a miRNA-pr was calculated and represented as a color in the heatmap. Right: A pie chart summarizes the distribution of transcriptional activity of miRNA-prs across developmental stages. **(B)** Left: Distribution of the onset of transcriptional activity of miRNA-prs across developmental stages. Figure organization is identical to (A), except only the earliest cell (onset cell) on a cell track exhibiting transcriptional activity of a miRNA-pr was used for the analysis. Right: A pie chart summarizes the distribution of the onset of transcriptional activity of miRNA-prs across developmental stages. **(C)** Left: Transcriptional activity of representative miRNA-prs across cell tracks (columns) at different developmental stages (rows). Right: tree visualization of cellular expression of corresponding miRNA-prs. Source data are provided as a Source Data file.

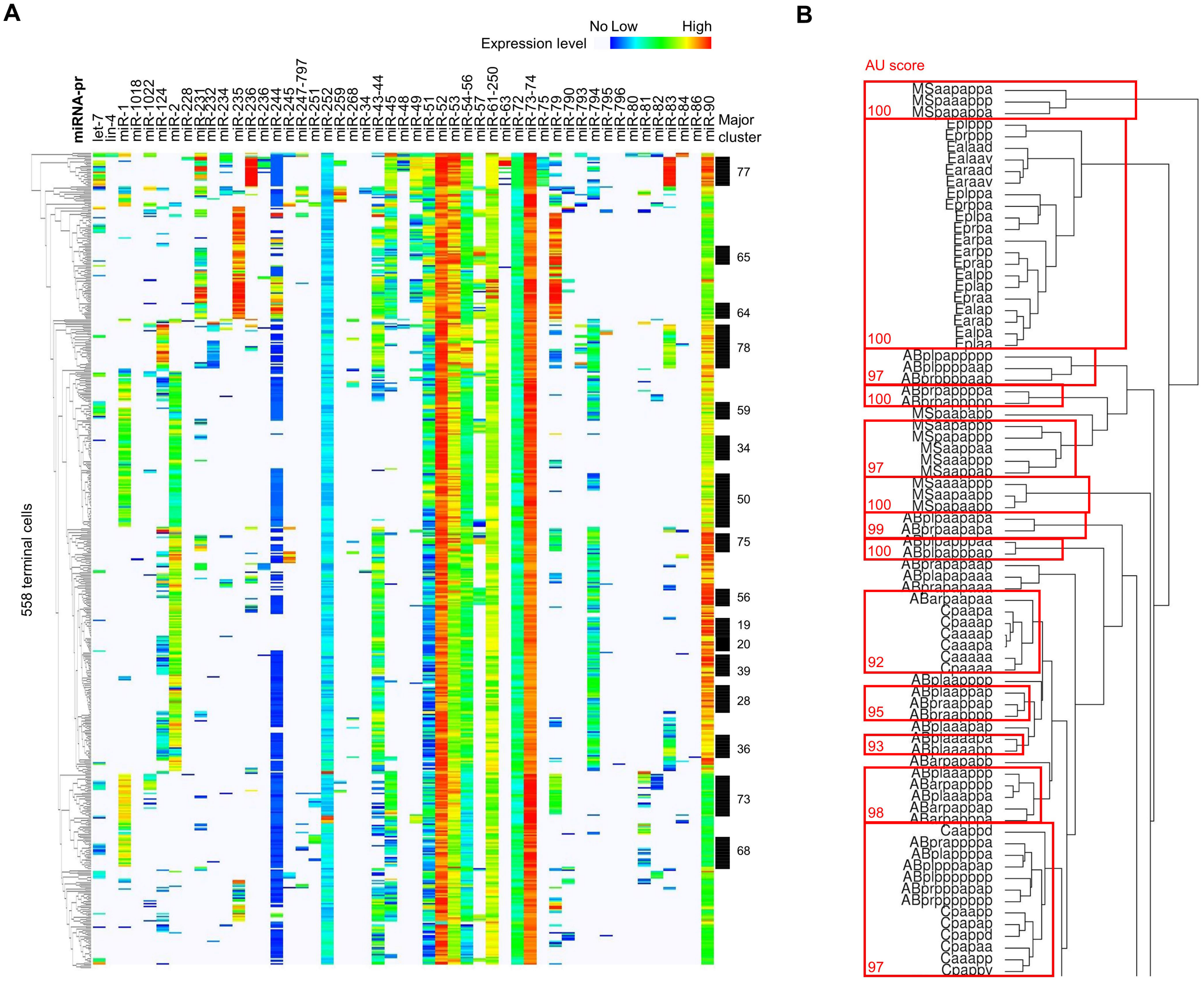

Supplementary Fig. 6. Clustering of cells based on miRNA expression.

(A) Heatmap illustrating the expression of each miRNA-pr (column) during the development of each terminal cell. The maximum value of the miRNA-pr's relative expression (percentile rank) in the cell track leading to each terminal cell and in the equivalent cell at the L1 stage was used. The dendrogram on the left shows cells (rows) clustered by miRNA-pr expression, and the bars on the right highlight cell clusters with 10 or more cells. **(B)** An example demonstrates the identification of cell clusters (boxes) using the Pvclust approach based on the AU (approximately unbiased) score (≥90 as the cutoff).

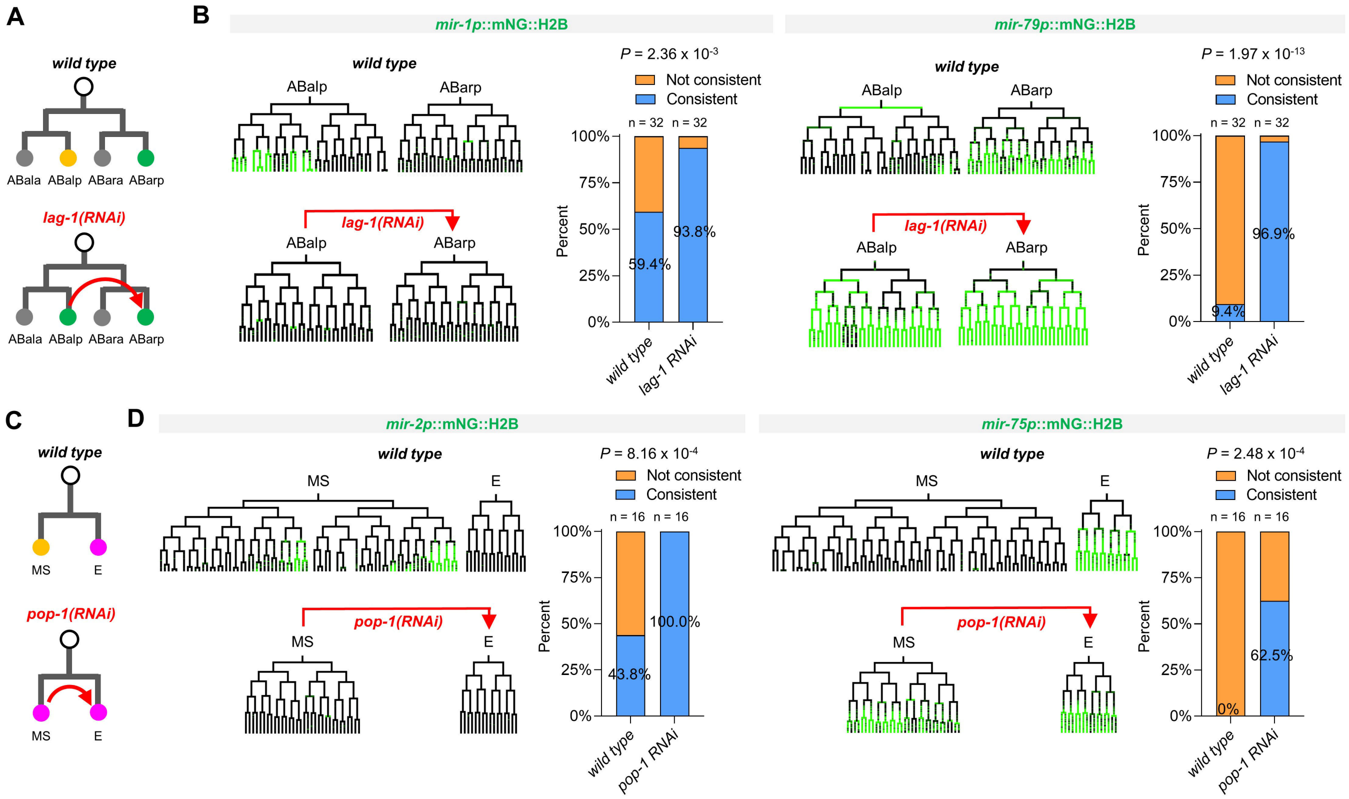

Supplementary Fig. 7. Association of miRNA expression with progenitor cell fate.

(A) Transformation of the developmental fate (indicated by color) of the ABalp cell to that of ABarp by performing RNAi against *lag-1*. **(B)** Expression of miR-1-pr (left) and miR-79-pr (right) in cells from ABalp and ABarp lineages before (top) and after (bottom) performing RNAi against *lag-1*. Bar graphs compare the expression consistency (binary expression) of corresponding miRNA-prs in lineage-equivalent leaf cells between ABalp and ABarp lineages, before and after performing RNAi against *lag-1*. In cases where two progenitor cells produce different numbers of leaf cells, the smaller lineage is expanded to match the larger one, and cells and expression status are expanded in the corresponding lineage branches. Statistics: Fisher's exact test, two-tailed. **(C)** Transformation of the developmental fate (indicated by color) of the MS progenitor cell to that of E by performing RNAi against *pop-1*. **(D)** Expression of miR-2-pr (left) and miR-75-pr (right) reporters in cells from MS and E lineages before (top) and after (bottom) performing RNAi against *pop-1*. Bar graphs compare the expression consistency of corresponding miRNA-prs in lineage-equivalent leaf cells between ABalp and ABarp lineages before and after performing RNAi against *pop-1*. Statistics: Fisher's exact test, two-tailed. Source data are provided as a Source Data file.

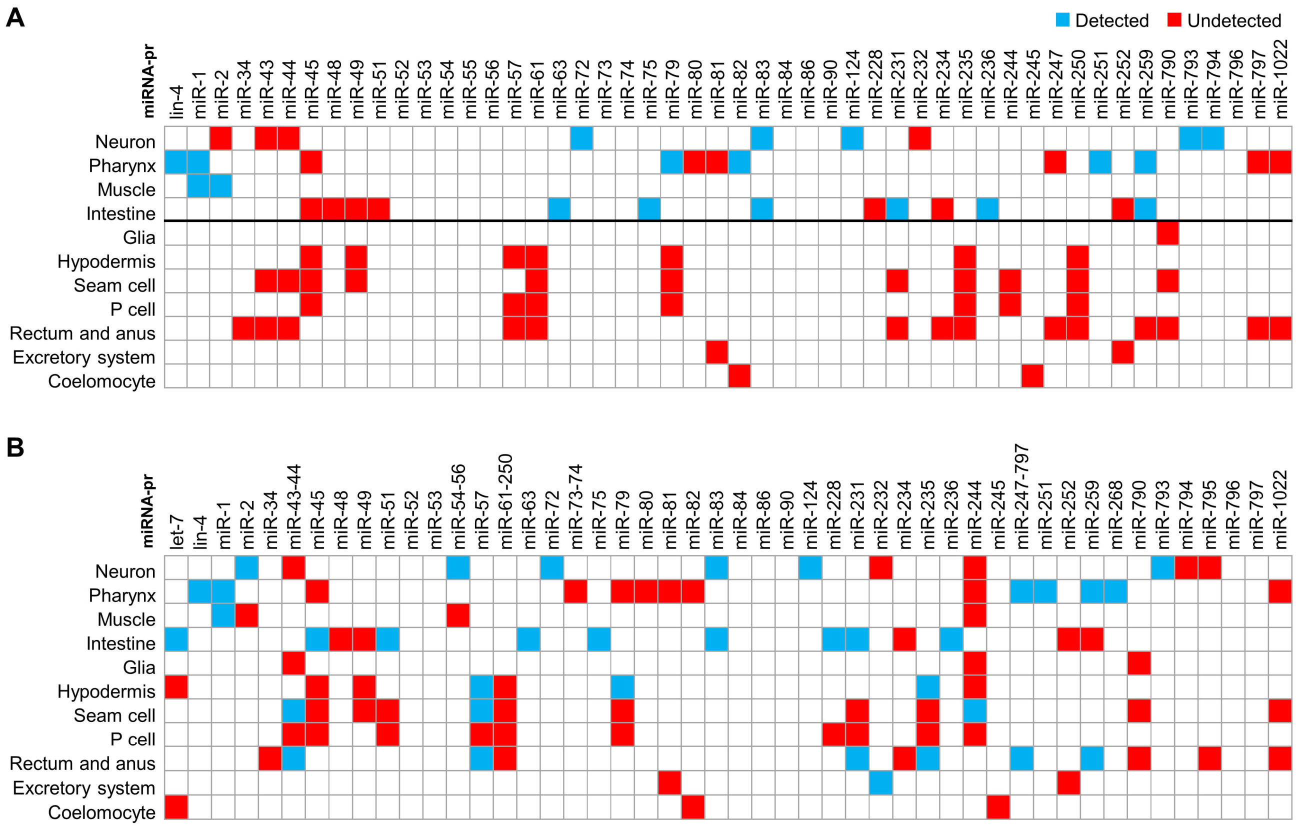

Supplementary Fig. 8. Detection status of tissue-enriched miRNA expression identified in this study.

Heatmap showing the detection status of all tissue-enriched miRNA-pr expression identified in this study in previous studies. (**A**) illustrates the comparison of this study to a previous tissue-level miRNA-seq study at the L1 stage [1]. If a tissue-enrichment pattern identified at the L1 stage in this study was also documented in the previous study, it was classified as detected. (**B**) depicts the comparison of this study to several previous studies using other approaches [1-10]. Tissue enrichments observed in both embryos and at the L1 stage in this study were used for comparison. For each tissue-enrichment pattern, if the previous study explicitly described a miRNA as expressed (weak expression was not included) in this tissue, this pattern was classified as detected. In cases where the previous study described the expression in specific cells, we manually checked whether the described cell list overlapped with our cellular expression and classified the pattern as detected if a considerable overlap was observed. Otherwise, the pattern was classified as undetected.

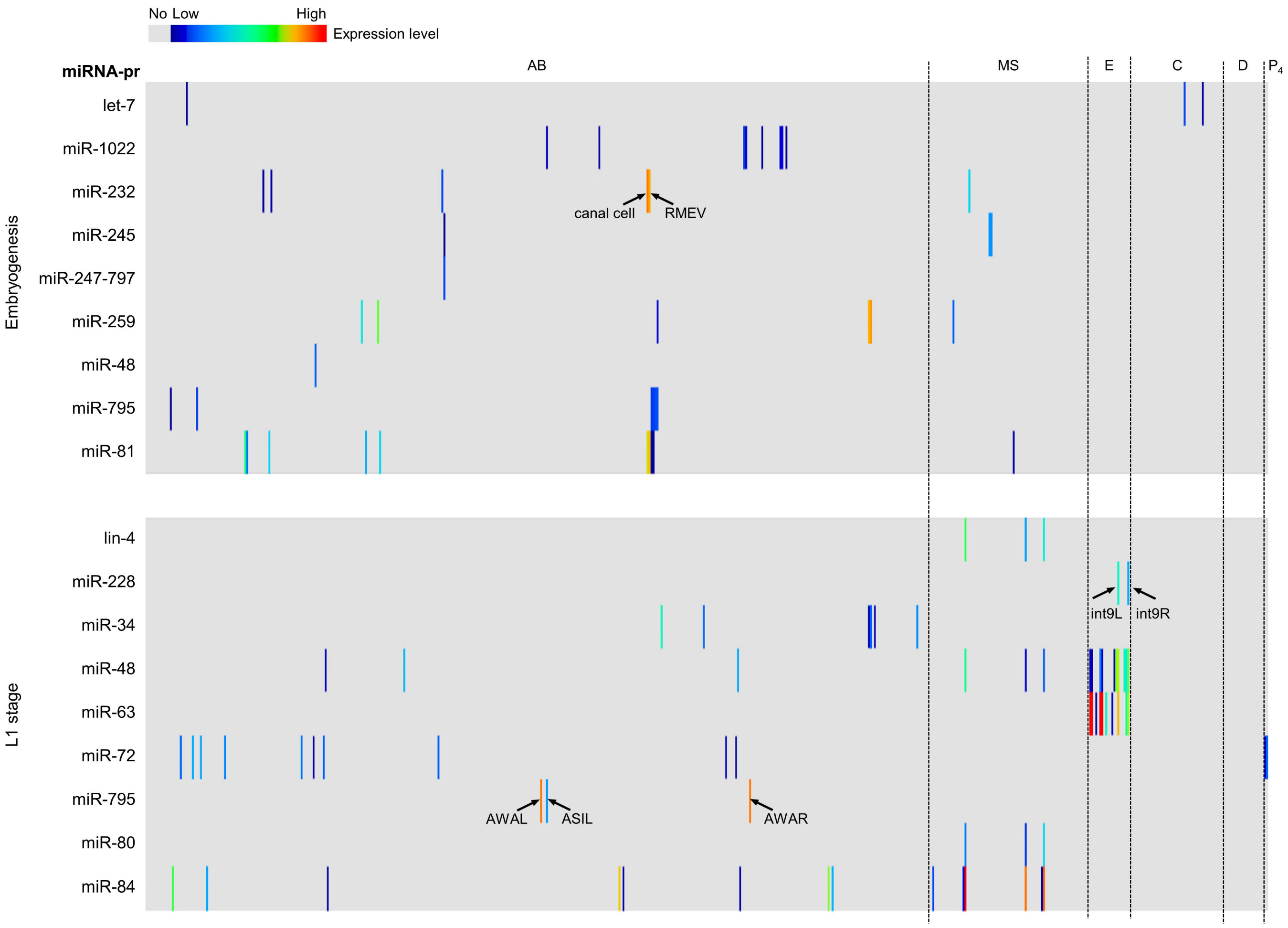

Supplementary Fig. 9. Expression specificity of miRNA.

Expression levels of miRNA-prs (rows) with highly restricted expression (expressed in <20 cell tracks in the embryos or in <20 cells at the L1 stage) in individual cell tracks (columns) during embryogenesis (top) or in individual cells (columns) at the L1 stage (bottom). Cells and tracks are ordered by lineage.

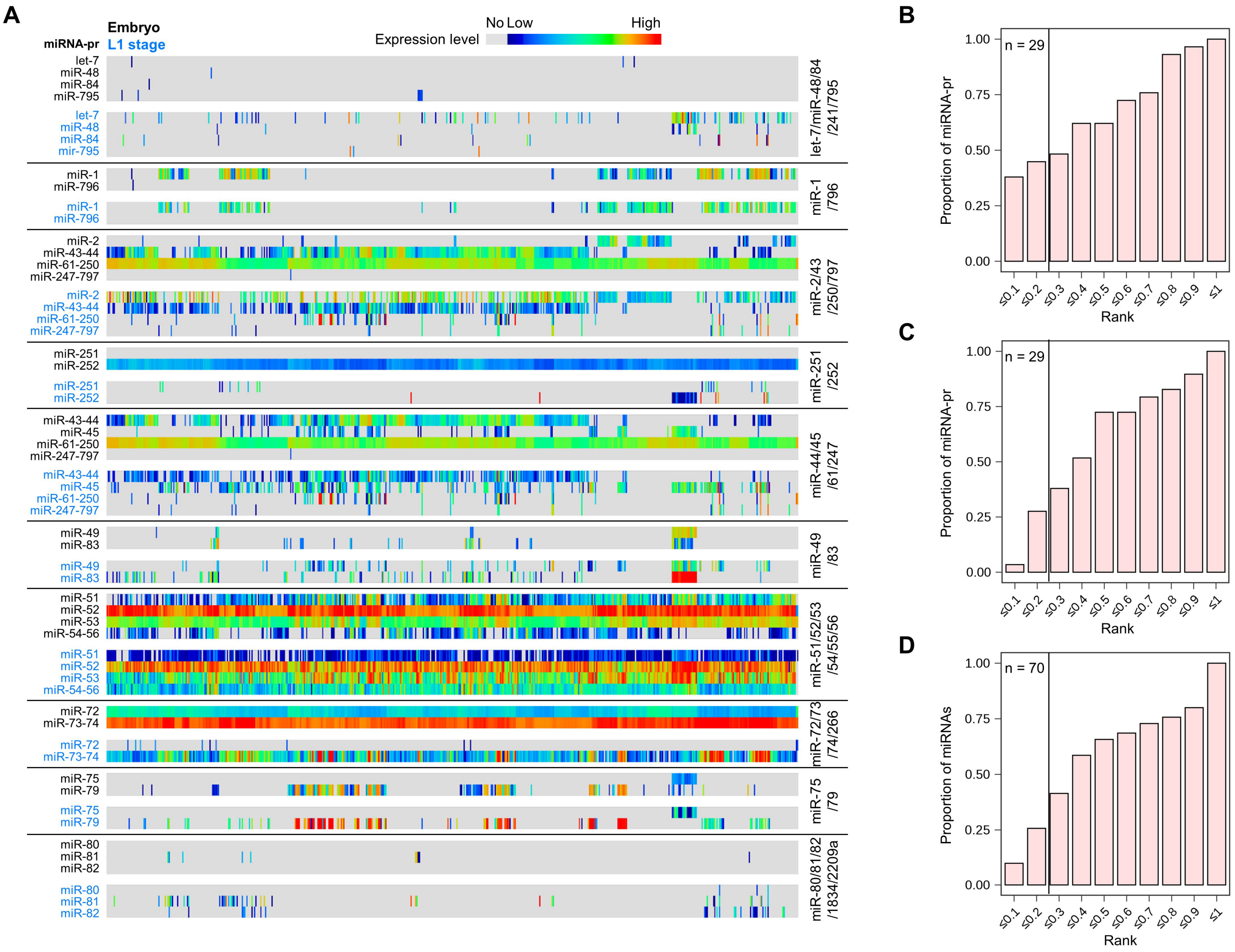

Supplementary Fig. 10. Divergent expression of miRNAs among family members.

(A) Comparison of the expression of individual miRNA-prs of a family in each cell track during embryogenesis or in each cell at the L1 stage. Cell tracks or terminal cells are ordered by cell lineage. **(B-C)** Cumulative distribution of the expression similarity among intra-family miRNA-prs relative to that among inter-family miRNA-prs in embryos (B) and at the L1 stage (C). For each miRNA family member, we first calculated the divergence of cellular miRNA-pr expression among all miRNA members in the same family as 1 minus Spearman's rank correlation coefficient (1-ρ). Then, we measured the expression divergence of this miRNA-pr to all other miRNA-prs not in the same family. Finally, the percentile rank of the averaged intra-family miRNA-pr expression divergence relative to inter-family miRNA-pr expression divergence was calculated to measure the relative expression divergence of miRNA-prs in a family. A lower percentile rank indicates a higher expression similarity. **(D)** Cumulative distribution of the expression similarity among intra-family miRNAs relative to that among inter-family miRNAs using tissue-level miRNA-seq data at the L1 stage. The expression divergence between miRNAs was measured as the Root Mean Square Deviation of expression levels (converted to internal ranks across datasets) among six datasets for four tissues. Source data are provided as a Source Data file.

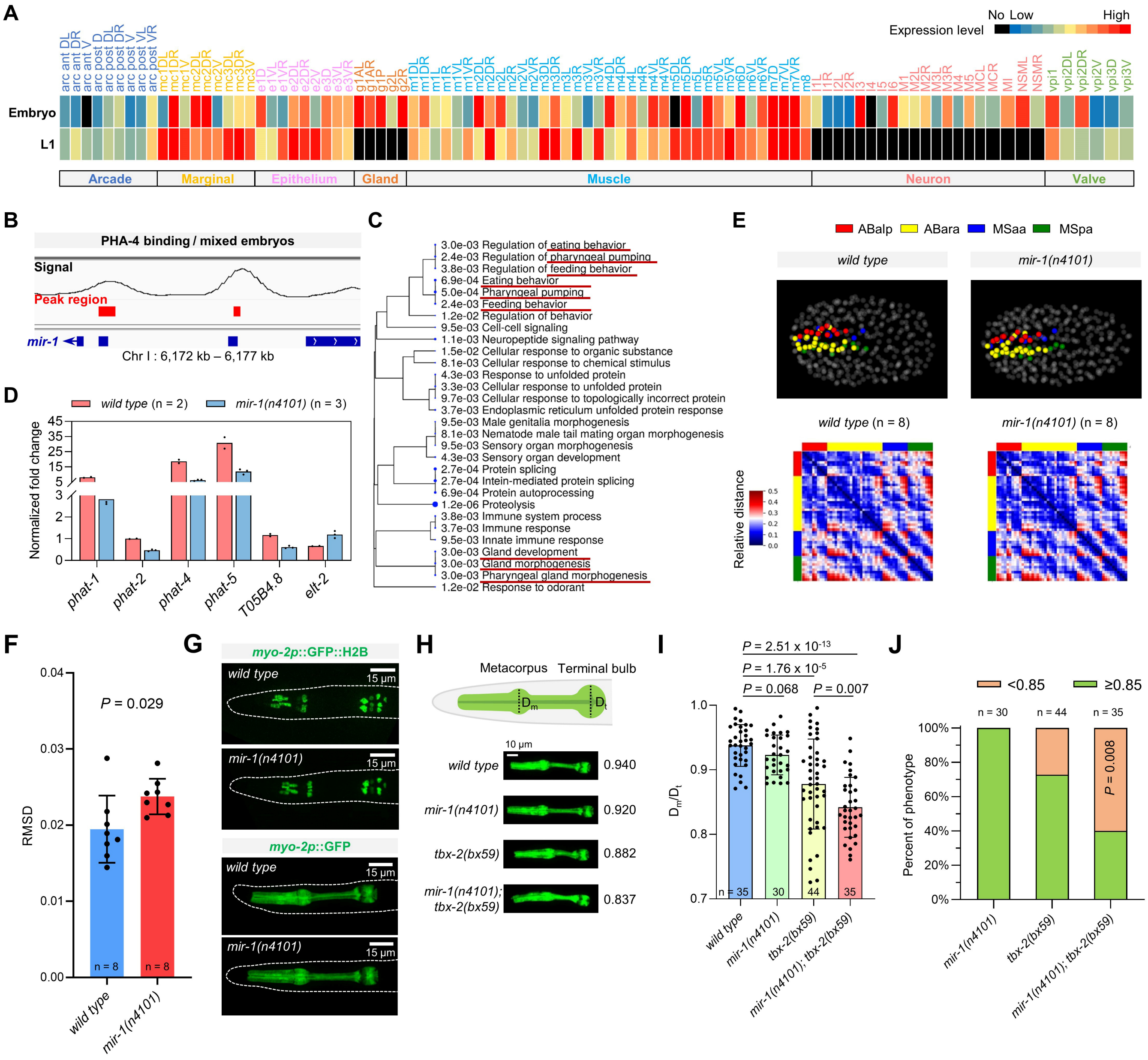

Supplementary Fig. 11. Functional analysis of miR-1.

(A) Expression of miR-1-pr in pharyngeal cells in bean-stage embryos and L1 larvae. **(B)** PHA-4 binding at the *mir-1* promoter. Red boxes indicate the peak regions. **(C)** Significantly enriched Gene Ontology terms, with red underscores highlighting those related to the pharynx. Gene Ontology analysis was performed by ShinyGO using default parameters [11]. **(D)** Quantitative RT-PCR comparing the expression of five gland-related genes and negative control (*elt-2*) relative to *ubc-2* between wild-type and *mir-1(n4101)* embryos. Each bar represents the mean value. **(E)** Comparison of the 3D position of pharyngeal cells between wild-type and *mir-1(n4101)* embryos at the 350-cell stage. Top: 3D rendering of pharyngeal cell positions (approximated by nucleus position). Cells are color-coded according to lineage origin. Bottom: Heatmap showing the averaged pair-wise geometric distances (normalized to the embryo length) between cells. **(F)** Comparison of the differences in the distance matrix (quantified as root-mean-square deviation, RMSD) of each wild-type or *mir-1(n4101)* embryo to that of the averaged wild-type embryos.

Statistics: *t*-test, two-tailed, unpaired. **(G)** Comparison of pharynx morphology between wild-type and *mir-1(n4101)* at the L1 stage using nucleus-localized (top) or ubiquitous-localized (bottom) GFP driven by the *myo-2* promoter. **(H)** Top: Quantification of the diameters of the metacorpus (D_m) and terminal bulb (D_t) at the L1 stage. Bottom: Micrographs comparing pharynx morphology (visualized using single-copy *myo-2* promoter-driven GFP) between single and double mutants of *mir-1* and *tbx-2*. The numbers show the median ratio of D_m to D_t. **(I)** Comparison of D_m/D_t values between genotypes. Data are shown as boxplots with whiskers drawn down to the minimum and up to the maximum value. Each dot represents the result of an animal. Data are represented as mean ± SD. Statistics: Mann-Whitney U test, two-tailed. **(J)** Distribution of cases exhibiting a severe defect in pharynx morphology (defined as D_m/D_t <0.85) in single and double mutants of *mir-1* and *tbx-2*. *P*-values were calculated by comparing the observed frequency to that predicted based on additive effects of single mutants using a two-tailed Chi-square test. Source data are provided as a Source Data file.

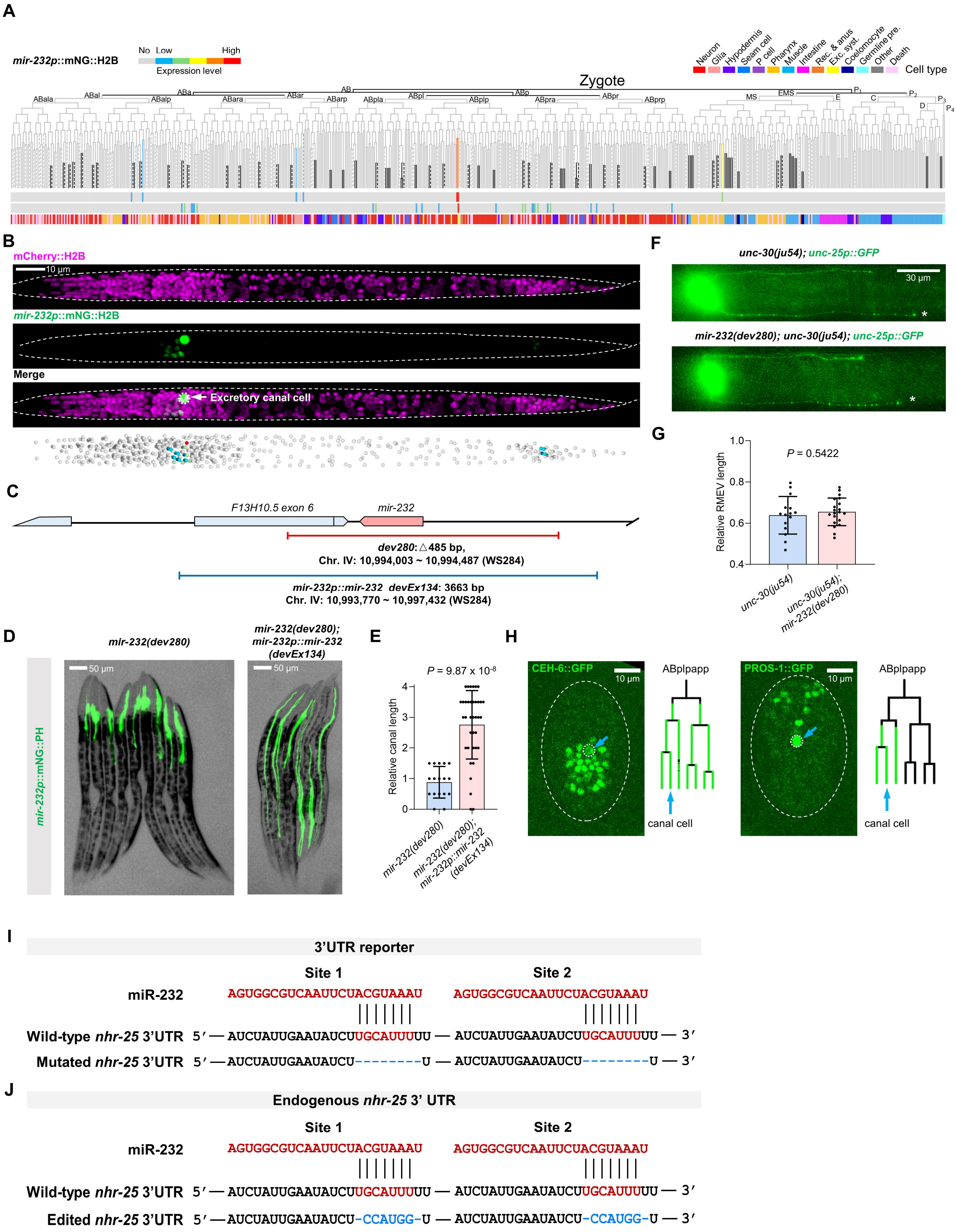
A
mir-232p::mNG::H2B
No Low High
Expression level
Neuron
Glia
Hypodermis
Seam cell
P cell
Pharynx
Muscle
Intestine
Rec. & anus
Exc. syst.
Coelomocyte
Germline pre.
Other
Death
Cell type
Zygote
B
mCherry::H2B
10 μm
mir-232p::mNG::H2B
Merge
Excretory canal cell
C
F13H10.5 exon 6
mir-232
dev280:△485 bp,
Chr. IV: 10,994,003 ~ 10,994,487 (WS284)
mir-232p::mir-232 devEx134: 3663 bp
Chr. IV: 10,993,770 ~ 10,997,432 (WS284)
D
mir-232(dev280)
mir-232(dev280); mir-232p::mir-232 (devEx134)
mir-232p::mNG::PH
50 μm
E
P = 9.87 x 10-8
Relative canal length
mir-232(dev280)
mir-232(dev280); mir-232p::mir-232 (devEx134)
F
unc-30(ju54); unc-25p::GFP
30 μm
mir-232(dev280); unc-30(ju54); unc-25p::GFP
G
P = 0.5422
Relative RMEV length
unc-30(ju54)
unc-30(ju54); mir-232(dev280)
H
CEH-6::GFP
10 μm
ABplpapp
canal cell
PROS-1::GFP
10 μm
ABplpapp
canal cell
I
3'UTR reporter
Site 1
Site 2
miR-232
AGUGGCGUCAAUUCUACGUAAAU
AGUGGCGUCAAUUCUACGUAAAU
Wild-type nhr-25 3'UTR 5′ — AUCUAUUGAAUAUCUUGCAUUUUU — AUCUAUUGAAUAUCUUGCAUUUUU — 3′
Mutated nhr-25 3'UTR 5′ — AUCUAUUGAAUAUCU--------U — AUCUAUUGAAUAUCU--------U — 3′
J
Endogenous nhr-25 3' UTR
Site 1
Site 2
miR-232
AGUGGCGUCAAUUCUACGUAAAU
AGUGGCGUCAAUUCUACGUAAAU
Wild-type nhr-25 3'UTR 5′ — AUCUAUUGAAUAUCUUGCAUUUUU — AUCUAUUGAAUAUCUUGCAUUUUU — 3′
Edited nhr-25 3'UTR 5′ — AUCUAUUGAAUAUCU-CCAUGG-U — AUCUAUUGAAUAUCU-CCAUGG-U — 3′

Supplementary Fig. 12. Functional analysis of miR-232.

(A) Expression of miR-232-pr during embryogenesis. **(B)** Cellular expression of the miR-232 reporter at the L1 stage. **(C)** Schematic representation of the *mir-232(dev280)* deletion allele and the fragment used in the rescue experiment. **(D)** Comparison of excretory canal length in *mir-232(dev280)* animals before and after injecting a fragment containing the entire wild-type *mir-232* locus. Only animals with co-injection markers were used for calculation. **(E)** Quantification of excretory canal length in *mir-232(dev280)* animals before (n = 17 animals) and after (n = 37 animals) injecting the rescuing fragment. Each dot shows the length observed in an L4-stage animal. Statistics: Mann-Whitney U test, two-tailed. **(F)** Representative micrographs showing the morphology of the RMEV neuron (star, visualized using *unc-25p*::GFP) before and after miR-232 loss. **(G)** Comparison of the length of the RMEV neuron before (n = 15 animals) and after (n = 20 animals) miR-232 loss. We quantified two lengths at the L4 stage: the distance from the RMEV cell body to the vulva ($L_{body-vulva}$) and the extent of the RMEV ventral axon extension ($L_{ventral-extension}$). Subsequently, the ratio of $L_{ventral-extension}$ to $L_{body-vulva}$ was measured to determine the relative length of the RMEV ventral axon. Each dot shows the length observed in one animal. Statistics: Mann-Whitney U test, two-tailed. **(H)** Micrographs show cellular expression of CEH-6 and PROS-1 in bean-stage embryos, with arrows indicating the excretory canal cell. Cell lineage diagrams on the right show the expression (green) of the proteins in related cell lineages that give rise to the excretory canal cell. **(I)** Top: Location of miR-232 sites in the *nhr-25* 3'UTR. Vertical lines indicate complementarity of the miR-232 seed sequence to the binding site. Bottom: Sequence of the mutated *nhr-25* 3'UTR used in the 3'UTR reporter assay, in which the two miR-232 binding sites were removed. **(J)** Modified sequence of the endogenous *nhr-25* 3'UTR, in which two miR-232 binding sites were mutated by CRISPR/Cas-9-mediated genome editing. Source data are provided as a Source Data file.

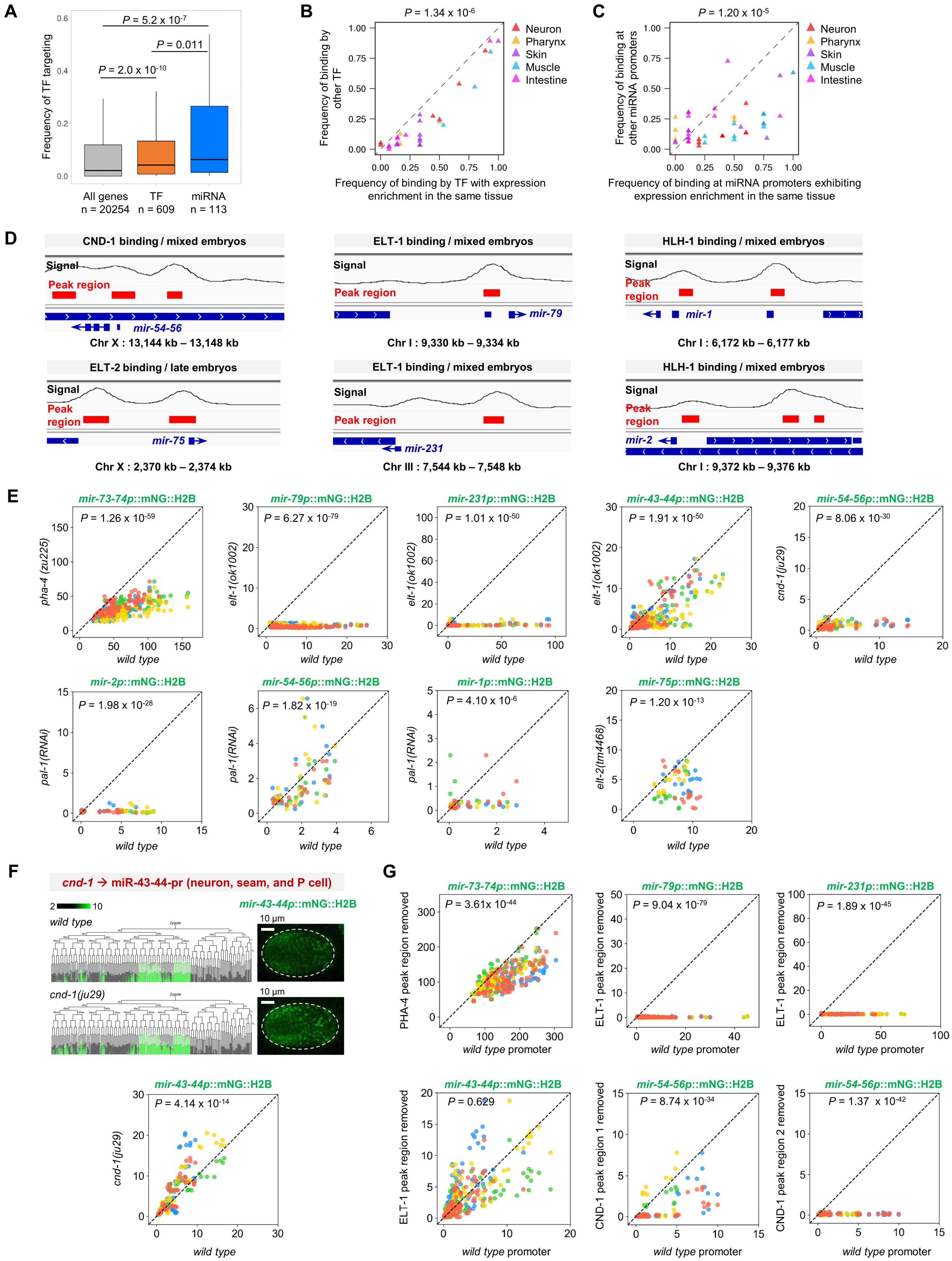
A
P = 5.2 x 10-7
P = 0.011
P = 2.0 x 10-10
Frequency of TF targeting
All genes n = 20254
TF n = 609
miRNA n = 113
B
P = 1.34 x 10-6
Frequency of binding by other TF
Frequency of binding by TF with expression enrichment in the same tissue
Neuron
Pharynx
Skin
Muscle
Intestine
C
P = 1.20 x 10-5
Frequency of binding at other miRNA promoters
Frequency of binding at miRNA promoters exhibiting expression enrichment in the same tissue
D
CND-1 binding / mixed embryos
ELT-1 binding / mixed embryos
HLH-1 binding / mixed embryos
Signal
Peak region
mir-54-56
mir-79
mir-1
Chr X : 13,144 kb – 13,148 kb
Chr I : 9,330 kb – 9,334 kb
Chr I : 6,172 kb – 6,177 kb
ELT-2 binding / late embryos
ELT-1 binding / mixed embryos
HLH-1 binding / mixed embryos
mir-75
mir-231
mir-2
Chr X : 2,370 kb – 2,374 kb
Chr III : 7,544 kb – 7,548 kb
Chr I : 9,372 kb – 9,376 kb
E
mir-73-74p::mNG::H2B P = 1.26 x 10-59 pha-4 (zu225)
mir-79p::mNG::H2B P = 6.27 x 10-79 elt-1(ok1002)
mir-231p::mNG::H2B P = 1.01 x 10-50 elt-1(ok1002)
mir-43-44p::mNG::H2B P = 1.91 x 10-50 elt-1(ok1002)
mir-54-56p::mNG::H2B P = 8.06 x 10-30 cnd-1(ju29)
mir-2p::mNG::H2B P = 1.98 x 10-28 pal-1(RNAi)
mir-54-56p::mNG::H2B P = 1.82 x 10-19 pal-1(RNAi)
mir-1p::mNG::H2B P = 4.10 x 10-6 pal-1(RNAi)
mir-75p::mNG::H2B P = 1.20 x 10-13 elt-2(tm4468)
wild type
F
cnd-1 → miR-43-44-pr (neuron, seam, and P cell)
mir-43-44p::mNG::H2B
wild type
cnd-1(ju29)
10 µm
mir-43-44p::mNG::H2B P = 4.14 x 10-14 cnd-1(ju29)
G
mir-73-74p::mNG::H2B P = 3.61x 10-44 PHA-4 peak region removed
mir-79p::mNG::H2B P = 9.04 x 10-79 ELT-1 peak region removed
mir-231p::mNG::H2B P = 1.89 x 10-45 ELT-1 peak region removed
mir-43-44p::mNG::H2B P = 0.629 ELT-1 peak region removed
mir-54-56p::mNG::H2B P = 8.74 x 10-34 CND-1 peak region 1 removed
mir-54-56p::mNG::H2B P = 1.37 x 10-42 CND-1 peak region 2 removed
wild type promoter

Supplementary Fig. 13. Influence of fate determinants and their binding on miRNA expression.

(A) Comparison of the binding frequency of TFs at the promoter regions of different gene types. Statistics: Mann-Whitney U test, two-tailed. **(B)** Scatter plot comparing the frequency of each pan-tissue specific miRNA promoter bound by TFs exhibiting the same tissue expression specificity (X-axis) to that bound by other TFs (Y-axis). Each triangle (n = 31) represents a pan-tissue-specific miRNA (determined by corresponding miRNA-pr) colored by tissue type. The dashed diagonal indicates equality of X and Y. Statistical analysis: Wilcoxon signed-rank test, two-tailed. **(C)** Scatter plot comparing the binding frequency of each pan-tissue-specific TF at the promoter of miRNA whose reporter exhibits the same type of pan-tissue specificity (X-axis) to that at other miRNA promoters (Y-axis). Each triangle represents a pan-tissue-specific TF (n = 50), colored by tissue. The dashed diagonal indicates equality of X and Y. Statistical analysis: Wilcoxon signed-rank test, two-tailed. **(D)** Representative examples show the binding of fate determinants at the promoters of tissue-specific miRNAs. Red boxes indicate peak regions. **(E)** Influence of fate determinants on miRNA-pr expression. Each panel compares the expression of a miRNA-pr in equivalent cells (dots) of embryos from wild-type animals (X-axis, n = 2 embryos) and those in which the indicated fate determinants were perturbed (Y-axis, n = 2 embryos). The dashed line indicates equality of X and Y. Only wild-type/mutant embryo pairs with identical orientation were compared; different colors indicate different pairs. Only cell tracks co-expressing the indicated miRNA-pr and fate determinants were included. Statistics: Wilcoxon rank sum test, two-tailed.. **(F)** Top: Changes in miR-43-44-pr expression during embryogenesis in *cnd-1(ju29)* embryos. The micrographs are maximum projection images comparing miR-43-44 reporter expression at the 350-cell stage.. Bottom: Comparison of miR-43-44 reporter expression in the wild-type (X-axis) and *cnd-1(ju29)* embryos (Y-axis) in equivalent cells (dots). Scatter plot organization and statistics are identical to (E). **(G)** Influence of the binding of fate determinants on miRNA-pr expression. Figure organization and statistics are identical to (E).

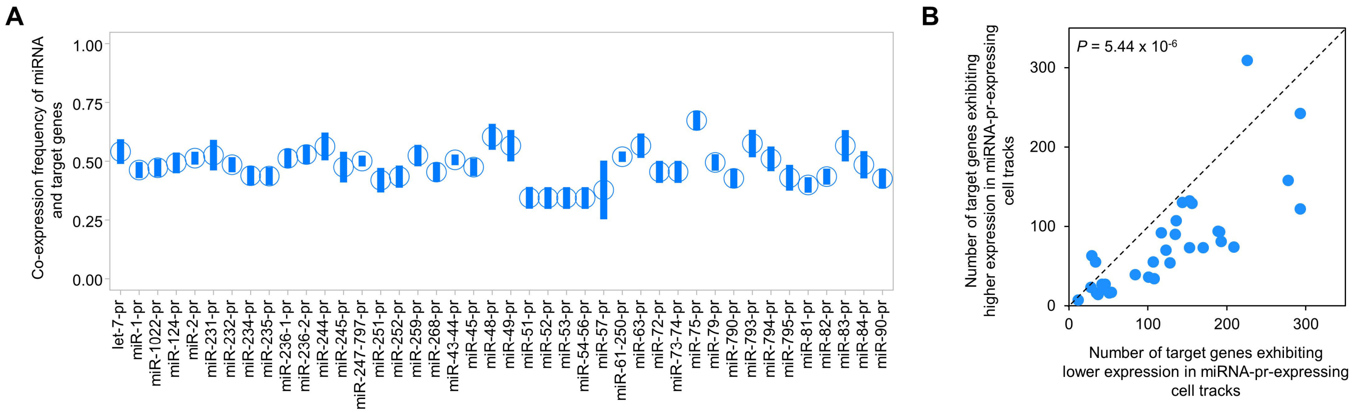

Supplementary Fig. 14. Expression status of miRNAs and their targets

(A) Frequencies with which miRNA-prs are expressed in cell tracks also expressing corresponding target genes. Only miRNA-prs expressed in ≥10 cell tracks were included. The value of each miRNA target was calculated, and the results were averaged across all targets (n = 209, 549, 473, 303, 828, 141, 685, 325, 429, 384, 384, 176, 149, 1138, 252, 252, 294, 401, 1,138, 354, 209, 137, 241, 241, 241, 241, 42, 1,138, 325, 247, 247, 467, 467, 356, 209, 241, 209, 618, 618, 137, 209, and 321 genes, from left to right). The data is summarized as an open circle indicating the mean and a vertical bar indicating the 95% confidence interval. **(B)** Scatter plot comparing the number of target genes exhibiting lower expression in miRNA-pr-expressing cell tracks than in non-expressing cell tracks (X-axis) to the number of target genes exhibiting the opposite pattern (Y-axis). Each dot represents the result of a miRNA. To ensure reliable comparison, only miRNA-prs expressed in between 10 and 500 cell tracks were analyzed. The dashed diagonal indicates equality of X and Y. Statistics: Wilcoxon signed-rank test, two-tailed. Source data are provided as a Source Data file.

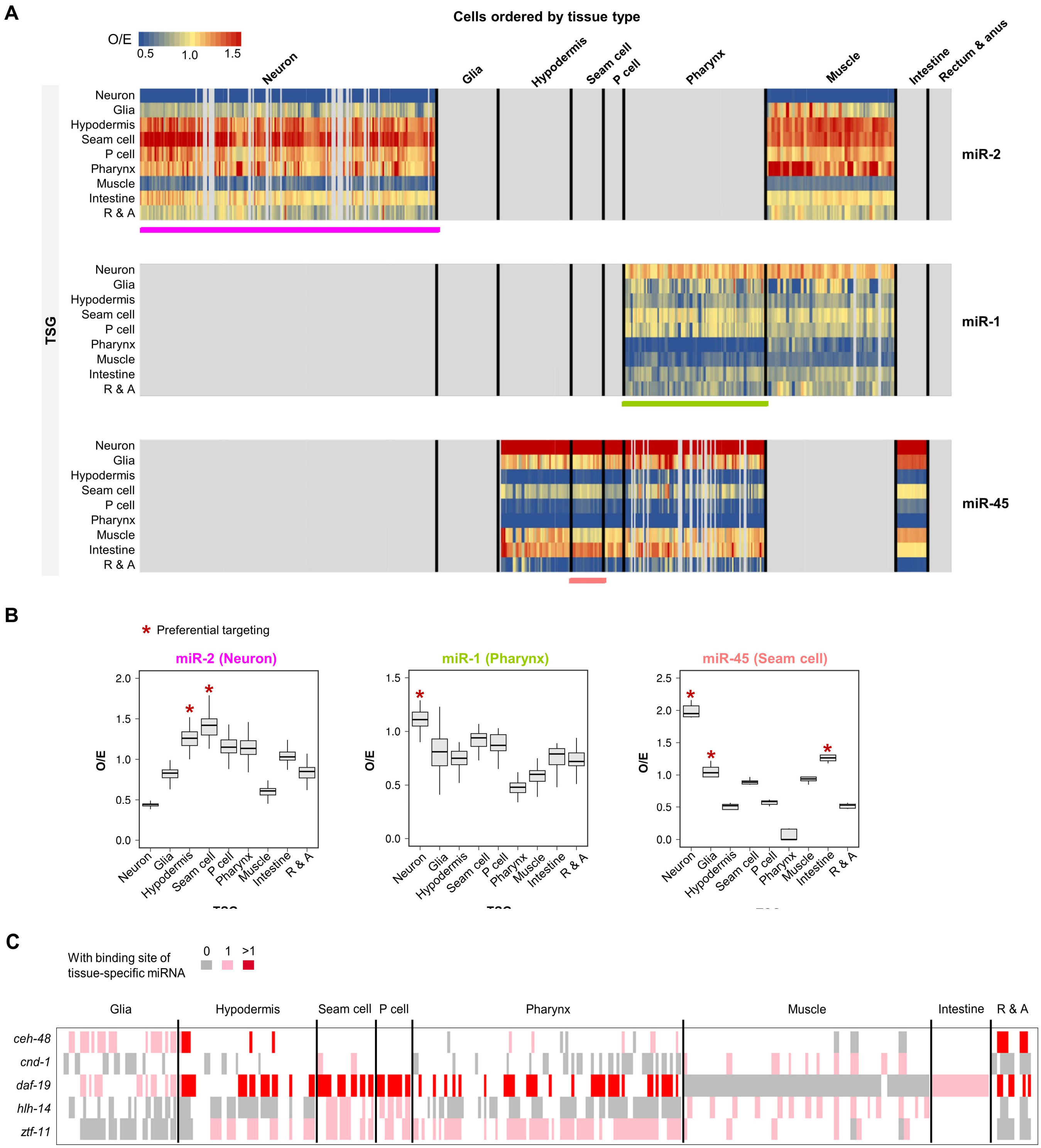

Supplementary Fig. 15. Preferential targeting of miRNAs against TSGs

(A) Preferential targeting of three representative miRNAs against TSGs associated with different tissue types (rows) in each cell track (columns, ordered by cell type). Gray indicates that miRNA is not expressed in the cell track. **(B)** Boxplots compare preferential targeting of representative miRNAs against different

types of TSG (n = 454, 188, 547, 898, 695, 229, 1,070, 3,374, and 132 genes from left to right) in indicated tissues (corresponding to the cells marked by color bars below the heatmap).**(C)** Heatmap showing the presence of binding sites for tissue-specific miRNAs that are also expressed in corresponding cell tracks (columns) in the 3'UTRs of five known neuronal fate regulators (rows). Source data are provided as a Source Data file.

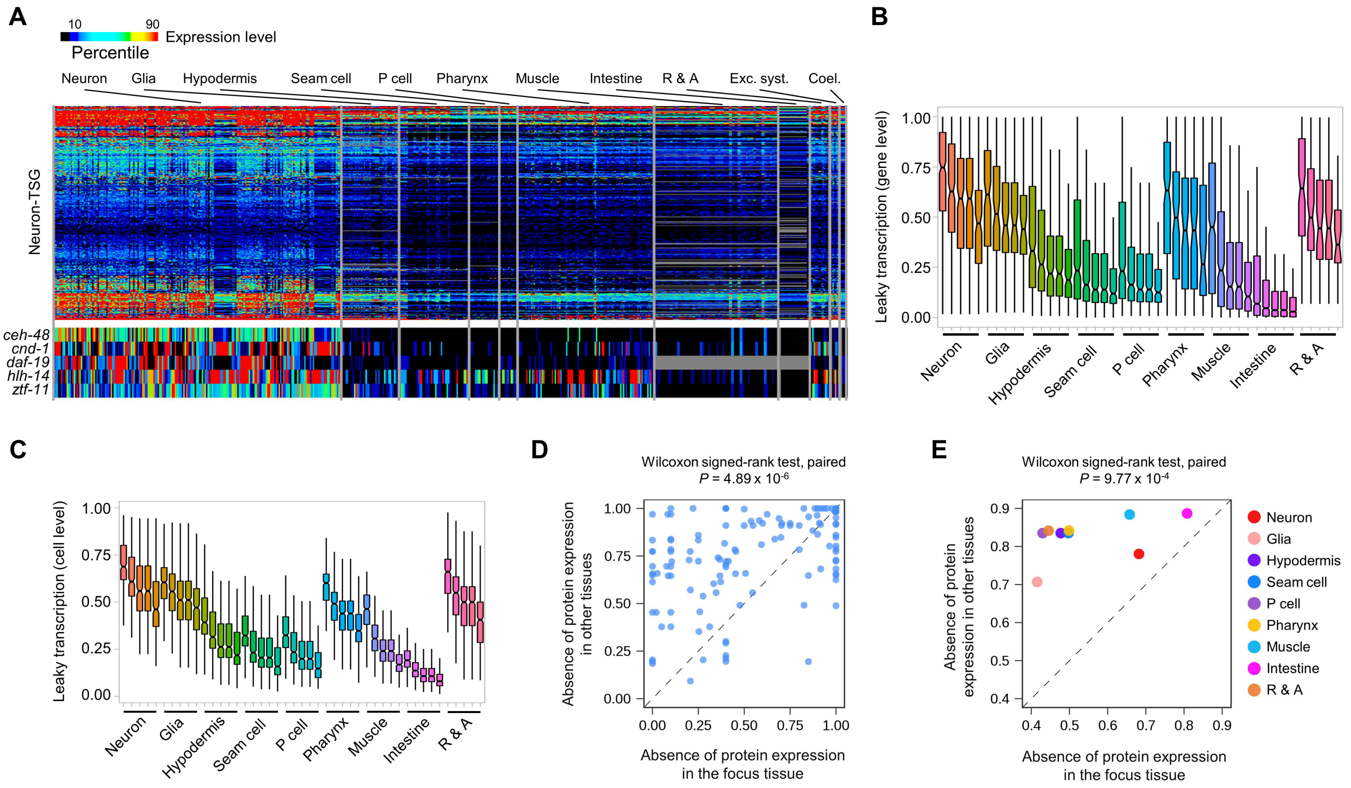

Supplementary Fig. 16. Analysis of leaky transcription.

(A) Expression levels of neuron-TSGs (rows) in individual cell tracks (columns, ordered by cell type). Expression levels of each gene across cell tracks were normalized to percentile rank values for cross-gene comparisons. Below is an enlarged view of the results for five known neuron fate regulators. If a gene is identified as exhibiting specificity in multiple tissues, the expression in non-neuronal cells is removed (dark gray). **(B, C)** Leaky transcription of different types of TSGs identified under different levels of stringency (measured as magnitudes of fold enrichment, 2, 2.5, 3, 3.5, and 4 from left to right). TSGs were identified as having significantly ($Q < 0.01$) great fold change of quantitative expression in cell tracks differentiating into the focus tissue relative to those differentiating into other tissues. Leaky transcription levels for individual genes (B) and cell tracks (C) are provided. **(D)** Comparison of protein expression absence in cells from the focus tissue (X-axis) versus that in cells from other tissue (Y-axis) for each TSG (n = 206). The dashed diagonal indicates equality of X and Y. Protein expression data are from Ma et al. [12]. **(E)** Comparison of protein expression absence in cells from the focus tissue (X-axis) versus that in cells from other tissue (Y-axis) for different types of TSGs. Source data are provided as a Source Data file.

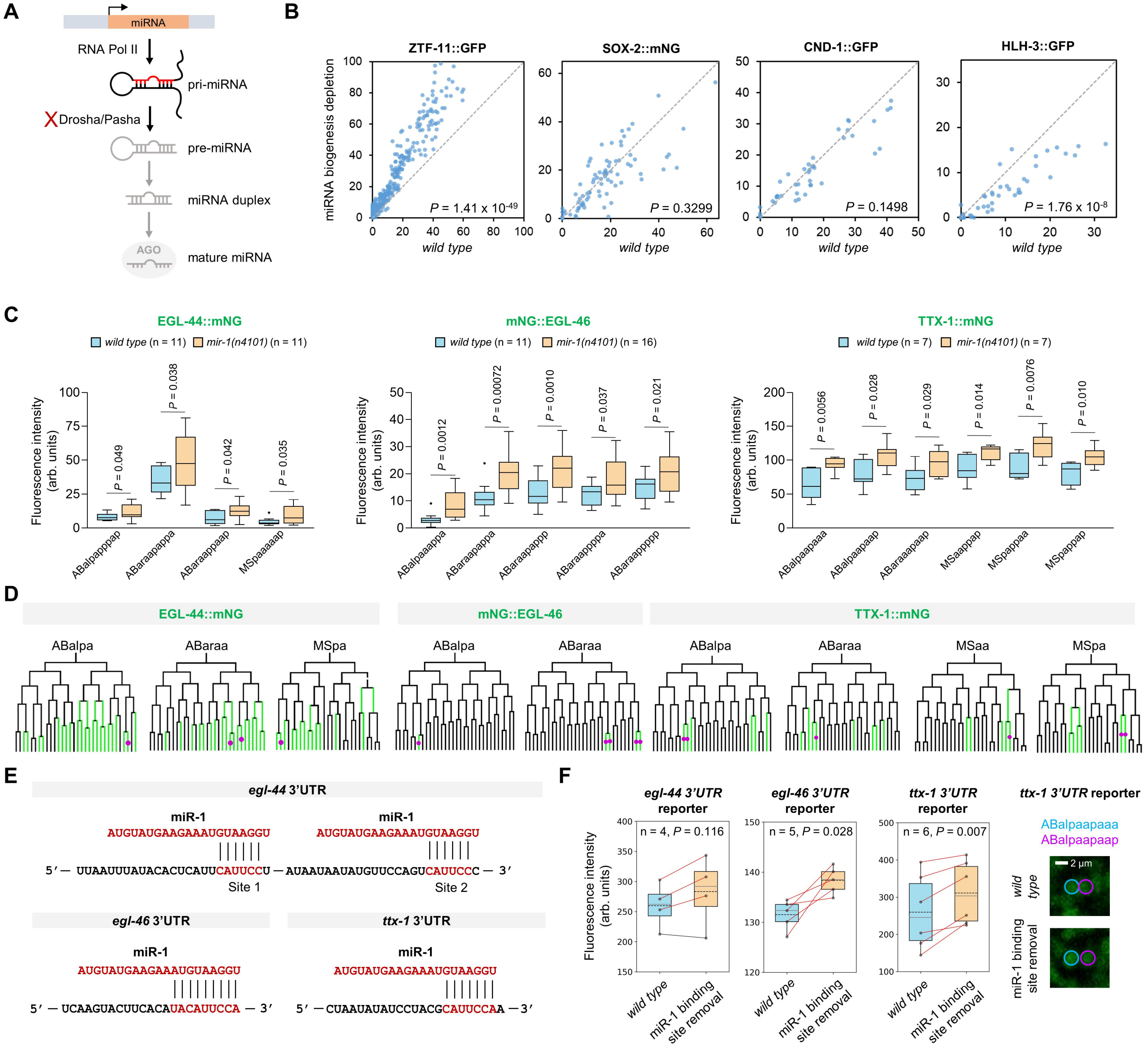

Supplementary Fig. 17. Changes in protein expression following miRNA perturbation.

(A) Schematic representation of depleting miRNA biogenesis by disrupting Drosha and Pasha. **(B)** Scatter plots compare cellular protein expression levels of four neuron-specific transcription factors before (X-axis) and after perturbing miRNA biogenesis. Each dot is a cell (n = 303, 84, 45, and 50 cells from left to right). The dashed diagonal indicates equality of X and Y. Statistics: Wilcoxon signed rank test, two-tailed. **(C)** Comparison of protein expression levels of ELG-44 (left), EGL-46 (middle), and TTX-1(right) between wild-type and *mir-1(n4101)* embryos in cells identified as having expression increase. The data are summarized as boxplots with boxes indicating the inter-quartile range (IQR), whiskers showing the range of values within 1.5*IQR, and horizontal lines indicating medians. Statistics: paired t-test, two-tailed. **(D)** Elevated expression of EGL-44::mNG, mNG::EGL-46, and TTX-1::mNG in *mir-1(n4101)* embryos. In each figure, the tree shows the expression status of corresponding proteins as

determined using endogenous protein fusion reporters; purple dots denote cells for which protein expression was significantly increased in *mir-1(n4101)* embryos. For each TF, we only considered the non-neuronal cell tracks that (1) expressed both miR-1 and the TF and (2) miR-1-pr expression was not later than that of the TF. In addition, to ensure the identification of robust expression increases, we only considered the cells in which the averaged expression level in mutant embryos was moderate (>5). **(E)** Presence of miR-1 binding sites in the 3'UTRs of *elg-44*, *egl-46*, and *ttx-1*. Vertical lines indicate complementarity of the miR-1 seed sequence to the binding site. **(F)** Comparison of fluorescence intensity of corresponding 3'UTR reporters before and after removing the miR-1 binding site. Only cells exhibiting an increase in corresponding protein expression in *mir-1(n4101)* were included for analysis. Micrographs on the right show changes in fluorescence signals in representative cells. The data are summarized as boxplots with boxes indicating the inter-quartile range (IQR), whiskers showing the range of values within 1.5*IQR, and solid and dashed horizontal lines indicating medians and means, respectively. Statistics: paired t-test, two-tailed. Source data are provided as a Source Data file.

Supplementary References

1. Alberti, C. *et al.* Cell-type specific sequencing of microRNAs from complex animal tissues. *Nat Methods* **15**, 283-289 (2018).
2. Zhao, Z. *et al.* A negative regulatory loop between microRNA and Hox gene controls posterior identities in Caenorhabditis elegans. *PLoS Genet* **6**, e1001089 (2010).
3. Pedersen, M.E. *et al.* An epidermal microRNA regulates neuronal migration through control of the cellular glycosylation state. *Science* **341**, 1404-1408 (2013).
4. McCulloch, K.A. & Rougvie, A.E. Caenorhabditis elegans period homolog lin-42 regulates the timing of heterochronic miRNA expression. *Proc Natl Acad Sci U S A* **111**, 15450-15455 (2014).
5. Martinez, N.J. *et al.* Genome-scale spatiotemporal analysis of Caenorhabditis elegans microRNA promoter activity. *Genome Res* **18**, 2005-2015 (2008).
6. Kasuga, H., Fukuyama, M., Kitazawa, A., Kontani, K. & Katada, T. The microRNA miR-235 couples blast-cell quiescence to the nutritional state. *Nature* **497**, 503-506 (2013).
7. Isik, M., Korswagen, H.C. & Berezikov, E. Expression patterns of intronic microRNAs in Caenorhabditis elegans. *Silence* **1**, 5 (2010).
8. Dzakah, E.E. *et al.* Loss of miR-83 extends lifespan and affects target gene expression in an age-dependent manner in Caenorhabditis elegans. *J Genet Genomics* **45**, 651-662 (2018).
9. Clark, A.M. *et al.* The microRNA miR-124 controls gene expression in the sensory nervous system of Caenorhabditis elegans. *Nucleic acids research* **38**, 3780-3793 (2010).
10. Andachi, Y. & Kohara, Y. A whole-mount in situ hybridization method for microRNA detection in Caenorhabditis elegans. *RNA* **22**, 1099-1106 (2016).
11. Ge, S.X., Jung, D. & Yao, R. ShinyGO: a graphical gene-set enrichment tool for animals and plants. *Bioinformatics* **36**, 2628-2629 (2020).
12. Ma, X. *et al.* A 4D single-cell protein atlas of transcription factors delineates spatiotemporal patterning during embryogenesis. *Nat Methods* **18**, 893-902 (2021).
