# Supplementary material for: A lineage-resolved cartography of microRNA promoter activity in C. elegans empowers multidimensional developmental analysis
Source: Nat Commun. 2024 Mar 30;15:2783. doi: 10.1038/s41467-024-47055-4 (PMC10981687; doi:10.1038/s41467-024-47055-4)
Supplement: Supplementary file 3 — Description of Additional Supplementary Information [file 41467_2024_47055_MOESM3_ESM.pdf]

Description of Additional Supplementary Information

File Name: Supplementary Data 1.
Description: *C. elegans* strains and oligonucleotides.

File Name: Supplementary Data 2.
Description: miRNA promoter-driven reporters.

File Name: Supplementary Data 3.
Description: Cellular expression of miRNAs.

File Name: Supplementary Data 4.
Description: Validating cell-specific expression of miRNAs with orthogonal cell-specific markers.

File Name: Supplementary Data 5.
Description: Cell clusters revealed by miRNA expression.

File Name: Supplementary Data 6.
Description: Tissue-specific miRNAs.

File Name: Supplementary Data 7.
Description: Comparison of scCAMERA to the literature.

File Name: Supplementary Data 8.
Description: Previous detection status of tissue enrichment patterns described in scCAMERA.

File Name: Supplementary Data 9.
Description: Tissue-specific TFs.

File Name: Supplementary Data 10.
Description: RNA-seq analysis of the *mir-1* mutant.

File Name: Supplementary Data 11.
Description: Influence of miR-1 on cell lineage and cell position in pharyngeal progenitors.

File Name: Supplementary Data 12.
Description: Preferential binding of TFs at miRNA promoters.

File Name: Supplementary Data 13.
Description: Influence of fate determinants on the expression of tissue-specific miRNAs.

File Name: Supplementary Data 14.
Description: Influence of fate determinant binding on the expression of tissue-specific miRNAs.

File Name: Supplementary Data 15.
Description: Developmental properties of miRNA targets.

File Name: Supplementary Data 16.
Description: ZTF-11, SOX-2, CND-1 protein expression before and after depleting miRNA biogenesis.

File Name: Supplementary Data 17.
Description: Protein expression levels of EGL-44, EGL-46, and TTX-1 in wild-type and *mir-1(n4101)* embryos.

File Name: Supplementary Data 18.
Description: mNG::H2B expression levels of *egl-44*, *egl-46*, and *ttx-1* 3'UTR fluorescent reporters before and after the removal of miR-1 binding site.
