# Supplementary material for: A lineage-resolved cartography of microRNA promoter activity in C. elegans empowers multidimensional developmental analysis
Source: Nat Commun. 2024 Mar 30;15:2783. doi: 10.1038/s41467-024-47055-4 (PMC10981687; doi:10.1038/s41467-024-47055-4)
Supplement: Supplementary file 7 — Supplementary Dataset 4 [file 41467_2024_47055_MOESM7_ESM.pdf]

Validating the identity of cells expressing miR-83-pr in ASEL/R cells using the *che-1* reporter (embryonic expression)

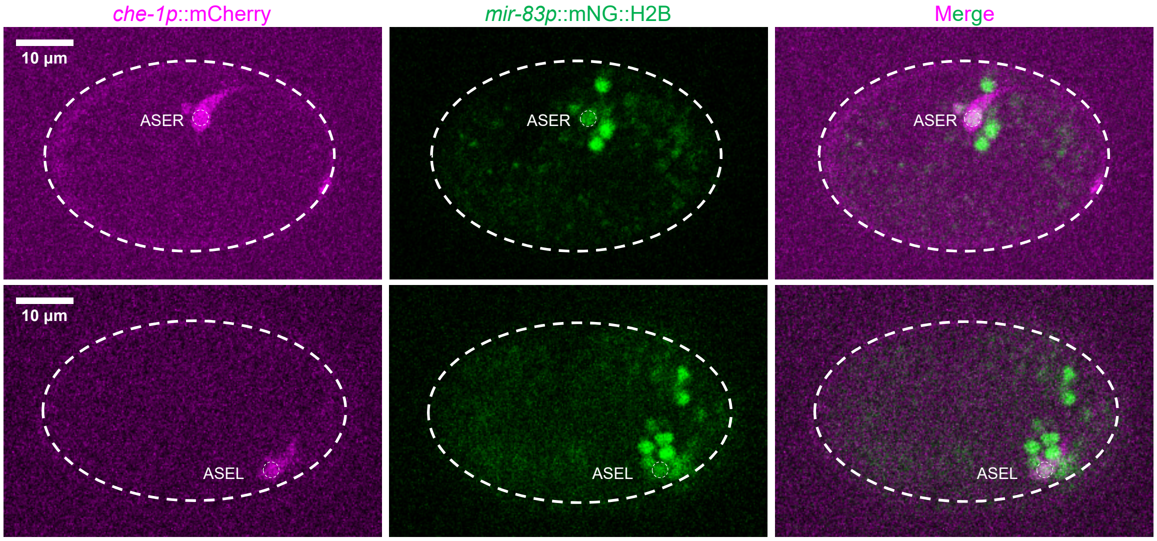

Lineage name	Functional name	Marker (*che-1p*)	Presence (+) or absence (-) of fluorescence signal of miRNA-pr in the cell	Cellular expression level of miR-83-pr in scST-miRNA atlas
ABalppppppaa	ASEL	on	+	33.07
ABpraaapppaa	ASER	on	+	9.85

Validating the identity of cells expressing miR-83-pr in ASEL/R cells using the *che-1* reporter (L1 stage expression)

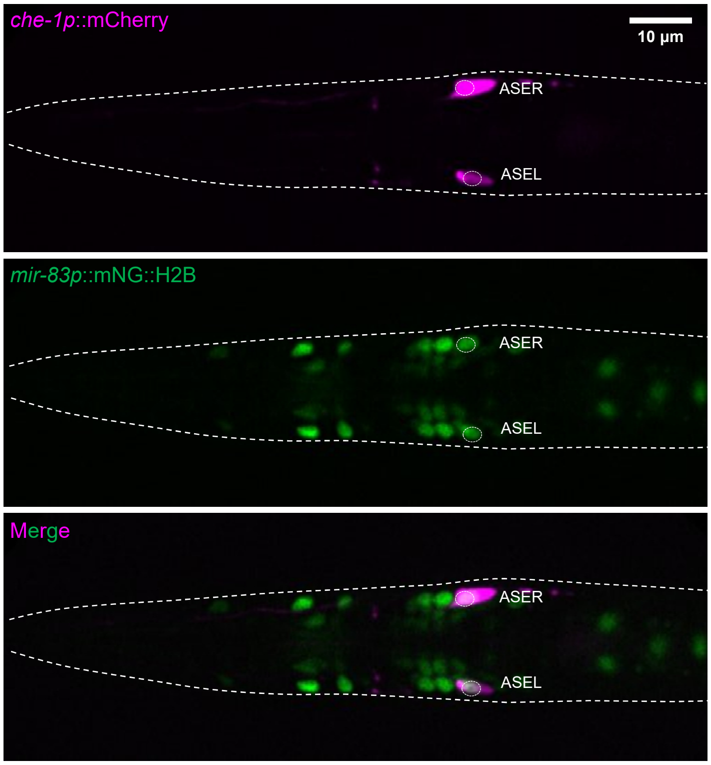

Lineage name	Functional name	Marker (*che-1p*)	Presence (+) or absence (-) of fluorescence signal of miRNA-pr in the cell	Cellular expression level of miR-83-pr in scST-miRNA atlas
ABalpppppppaa	ASEL	on	+	5.15
ABpraaapppaa	ASER	on	+	5.31

Validating the identity of cells expressing miR-124-pr in ASEL/R cells using the *che-1* reporter (embryonic expression)

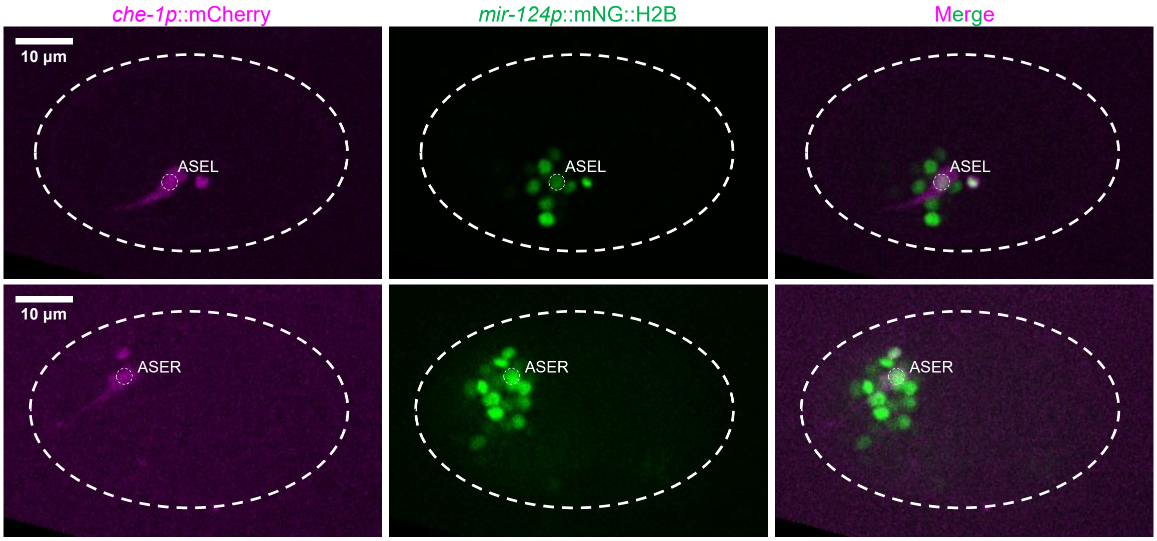

Lineage name	Functional name	Marker (*che-1p*)	Presence (+) or absence (-) of fluorescence signal of miRNA-pr in the cell	Cellular expression level of miR-124-pr in scST-miRNA atlas
ABalpppppaa	ASEL	on	+	272.83
ABpraaapppaa	ASER	on	+	390.76

Validating the identity of cells expressing miR-124-pr in ASEL/R cells using the *che-1* reporter (L1 stage expression)

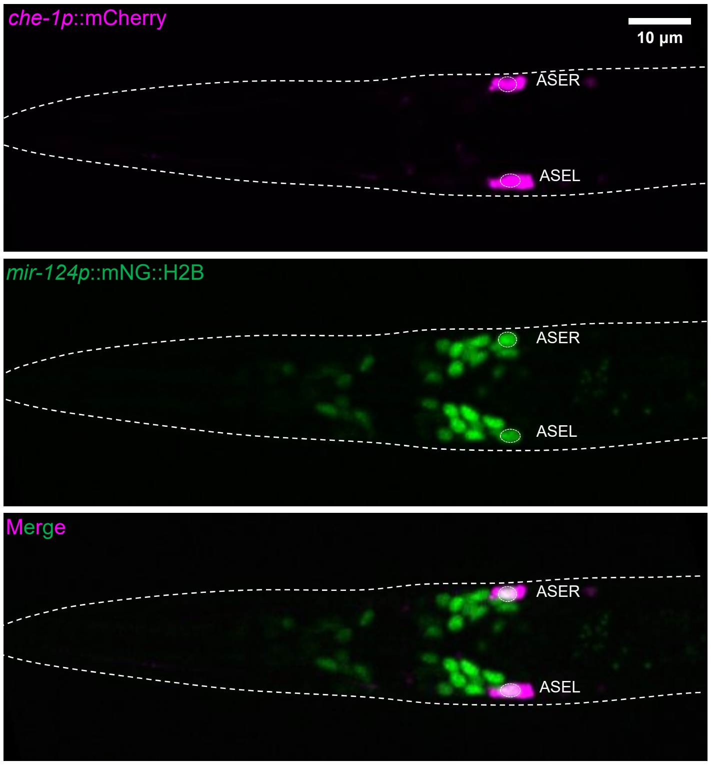

Lineage name	Functional name	Marker (*che-1p*)	Presence (+) or absence (-) of fluorescence signal of miRNA-pr in the cell	Cellular expression level of miR-124-pr in scST-miRNA atlas
ABalpppppppaa	ASEL	on	+	5.96
ABpraaapppaa	ASER	on	+	6.24

Validating the identity of cells expressing miR-232-pr in ASEL/R cells using the *che-1* reporter (L1 stage expression)

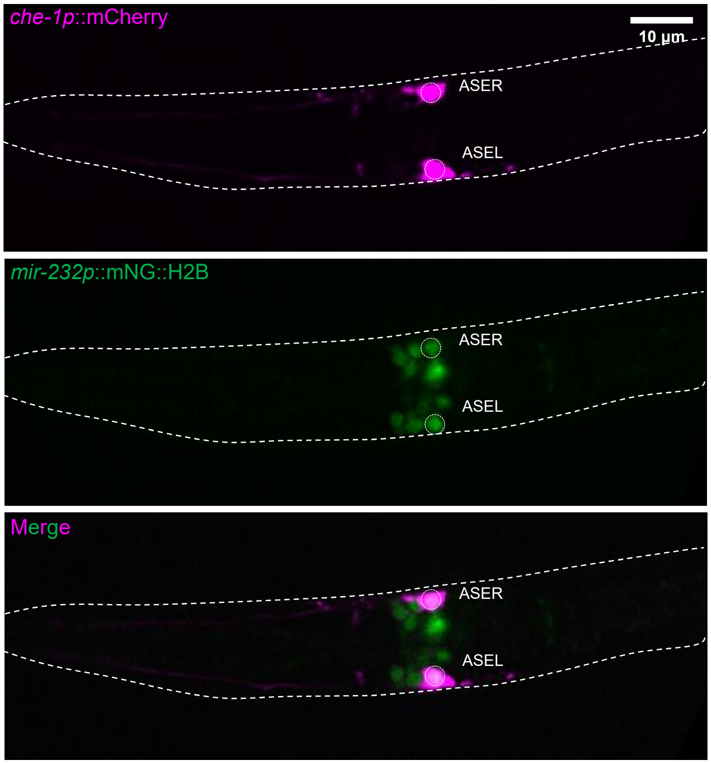

Lineage name	Functional name	Marker (*che-1p*)	Presence (+) or absence (-) of fluorescence signal of miRNA-pr in the cell	Cellular expression level of miR-232-pr in scST-miRNA atlas
ABalpppppaa	ASEL	on	+	1.26
ABpraaapppaa	ASER	on	+	2.96

Validating the identity of cells expressing miR-2-pr in AIYL/R cells using the *ttx-3* reporter (L1 stage expression)

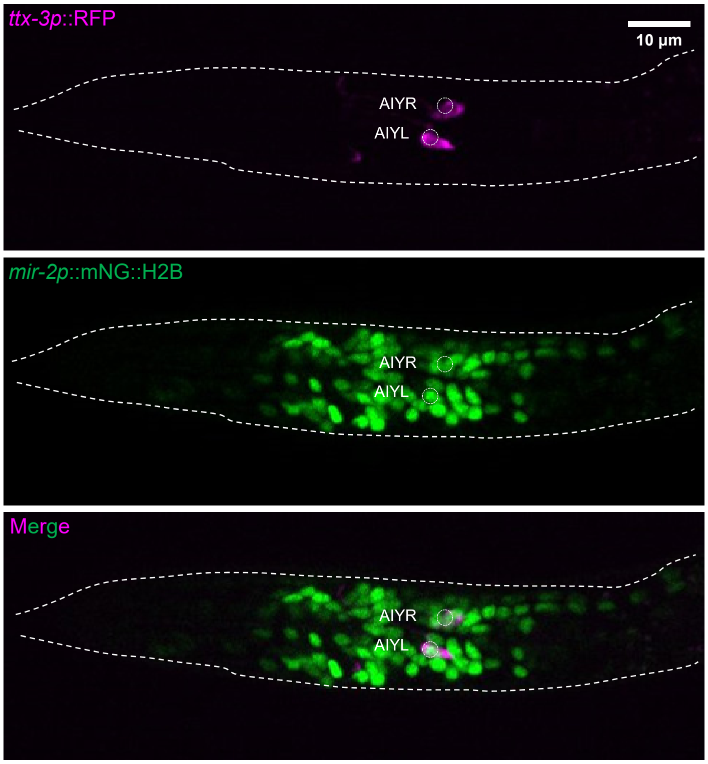

Lineage name	Functional name	Marker (*ttx-3p*)	Presence (+) or absence (-) of fluorescence signal of miRNA-pr in the cell	Cellular expression level of miR-2-pr in scST-miRNA atlas
ABplpapaaap	AIYL	on	+	6.43
ABprpapaaap	AIYR	on	+	6.21

Validating the identity of cells expressing miR-794-pr in AIYL/R cells using the *ttx-3* reporter (L1 stage expression)

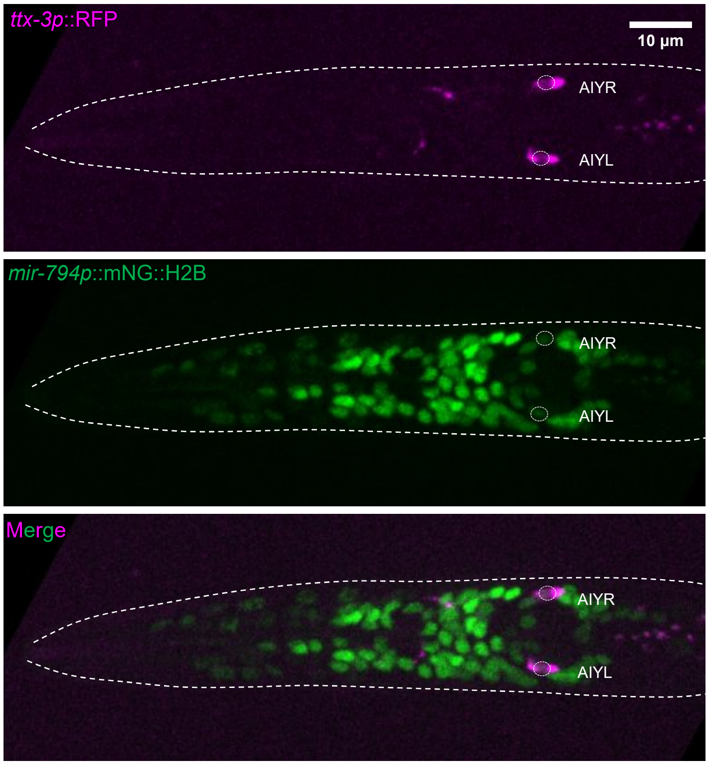

Lineage name	Functional name	Marker (*ttx-3p*)	Presence (+) or absence (-) of fluorescence signal of miRNA-pr in the cell	Cellular expression level of miR-794-pr in scST-miRNA atlas
ABplpapaaap	AIYL	on	+	3.69
ABprpapaaap	AIYR	on	+	2.59

Validating the identity of cells expressing miR-2-pr in NSML/R cells using the *mgl-1* reporter (L1 stage expression)

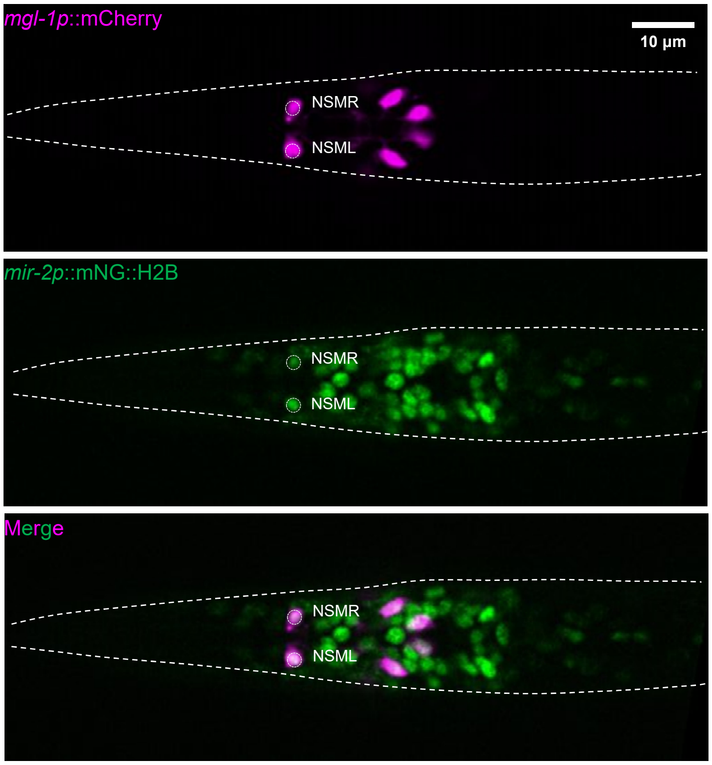

Lineage name	Functional name	Marker (*mgl-1p*)	Presence (+) or absence (-) of fluorescence signal of miRNA-pr in the cell	Cellular expression level of miR-2-pr in scST-miRNA atlas
ABaraapapaav	NSML	on	+	5.2
ABaraapppaav	NSMR	on	+	5.37

Validating the identity of cells expressing miR-124-pr in NSML/R cells using the *mgl-1* reporter (L1 stage expression)

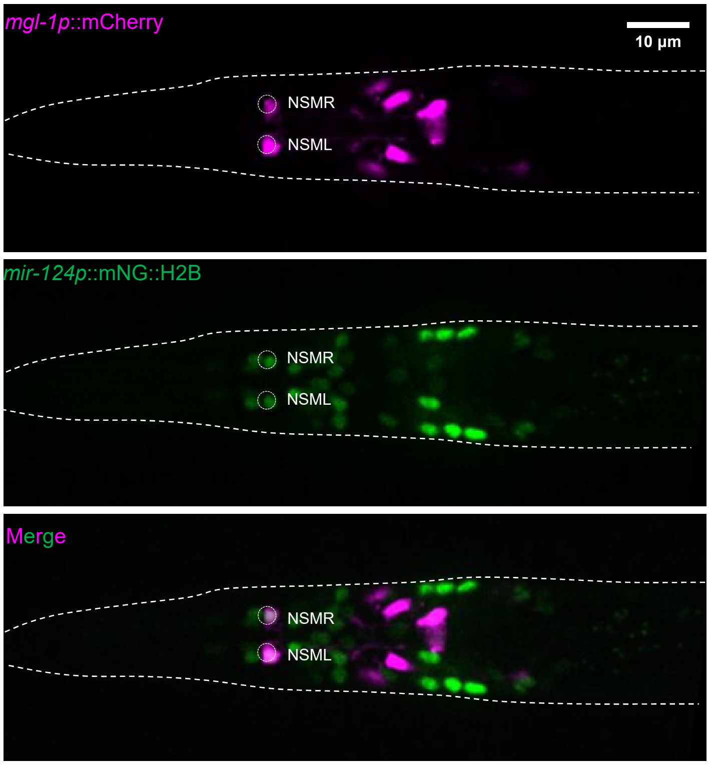

Lineage name	Functional name	Marker (*mgl-1p*)	Presence (+) or absence (-) of fluorescence signal of miRNA-pr in the cell	Cellular expression level of miR-124-pr in scST-miRNA atlas
ABaraapapaav	NSML	on	+	3.61
ABaraapppaav	NSMR	on	+	3.9

Validating the identity of cells expressing miR-794-pr in NSML/R cells using the *mgl-1* reporter (L1 stage expression)

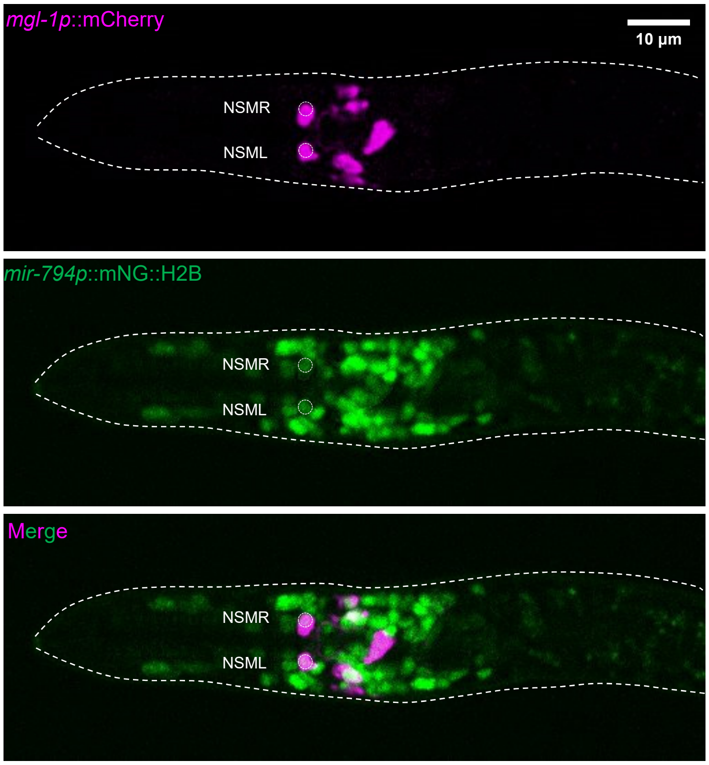

Lineage name	Functional name	Marker (*mgl-1p*)	Presence (+) or absence (-) of fluorescence signal of miRNA-pr in the cell	Cellular expression level of miR-794-pr in scST-miRNA atlas
ABaraapapaav	NSML	on	+	4.08
ABaraapppaav	NSMR	on	+	4.21

Validating the identity of cells expressing let-7-pr in coelomocytes using the *unc-122* reporter (L1 stage expression)

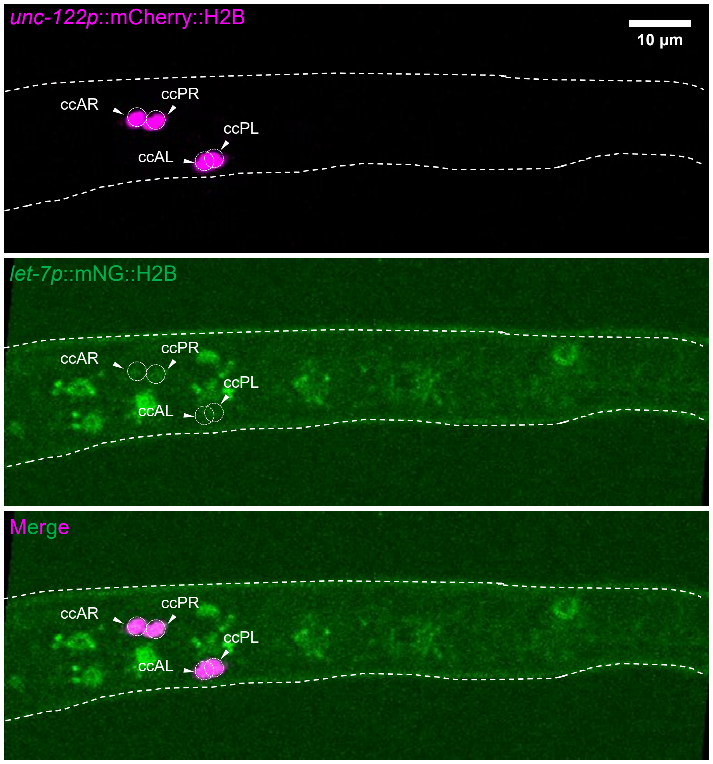

Lineage name	Functional name	Marker (*unc-122p*)	Presence (+) or absence (-) of fluorescence signal of miRNA-pr in the cell	Cellular expression level of let-7-pr in scST-miRNA atlas
MSppapaaa	ccAR	on	+	3.06
MSppapaap	ccPR	on	+	3.27
MSapapaaa	ccAL	on	+	3.68
MSapapaap	ccPL	on	+	3.44

Validating the identity of cells expressing miR-245-pr in coelomocytes using the *unc-122* reporter (L1 stage expression)

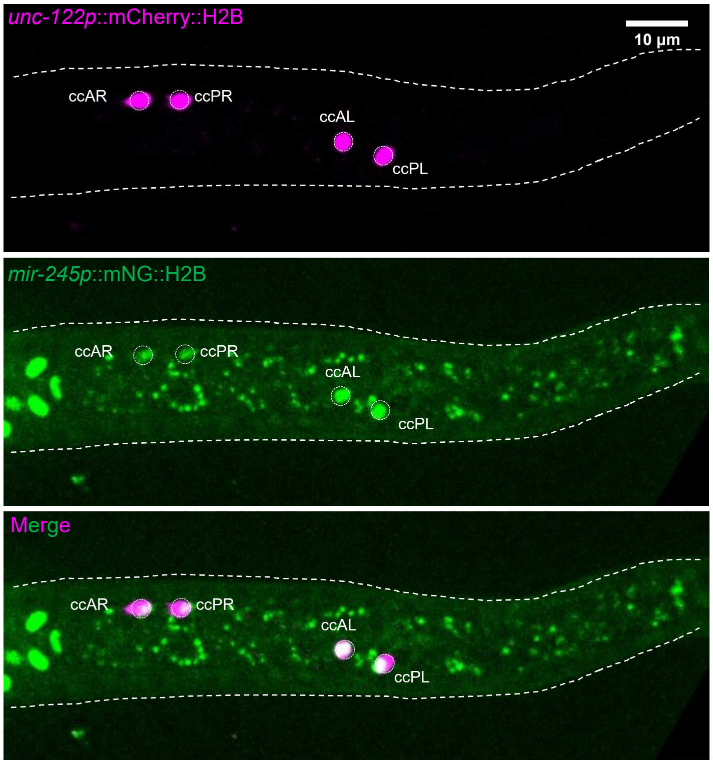

Lineage name	Functional name	Marker (*unc-122p*)	Presence (+) or absence (-) of fluorescence signal of miRNA-pr in the cell	Cellular expression level of miR-245-pr in scST-miRNA atlas
MSppapaaa	ccAR	on	+	2.81
MSppapaap	ccPR	on	+	2.01
MSapapaaa	ccAL	on	+	0.56
MSapapaap	ccPL	on	+	0.52

Validating the identity of cells expressing miR-45-pr in intestine cells using the *elt-2* reporter (embryonic expression)

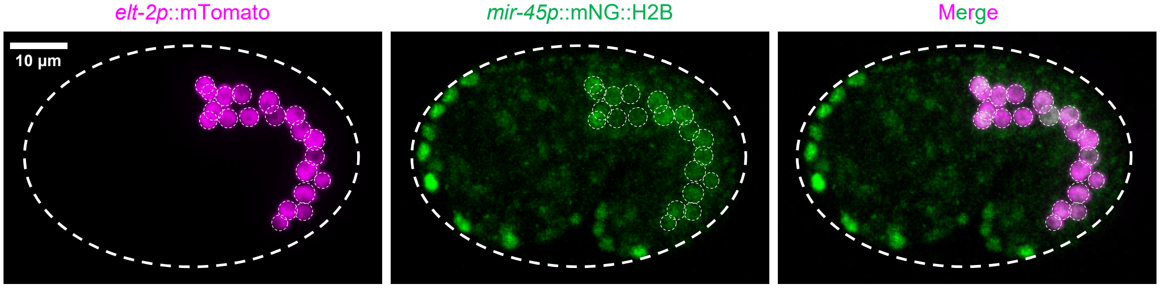

Lineage name	Functional name	Marker (*elt-2p*)	Presence (+) or absence (-) of fluorescence signal of miRNA-pr in the cell	Cellular expression level of miR-45-pr in scST-miRNA atlas
Ealaad	int1DL	on	+	25.5
Ealaav	int1VL	on	+	29.2
Ealap	int3V	on	+	19
Ealpa	int2V	on	+	19.9
Ealpp	int5L	on	+	14.2
Earaad	int1DR	on	+	22.8
Earaav	int1VR	on	+	21.6
Earap	int3D	on	+	14.1
Earpa	int2D	on	+	11.5
Earpp	int5R	on	+	8.66
Eplaa	int4V	on	+	15.6
Eplap	int6L	on	+	16.2
Eplpa	int7L	on	+	16.7
Eplppa	int8L	on	+	30
Eplppp	int9L	on	+	28.6
Epraa	int4D	on	+	7.43
Eprap	int6R	on	+	13.3
Eprpa	int7R	on	+	6.66
Eprppa	int8R	on	+	18.4
Eprppp	int9R	on	+	26.5

Validating the identity of cells expressing miR-49-pr in intestine cells using the *elt-2* reporter (embryonic expression)

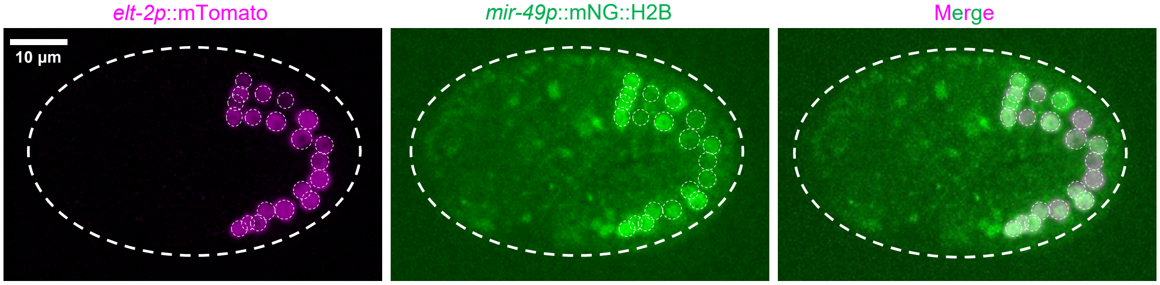

Lineage name	Functional name	Marker (*elt-2p*)	Presence (+) or absence (-) of fluorescence signal of miRNA-pr in the cell	Cellular expression level of miR-49-pr in scST-miRNA atlas
Ealaad	int1DL	on	+	28.9
Ealaav	int1VL	on	+	51.8
Ealap	int3V	on	+	23
Ealpa	int2V	on	+	27.9
Ealpp	int5L	on	+	25.3
Earaad	int1DR	on	+	47.8
Earaav	int1VR	on	+	19.2
Earap	int3D	on	+	30.8
Earpa	int2D	on	+	29.1
Earpp	int5R	on	+	29.3
Eplaa	int4V	on	+	22.6
Eplap	int6L	on	+	28.2
Eplpa	int7L	on	+	29.8
Eplppa	int8L	on	+	64.4
Eplppp	int9L	on	+	43.7
Epraa	int4D	on	+	16.8
Eprap	int6R	on	+	28.2
Eprpa	int7R	on	+	23.8
Eprppa	int8R	on	+	56.6
Eprppp	int9R	on	+	53

Validating the identity of cells expressing miR-51-pr in hypodermal cells using the *pax-3* reporter (embryonic expression)

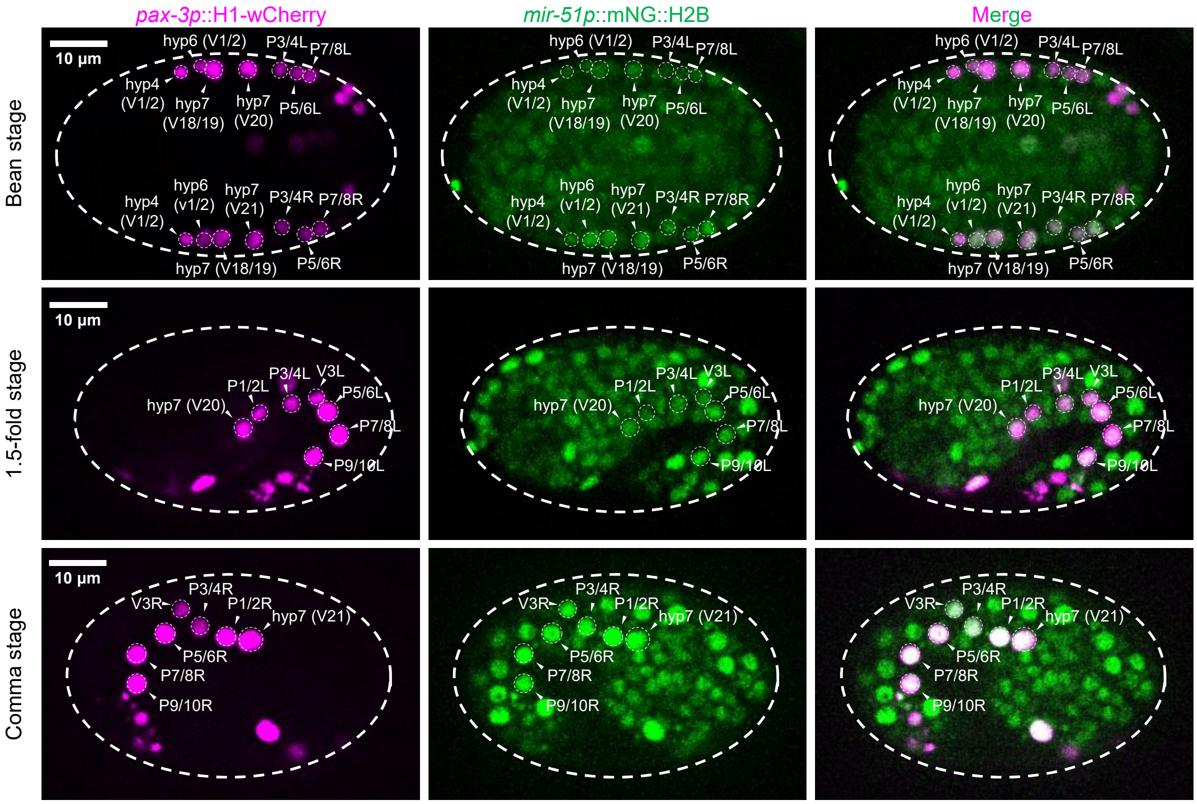

Lineage name	Functional name	Marker (*pax-3p*)	Presence (+) or absence (-) of fluorescence signal of miRNA-pr in the cell	Cellular expression level of miR-51-pr in scST-miRNA atlas
ABplaappaa	hyp4 (V1/2)	on	+	7.26
ABplaappap	hyp6 (V1/2)	on	+	23.7
ABplaapppa	hyp7 (V18/19)	on	+	38.35
ABplaapppp	hyp7 (V20)	on	+	39.44
ABplapaapp	P1/2L	on	+	38.25
ABplapapap	P9/10L	on	+	32.74
ABplappaaa	P3/4L	on	+	51.16
ABplappaap	P5/6L	on	+	46.36
ABplappapa	V3L	on	+	36.46
ABplappapp	P7/8L	on	+	29.85
ABpraappaa	hyp4 (V1/2)	on	+	16.66
ABpraappap	hyp6 (V1/2)	on	+	26.11
ABpraapppa	hyp7 (V18/19)	on	+	36.66
ABpraapppp	hyp7 (V21)	on	+	42.72
ABprapaapp	P1/2R	on	+	28.99
ABprapapap	P9/10R	on	+	34.92
ABprappaaa	P3/4R	on	+	80.1
ABprappaap	P5/6R	on	+	47.08
ABprappapa	V3R	on	+	20.32
ABprappapp	P7/8R	on	+	14.76

Validating the identity of cells expressing miR-79-pr in hypodermal cells using the *pax-3* reporter (embryonic expression)

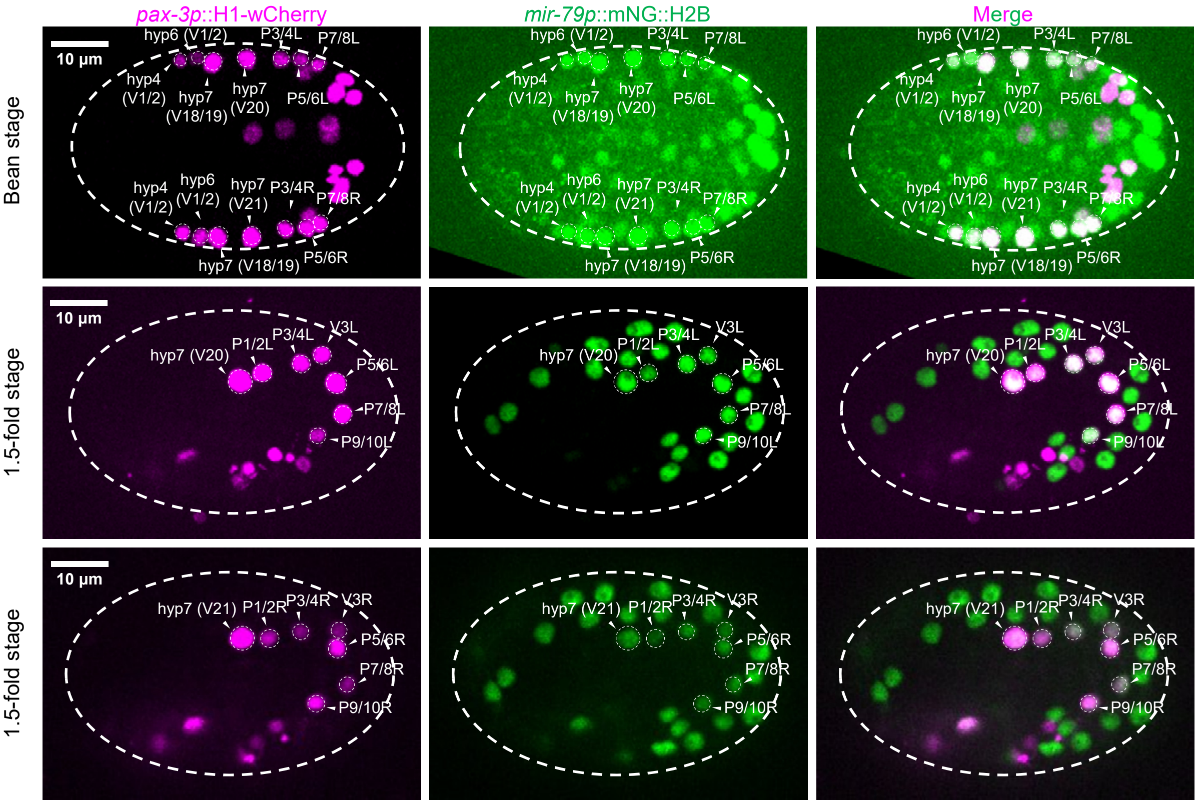

Lineage name	Functional name	Marker (*pax-3p*)	Presence (+) or absence (-) of fluorescence signal of miRNA-pr in the cell	Cellular expression level of miR-79-pr in scST-miRNA atlas
ABplaappaa	hyp4 (V1/2)	on	+	55.74
ABplaappap	hyp6 (V1/2)	on	+	66.39
ABplaapppa	hyp7 (V18/19)	on	+	66.48
ABplaapppp	hyp7 (V20)	on	+	105.6
ABplapaapp	P1/2L	on	+	16.23
ABplapapap	P9/10L	on	+	56.72
ABplappaaa	P3/4L	on	+	78.55
ABplappaap	P5/6L	on	+	77.47
ABplappapa	V3L	on	+	71.7
ABplappapp	P7/8L	on	+	49.22
ABpraappaa	hyp4 (V1/2)	on	+	54.8
ABpraappap	hyp6 (V1/2)	on	+	59.27
ABpraapppa	hyp7 (V18/19)	on	+	87.06
ABpraapppp	hyp7 (V21)	on	+	128
ABprapaapp	P1/2R	on	+	28.16
ABprapapap	P9/10R	on	+	110.5
ABprappaaa	P3/4R	on	+	104.6
ABprappaap	P5/6R	on	+	114.5
ABprappapa	V3R	on	+	45.83
ABprappapp	P7/8R	on	+	97.45

Validating the identity of cells expressing miR-231-pr in hypodermal cells using the *pax-3* reporter (embryonic expression)

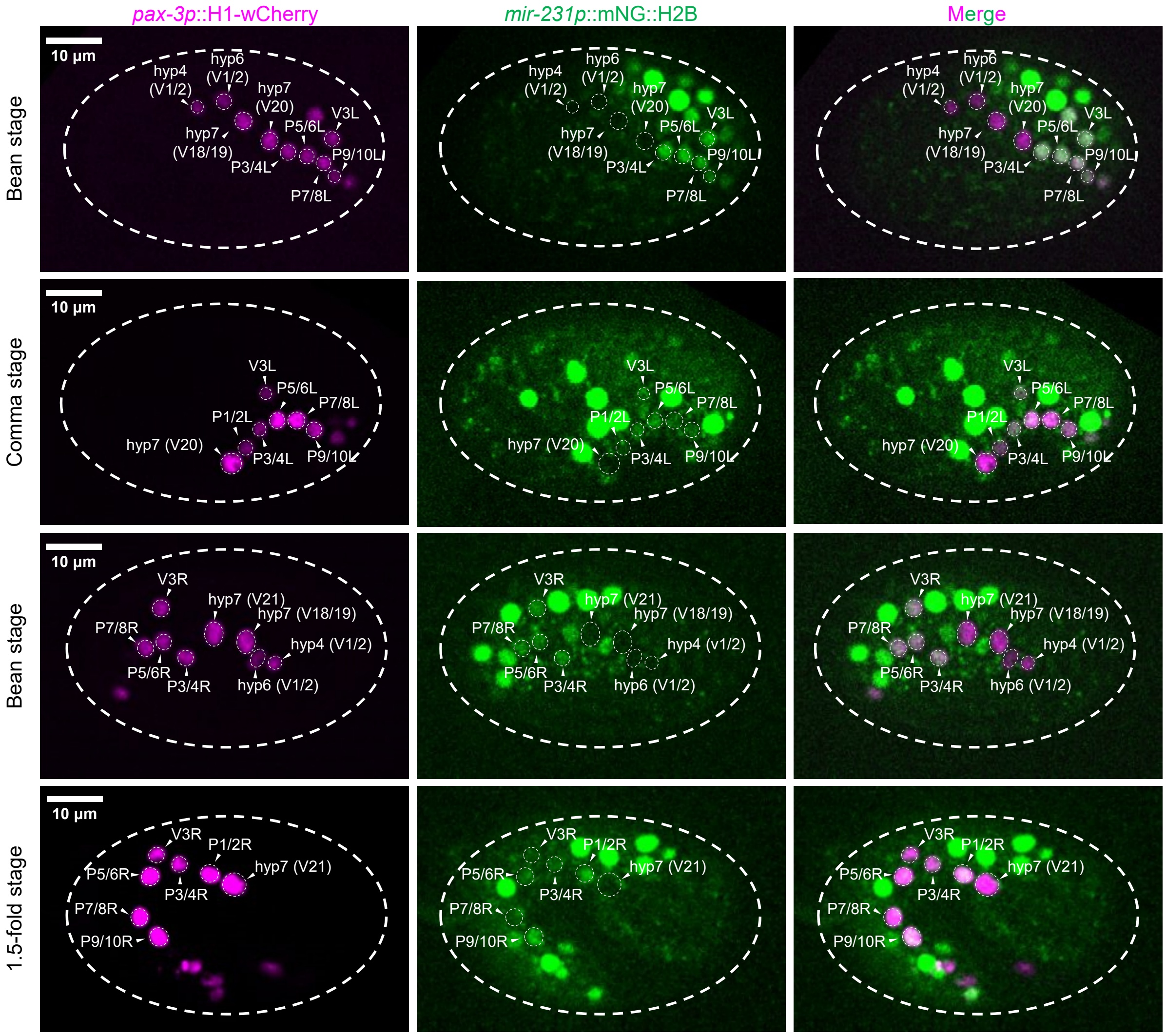

Lineage name	Functional name	Marker (*pax-3p*)	Presence (+) or absence (-) of fluorescence signal of miRNA-pr in the cell	Cellular expression level of miR-231-pr in scST-miRNA atlas
ABplaappaa	hyp4 (V1/2)	on	-	0
ABplaappap	hyp6 (V1/2)	on	-	0
ABplaapppa	hyp7 (V18/19)	on	-	0
ABplaapppp	hyp7 (V20)	on	-	0
ABplapaapp	P1/2L	on	+	21.42
ABplapapap	P9/10L	on	+	47.79
ABplappaaa	P3/4L	on	+	22.04
ABplappaap	P5/6L	on	+	20.81
ABplappapa	V3L	on	+	16.46
ABplappapp	P7/8L	on	+	23.39
ABpraappaa	hyp4 (V1/2)	on	-	0
ABpraappap	hyp6 (V1/2)	on	-	0
ABpraapppa	hyp7 (V18/19)	on	-	0
ABpraapppp	hyp7 (V21)	on	-	0
ABprapaapp	P1/2R	on	+	9.12
ABprapapap	P9/10R	on	+	13.49
ABprappaaa	P3/4R	on	+	6.65
ABprappaap	P5/6R	on	+	6.6
ABprappapa	V3R	on	+	8.56
ABprappapp	P7/8R	on	+	5.14

Validating the identity of cells expressing miR-43-44-pr in neurons using the *hlh-16* reporter (embryonic expression)

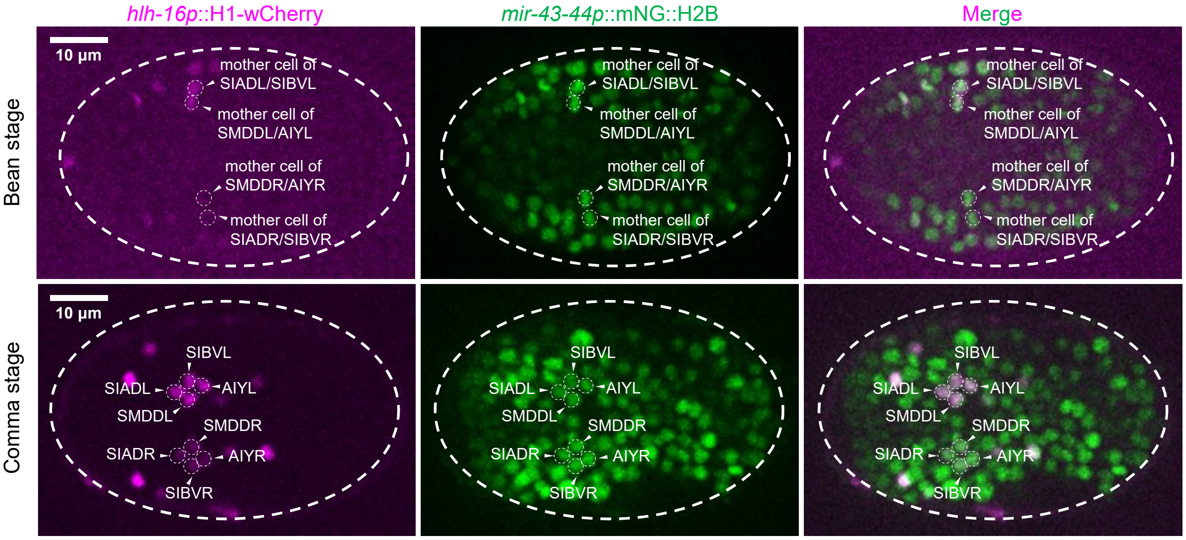

Lineage name	Functional name	Marker (*hlh-16p*)	Presence (+) or absence (-) of fluorescence signal of miRNA-pr in the cell	Cellular expression level of miR-43-44-pr in scST-miRNA atlas
ABplpapaaa	Mother of AIYL and SMDDL	on	+	18.95
ABplpapaaap	AIYL	on	+	14.84
ABplpapaaaa	SMDDL	on	+	16.16
ABplpapaap	Mother of SIADL and SIBVL	on	+	16.5
ABplpapaapa	SIADL	on	+	17.95
ABplpapaapp	SIBVL	on	+	19.32
ABprpapaaa	Mother of AIYR and SMDDR	on	+	16.36
ABprpapaaap	AIYR	on	+	12.83
ABprpapaaaa	SMDDR	on	+	14.09
ABprpapaap	Mother of SIADR and SIBVR	on	+	15.89
ABprpapaapa	SIADR	on	+	14.56
ABprpapaapp	SIBVR	on	+	14.75
