# Supplementary material for: A lineage-resolved cartography of microRNA promoter activity in C. elegans empowers multidimensional developmental analysis
Source: Nat Commun. 2024 Mar 30;15:2783. doi: 10.1038/s41467-024-47055-4 (PMC10981687; doi:10.1038/s41467-024-47055-4)
Supplement: Supplementary file 10 — Supplementary Dataset 7 [file 41467_2024_47055_MOESM10_ESM.pdf]

#	miRNA	**Expression described in the literature** (Previous studies are numbered and the method used to determine the expression is indicated)	**Summary of expression in the literature** (Descriptions consistent with this study are cyan-shaded; partially consistent with this study are yellow-shaded)	**Expression in this study**	**Comparison results**
1	let-7	1. Reporter: expression detected from late embryos to adults. In late embryos to L1, expression is seen in dnc and intestine [1].<br>2. Reporter: For example, gut and pharyngeal expression began in the L1 [2].	1. Reporter [1]:<br>Tissue level: intestine<br>Cell level: dorsal nerve cord<br>2. Reporter [2]:<br>Tissue level: intestine; pharynx	**Enriched tissue:**<br>hypodermis (sub)<br>intestine (pan)<br>coelomocyte (pan)<br><br>**Expression status:**<br>neuron (4.0%)<br>glia (15.0%)<br>hypodermis (16.7%)<br>seam cell (0.0%)<br>P cell (0.0%)<br>pharynx (20.0%)<br>muscle (13.8%)<br>intestine (90.0%)<br>rectum & anus (40.0%)<br>excretory system (16.7%)<br>coelomocyte (100.0%)	1. Partially consistent<br>2. Partially consistent<br><br>Conclusion: partially consistent
2	lin-4	1. Sequencing: in neurons, intestine, pharynx and muscle (L1 stage, mime-seq, supplemental) [3].<br>2. Reporter: expression seen from late L1 to adult stages. Weak expression detected	1. Sequencing [3]:<br>Tissue level: neuron; intestine; pharynx; muscle<br>2. Reporter [1]:<br>Tissue level: pharynx; body wall muscle	**Enriched tissue:**<br>pharynx(sub)<br><br>**Expression status:**<br>neuron (0.0%)	1. Partially consistent<br>2. Partially consistent

		ubiquitously (except germline). Stronger in pharynx, vulva, vulval muscle, body wall muscle [1].		glia (0.0%)<br>hypodermis (0.0%)<br>seam cell (0.0%)<br>P cell (0.0%)<br>pharynx (3.2%)<br>muscle (0.0%)<br>intestine (0.0%)<br>rectum & anus (0.0%)<br>excretory system (0.0%)<br>coelomocyte (0.0%)	Conclusion:<br>partially consistent
3	miR-1	1. Sequencing: in muscle and pharynx (L1 stage, mime-seq, supplemental) [3].<br>2. Reporter: expression detected from early embryos to adults specifically in pharynx and precursors [1].<br>3. In situ hybridization: observed in the body-wall muscle at the adult stage. Additionally, *mir-1* miRNA was detected in the anal depressor muscle and the pharynx, which is a muscular tube used to suck food. The miRNA was also detected in sex-specific muscles, i.e., the vulval muscle in hermaphrodites and the diagonal muscle in males. Analyses of larvae by the method also gave signals in these muscles at each of the four larval stages (Fig. 1D–G). Pharyngeal signals were detected in	1. Sequencing [3]:<br>Tissue level: muscle; pharynx<br>2. Reporter [1]:<br>Tissue level: pharynx<br>3. In situ hybridization [4]:<br>Tissue level: body-wall muscle; pharynx<br>Cell level: anal depressor muscle	**Enriched tissue:**<br>pharynx (pan)<br>muscle (pan)<br><br>**Expression status:**<br>neuron (0.0%)<br>glia (0.0%)<br>hypodermis (0.0%)<br>seam cell (0.0%)<br>P cell (0.0%)<br>pharynx (73.7%)<br>muscle (93.1%)<br>intestine (5.0%)<br>rectum & anus (60.0%)<br>excretory system (0.0%)<br>coelomocyte (0.0%)	1. Consistent<br>2. Consistent<br>3. Consistent<br><br>Conclusion:<br>consistent

		embryos at the 500-cell stage and later (Fig. 1H–K). [4].			
4	miR-1018	No reference.	No reference.	**Expression status:**<br>neuron (0.0%)<br>glia (0.0%)<br>hypodermis (0.0%)<br>seam cell (0.0%)<br>P cell (0.0%)<br>pharynx (0.0%)<br>muscle (0.0%)<br>intestine (0.0%)<br>rectum & anus (0.0%)<br>excretory system (0.0%)<br>coelomocyte (0.0%)	
5	miR-1022	1. Sequencing and Reporter: in neurons (L1 stage, mime-seq, supplemental), few sensory neurons & dim in vulva and uterus (fosmid reporter, supplemental) [3].	1. Sequencing and Reporter [3]:<br>Tissue level: neuron	**Enriched tissue:**<br>seam cell (sub)<br>pharynx (sub)<br>rectum & anus (sub)<br><br>**Expression status:**<br>neuron (1.0%)<br>glia (0.0%)<br>hypodermis (0.0%)<br>seam cell (0.0%)<br>P cell (0.0%)<br>pharynx (22.1%)	Conclusion: partially consistent

				muscle (2.3%)<br>intestine (0.0%)<br>rectum & anus (66.7%)<br>excretory system (16.7%)<br>coelomocyte (0.0%)	
6	miR-124	1. Sequencing and Reporter: in neurons (L1 stage, mime-seq, supplemental), Neurons in the head (close to pharynx) and in the tail (fosmid reporter, supplemental) [3].<br>2. Reporter: Nervous system [5].<br>3. Reporter: from mid-embryogenesis (350min post-fertilization), when neuronal differentiation begins, throughout development and in adults. We detected *mir-124* promoter::*gfp* expression in 40 of the 302 neurons in C. elegans (some of these as sensory neurons most of which are ciliated [AWC, AWA, AWB, ASH, ASI, ASK, PVQ (not ciliated), ASE, PHA, PHB, PVD (not ciliated), IL1, ADE, PDE) [6].	1. Sequencing and Reporter [3]:<br>Tissue level: neuron<br>2. Reporter [5]:<br>Tissue level: neuron<br>3. Reporter [6]:<br>Cell level: 40 of the 302 neurons in C. elegans (some of these as sensory neurons most of which are ciliated [AWC, AWA, AWB, ASH, ASI, ASK, PVQ (not ciliated), ASE, PHA, PHB, PVD (not ciliated), IL1, ADE, PDE)	**Enriched tissue:**<br>neuron (sub)<br><br>**Expression status:**<br>neuron (38.6%)<br>glia (0.0%)<br>hypodermis (0.0%)<br>seam cell (0.0%)<br>P cell (0.0%)<br>pharynx (14.7%)<br>muscle (0.0%)<br>intestine (0.0%)<br>rectum & anus (0.0%)<br>excretory system (0.0%)<br>coelomocyte (0.0%)	1. Partially consistent<br>2. Partially consistent<br>3. Partially consistent<br><br>Conclusion:<br>partially consistent
7	miR-2	1. Sequencing: in muscle (L1 stage, mime-seq, supplemental) [3].<br>2. Reporter: expressed from late embryos to adulthood. Strong expression detected in many nerves, nerve ring, dnc, vnc an also nerves in the tail [1].	1. Sequencing [3]:<br>Tissue level: muscle<br>2. Reporter [1]:<br>Tissue level: neuron	**Enriched tissue:**<br>neuron (pan)<br>muscle (pan)<br><br>**Expression status:**<br>neuron (84.2%)	1. Consistent<br>2. Consistent<br><br>Conclusion:<br>consistent

				glia (0.0%)<br>hypodermis (0.0%)<br>seam cell (0.0%)<br>P cell (0.0%)<br>pharynx (21.1%)<br>muscle (100.0%)<br>intestine (0.0%)<br>rectum & anus (26.7%)<br>excretory system (0.0%)<br>coelomocyte (0.0%)	
8	miR-228	1. Reporter: expressed from mid embryo continuing through adulthood. Expressed in amphids, excretory cells, seam cells, vulva, body neurons, rectum and posterior intestine [1].<br>2. Reporter: P*mir-228*:: myristoylated-GFP for glia (allows distinguishing CEPsh glia from anterior labial glia and from the Amphid sheath glia) [7].	1. Reporter [1]:<br>Tissue level: seam cell; rectum<br>Cell level: amphid neurons; excretory cells; body neurons; posterior intestine<br>2. Reporter [7]:<br>Tissue level: glia	**Enriched tissue:**<br>P cell (sub)<br>intestine (sub)<br><br>**Expression status:**<br>neuron (0.0%)<br>glia (0.0%)<br>hypodermis (0.0%)<br>seam cell (0.0%)<br>P cell (0.0%)<br>pharynx (0.0%)<br>muscle (0.0%)<br>intestine (10.0%)<br>rectum & anus (0.0%)<br>excretory system (0.0%)<br>coelomocyte (0.0%)	Conclusion:<br>partially consistent

9	miR-231	1. Sequencing: in intestine and muscle (L1 stage, mime-seq, supplemental) [3].<br>2. Reporter: expressed from early embryo continuing through adulthood. In early embryos, expression is detected on the lateral sides and in mid embryo stages, expression is detected on the posterior part only. In larval and adult stages, expression is seen in posterior areas of intestine, rectum and tail hypodermis [1].	1. Sequencing [3]:<br>Tissue level: intestine; muscle<br>2. Reporter [1]:<br>Tissue level: rectum<br>Cell level: posterior intestine; tail hypodermis	**Enriched tissue:**<br>seam cell (pan)<br>P cell (pan)<br>intestine (pan)<br>rectum & anus (sub)<br><br>**Expression status:**<br>neuron (4.0%)<br>glia (5.0%)<br>hypodermis (10.4%)<br>seam cell (60.0%)<br>P cell (0.0%)<br>pharynx (12.6%)<br>muscle (1.1%)<br>intestine (80.0%)<br>rectum & anus (46.7%)<br>excretory system (0.0%)<br>coelomocyte (0.0%)	1. Partially consistent<br>2. Partially consistent<br><br>Conclusion:<br>partially consistent
10	miR-232	1. Reporter: expression detected from late embryos till adulthood. Expressed in excretory cells and canals [1].	1. Reporter [1]:<br>Cell level: excretory cells and canals	**Enriched tissue:**<br>neuron (sub)<br>excretory system (sub)<br><br>**Expression status:**<br>neuron (11.9%)<br>glia (0.0%)	Conclusion:<br>partially consistent

				hypodermis (0.0%)<br>seam cell (0.0%)<br>P cell (0.0%)<br>pharynx (0.0%)<br>muscle (0.0%)<br>intestine (0.0%)<br>rectum & anus (0.0%)<br>excretory system (16.7%)<br>coelomocyte (0.0%)	
11	miR-234	1. Reporter: neuronal cells in the head, the body and the tail (fosmid reporter, supplemental) [3].<br>2. Reporter: expressed from mid embryo continuing through adulthood. Expression seen in head nerves, specifically in 6 pairs in nerve ring and also dnc. Expressed also in tail nerves, specifically in 2 pairs and one single neuron with axon ventral to head. Expression seen in body nerves, specifically in CAN nerves. Also seen in amphid neurons in the pharynx; the cell body is posterior/lateral to posterior pharyngeal bulb (ppb) and axon runs to tip of head. Expression seen in I4, I5 and I6 neurons, cell body in ppb and axon runs to anterior pharyngeal bulb (apb) [1].<br>3. Reporter: including multiple neuron classes: sensory neurons (ADL, ASG, ALM, AVM,	1. Reporter [3]:<br>Cell level: neuronal cells in the head, the body and the tail<br>2. Reporter [1]:<br>Cell level: head nerves(6 pairs in nerve ring and also dnc); tail nerves(2 pairs and one single neuron with axon ventral to head); body nerves(CAN nerves); amphid neurons in the pharynx; I4, I5 and I6 neurons<br>3. Reporter [8]:<br>Cell level: sensory neurons (ADL, ASG, ALM, AVM, PHA, PVM), interneurons (AIM, PVQ, PVT, RID), motor neuron (HSN) and pharyngeal neurons (I4 and I6)	**Enriched tissue:**<br>intestine (sub)<br>rectum & anus (sub)<br><br>**Expression status:**<br>neuron (5.4%)<br>glia (0.0%)<br>hypodermis (0.0%)<br>seam cell (0.0%)<br>P cell (0.0%)<br>pharynx (9.5%)<br>muscle (0.0%)<br>intestine (35.0%)<br>rectum & anus (33.3%)<br>excretory system (16.7%)<br>coelomocyte (0.0%)	1. Partially consistent<br>2. Partially consistent<br>3. Partially consistent<br><br>Conclusion: partially consistent

		PHA, PVM), interneurons (AIM, PVQ, PVT, RID), motor neuron (HSN) and pharyngeal neurons (I4 and I6). [8]			
12	miR-235	1. Reporter: expressed from late embryos to adulthood. Expression detected in hypodermis, especially at L1-L2. Also detected in vulva, rectum and some amphid neurons [1].<br>2. Reporter: P*mir-235::gfp* is apparently expressed in the entire hypodermis from late embryogenesis to adult. In addition to the hypodermis, its expression was also seen in glia-like socket cells surrounding amphid neurons at L1 diapause (AmsoL/R) [9].	1. Reporter [1]:<br>Tissue level: hypodermis; rectum<br>Cell level: amphid neurons<br>2. Reporter [9]:<br>Tissue level: hypodermis<br>Cell level: glia-like socket cells(AmsoL/R)	**Enriched tissue:**<br>hypodermis (pan)<br>seam cell (pan)<br>P cell (pan)<br>rectum & anus (sub)<br><br>**Expression status:**<br>neuron (0.0%)<br>glia (25.0%)<br>hypodermis (95.8%)<br>seam cell (100.0%)<br>P cell (100.0%)<br>pharynx (0.0%)<br>muscle (0.0%)<br>intestine (0.0%)<br>rectum & anus (53.3%)<br>excretory system (50.0%)<br>coelomocyte (0.0%)	1. Partially consistent<br>2. Consistent<br><br>Conclusion:<br>consistent
13	miR-236	1. Sequencing: in intestine and pharynx (L1 stage, mime-seq, supplemental) [3].<br>2. Reporter: expressed from mid embryo to adulthood. Expression seen in intestine, rectal glands and dnc [1].	1. Sequencing [3]:<br>Tissue level: intestine; pharynx<br>2. Reporter [1]:<br>Tissue level: intestine<br>Cell level: rectal glands; dorsal nerve cord	**Enriched tissue:**<br>intestine(pan)<br><br>**Expression status:**<br>neuron (9.4%)	1. Partially consistent<br>2. Partially consistent

				glia (0.0%)<br>hypodermis (0.0%)<br>seam cell (0.0%)<br>P cell (0.0%)<br>pharynx (4.2%)<br>muscle (0.0%)<br>intestine (100.0%)<br>rectum & anus (6.7%)<br>excretory system (33.3%)<br>coelomocyte (0.0%)	Conclusion:<br>partially consistent
14	miR-244	1. Reporter: expressed in seam cells from late embryos till adults [1].	1. Reporter [1]:<br>Tissue level: seam cell	**Enriched tissue:**<br>neuron (pan)<br>glia (pan)<br>hypodermis (pan)<br>seam cell (pan)<br>P cell (pan)<br>pharynx (pan)<br>muscle (pan)<br><br>**Expression status:**<br>neuron (0.0%)<br>glia (0.0%)<br>hypodermis (0.0%)<br>seam cell (100.0%)<br>P cell (100.0%)<br>pharynx (2.1%)	Conclusion:<br>consistent

				muscle (0.0%)<br>intestine (0.0%)<br>rectum & anus (13.3%)<br>excretory system (33.3%)<br>coelomocyte (0.0%)	
15	miR-245	1. Sequencing and Reporter: in muscle, neurons and pharynx (L1 stage, mime-seq, supplemental), few neurons and pharynx (fosmid reporter, supplemental) [3].<br>2. Reporter: expressed from late embryos to adulthood. Expressed in a subset of pharyngeal muscles (pm3, 4 and 7). Expression seen also in head nerves and dnc. [1]	1. Sequencing and Reporter [3]:<br>Tissue level: muscle; neuron; pharynx<br>2. Reporter [1]:<br>Cell level: pharyngeal muscles (pm3, 4 and 7); head nerves and dorsal nerve cord	**Enriched tissue:**<br>coelomocyte (pan)<br><br>**Expression status:**<br>neuron (5.0%)<br>glia (0.0%)<br>hypodermis (0.0%)<br>seam cell (0.0%)<br>P cell (0.0%)<br>pharynx (6.3%)<br>muscle (0.0%)<br>intestine (0.0%)<br>rectum & anus (0.0%)<br>excretory system (0.0%)<br>coelomocyte (100.0%)	1. Partially consistent<br>2. Partially consistent<br><br>Conclusion:<br>partially consistent
16	miR-247-797	1. Sequencing: *mir-797*: in intestine (L1 stage, mime-seq, supplemental) [3].<br>2. Reporter: expression detected in pharynx from late embryo thorough L1. From L1 to L2/L3, expression is also detected in rectal glands and from L2 to L4, in distal tip cells [1].	1. Sequencing [3]:<br>*mir-797*:<br>Tissue level: intestine<br>2. Reporter [1]:<br>Tissue level: pharynx<br>Cell level: rectal glands	**Enriched tissue:**<br>pharynx (sub)<br>rectum & anus (sub)<br><br>**Expression status:**<br>neuron (0.0%)	1. Not consistent<br>2. Partially consistent<br><br>Conclusion:

				glia (0.0%)<br>hypodermis (0.0%)<br>seam cell (10.0%)<br>P cell (0.0%)<br>pharynx (13.7%)<br>muscle (0.0%)<br>intestine (0.0%)<br>rectum & anus (33.3%)<br>excretory system (0.0%)<br>coelomocyte (0.0%)	partially consistent
17	miR-251	1. Sequencing: in pharynx (L1 stage, mime-seq, supplemental) [3].<br>2. Reporter: expression seen from late embryos to adulthood. Expressed in pharyngeal muscles [1] .	1. Sequencing [3]:<br>Tissue level: pharynx<br>2. Reporter [1]:<br>Cell level: pharyngeal muscles	**Enriched tissue:**<br>pharynx (sub)<br><br>**Expression status:**<br>neuron (0.0%)<br>glia (0.0%)<br>hypodermis (0.0%)<br>seam cell (0.0%)<br>P cell (0.0%)<br>pharynx (18.9%)<br>muscle (0.0%)<br>intestine (0.0%)<br>rectum & anus (0.0%)<br>excretory system (0.0%)<br>coelomocyte (0.0%)	1. Partially consistent<br>2. Partially consistent<br><br>Conclusion: partially consistent
18	miR-252	1. Sequencing: in neurons (L1 stage, mime-seq,	1. Sequencing [3]:	**Enriched tissue:**	1. Not

		supplemental) [3].<br>2. Reporter: expressed in larval stages to adulthood in the pharyngeal glands, spermatheca and uterine cells, more specifically in a pair of cells closest to vulva. Also in head neurons [1].	Tissue level: neuron<br>2. Reporter [1]:<br>Cell level: pharyngeal glands; head neurons	intestine (pan)<br>excretory system (sub)<br><br>**Expression status:**<br>neuron (0.0%)<br>glia (0.0%)<br>hypodermis (0.0%)<br>seam cell (0.0%)<br>P cell (0.0%)<br>pharynx (6.3%)<br>muscle (0.0%)<br>intestine (100.0%)<br>rectum & anus (0.0%)<br>excretory system (33.3%)<br>coelomocyte (0.0%)<br><br>**Ubiquitous expression** (emb: 100.0%, L1: 5.0%)	consistent<br>2. Partially consistent<br><br>Conclusion: partially consistent
19	miR-259	1. Sequencing: in intestine and pharynx (L1 stage, mime-seq, supplemental) [3].<br>2. Reporter: expression seen from mid embryos to adults. In mid embryos, expression is detected in few cells in the posterior and anterior part. In larval stages and adults, expression is seen in rectal glands, pharyngeal/intestinal valve. Expression	1. Sequencing [3]:<br>Tissue level: intestine; pharynx<br>2. Reporter [1]:<br>Cell level: rectal glands; pharyngeal/intestinal valve	**Enriched tissue:**<br>pharynx (sub)<br>intestine (sub)<br>rectum & anus (sub)<br><br>**Expression status:**<br>neuron (0.0%)<br>glia (0.0%)	1. Partially consistent<br>2. Consistent<br><br>Conclusion: consistent

		detected also in the reproductive tract, specifically in somatic cells of the uterus, spermatheca and spermatheca-uterus valve [1].		hypodermis (0.0%)<br>seam cell (0.0%)<br>P cell (0.0%)<br>pharynx (8.4%)<br>muscle (0.0%)<br>intestine (20.0%)<br>rectum & anus (33.3%)<br>excretory system (0.0%)<br>coelomocyte (0.0%)	
20	miR-268	1. Reporter: expression seen from late embryos to adulthood in dnc, vnc, other head/body and tail nuclei nerves as well as pharyngeal nerve in anterior bulb [1].	1. Reporter [1]:<br>Cell level: dorsal nerve cord; ventral nerve cord; other head/body and tail nuclei nerves; pharyngeal nerve in anterior bulb	**Enriched tissue:**<br>pharynx (sub)<br><br>**Expression status:**<br>neuron (4.5%)<br>glia (0.0%)<br>hypodermis (0.0%)<br>seam cell (0.0%)<br>P cell (0.0%)<br>pharynx (8.4%)<br>muscle (0.0%)<br>intestine (0.0%)<br>rectum & anus (0.0%)<br>excretory system (0.0%)<br>coelomocyte (0.0%)	Conclusion:<br>partially consistent
21	miR-34	1. Sequencing and Reporter: in neurons, intestine, pharynx and muscle (L1 stage,	1. Sequencing and Reporter [3]:<br>Tissue level: neuron, intestine, pharynx;	**Enriched tissue:**<br>rectum & anus (sub)	Conclusion:<br>partially

		mime-seq, supplemental), many neurons (likely pharyngeal) in the head & pharynx (fosmid reporter, supplemental) [3].	muscle	**Expression status:**<br>neuron (1.0%)<br>glia (0.0%)<br>hypodermis (0.0%)<br>seam cell (0.0%)<br>P cell (0.0%)<br>pharynx (0.0%)<br>muscle (0.0%)<br>intestine (0.0%)<br>rectum & anus (26.7%)<br>excretory system (0.0%)<br>coelomocyte (0.0%)	consistent
22	miR-(42-)43-44	1. Reporter: expression seen in early embryos and continuing through adulthood. In the embryo, expression is seeing as stripes on the outside part of the embryo. In the mid embryo stage, gfp is seeing on dorsal and ventral part of the embryo, including head. Late embryos show complex expression. Strong expression in seam cells and vulva. Weaker expression in hypodermis. Also seeing in posterior intestine and rectum and dnc. In larval stages, expression was detected in hypodermal seam cells [1]	1. Reporter [1]:<br>Tissue level: seam cells; rectum<br>Cell level: posterior intestine; dorsal nerve cord	**Enriched tissue:**<br>neuron (pan)<br>glia (pan)<br>seam cell (pan)<br>P cell (pan)<br>rectum & anus (pan)<br><br>**Expression status:**<br>neuron (79.2%)<br>glia (62.5%)<br>hypodermis (62.5%)<br>seam cell (100.0%)<br>P cell (75.0%)	Conclusion: partially consistent

				pharynx (22.1%)<br>muscle (0.0%)<br>intestine (0.0%)<br>rectum & anus (93.3%)<br>excretory system (66.7%)<br>coelomocyte (0.0%)	
23	miR-45	1. Reporter: expression seen in all stages in the intestine, although it varies from anterior and/or posterior part. From late embryo till L1, strong expression is seen in the posterior part of the pharynx. Expresses also in dnc and a pair of nerves in the head at each side of the posterior pharyngeal bulb, in all stages and two amphids from L2 on. The late embryo stage shows a complex pattern of expression especially in lateral stripes. [1]	1. Reporter [1]:<br>Tissue level: intestine<br>Cell level: posterior pharynx; dorsal nerve cord and a pair of nerves in the head at each side of the posterior pharyngeal bulb	**Enriched tissue:**<br>hypodermis (pan)<br>seam cell (pan)<br>P cell (pan)<br>pharynx (pan)<br>intestine (pan)<br><br>**Expression status:**<br>neuron (3.0%)<br>glia (40.0%)<br>hypodermis (95.8%)<br>seam cell (95.0%)<br>P cell (100.0%)<br>pharynx (76.8%)<br>muscle (0.0%)<br>intestine (100.0%)<br>rectum & anus (73.3%)<br>excretory system (100.0%)<br>coelomocyte (0.0%)	Conclusion:<br>partially consistent
24	miR-48	1. Reporter: expressed from L1 to adulthood.	1. Reporter [1]:	**Enriched tissue:**	Conclusion:

		Expression seen in vulval cells, seam cells, head and tail neurons [1].	Tissue level: seam cell<br>Cell level: head and tail neurons	intestine (sub)<br><br>**Expression status:**<br>neuron (1.0%)<br>glia (0.0%)<br>hypodermis (0.0%)<br>seam cell (0.0%)<br>P cell (0.0%)<br>pharynx (4.2%)<br>muscle (0.0%)<br>intestine (50.0%)<br>rectum & anus (0.0%)<br>excretory system (0.0%)<br>coelomocyte (0.0%)	partially consistent
25	miR-49	No reference.	No reference.	**Enriched tissue:**<br>hypodermis (sub)<br>seam cell (pan)<br>intestine (pan)<br><br>**Expression status:**<br>neuron (3.0%)<br>glia (0.0%)<br>hypodermis (52.1%)<br>seam cell (95.0%)<br>P cell (0.0%)<br>pharynx (20.0%)	

				muscle (0.0%)<br>intestine (100.0%)<br>rectum & anus (33.3%)<br>excretory system (0.0%)<br>coelomocyte (0.0%)	
26	miR-51	1. Reporter: Expression detected from mid embryos to adults. In late embryos, expression is seen on one side of the embryo, ventral and mostly anterior. From late embryo to L1, expression is detected in canal cells and canal nerves. Also, from late embryos to adults, expression is detected in several nerves, including dnc and vnc. In addition, expression is detected in head muscles, coelomocytes and intestine [1].<br>2. Reporter: expression was restricted in expression to anterior and ventral cells identified as neurons, tail hypodermal cells, cells of the excretory system, and in the case of miR-51 GFP, the arcade cells [10].	1. Reporter [1]:<br>Tissue level: coelomocyte; intestine<br>Cell level: canal cells and canal nerves; dorsal nerve cord; ventral nerve cord; head muscles	**Enriched tissue:**<br>seam cell (pan)<br>P cell (pan)<br>intestine (pan)<br><br>**Expression status:**<br>neuron (81.7%)<br>glia (72.5%)<br>hypodermis (91.7%)<br>seam cell (100.0%)<br>P cell (100.0%)<br>pharynx (87.4%)<br>muscle (95.4%)<br>intestine (100.0%)<br>rectum & anus (100.0%)<br>excretory system (100.0%)<br>coelomocyte (100.0%)	Conclusion:<br>partially consistent
27	miR-52	1. Reporter: ubiquitously expressed in the soma (embryo stage) [10].	1. Reporter [10]:<br>ubiquitously expressed in the soma	**Expression status:**<br>neuron (100.0%)<br>glia (100.0%)<br>hypodermis (100.0%)	Conclusion:<br>consistent

				seam cell (100.0%)<br>P cell (100.0%)<br>pharynx (100.0%)<br>muscle (100.0%)<br>intestine (100.0%)<br>rectum & anus (100.0%)<br>excretory system (100.0%)<br>coelomocyte (100.0%)<br><br>**Ubiquitous expression** (emb: 100.0%, L1: 99.6%)	
28	miR-53	1. Reporter: two neurons and several additional muscle and hypodermal cells (L1, fosmid reporter, supplemental) [3].<br>2. Reporter: expression seen ubiquitously (except germline) from pre-comma stage to adulthood [1].<br>3. Reporter: ubiquitously expressed, but expression is weak in the gut and in anterior pharyngeal cells (embryo stage) [10].	1. Reporter [3]:<br>Cell level: two neurons; several additional muscle; several hypodermal cells<br>2. Reporter [1]:<br>ubiquitously expressed in the soma<br>3. Reporter [10]:<br>ubiquitously expressed	**Expression status:**<br>neuron (100.0%)<br>glia (100.0%)<br>hypodermis (100.0%)<br>seam cell (100.0%)<br>P cell (100.0%)<br>pharynx (100.0%)<br>muscle (100.0%)<br>intestine (100.0%)<br>rectum & anus (100.0%)<br>excretory system (100.0%)<br>coelomocyte (100.0%)<br><br>**Ubiquitous expression** (emb: 100.0%, L1: 99.6%)	1. Partially consistent<br>2. Consistent<br>3. Consistent<br><br>Conclusion: consistent

29	miR-54-55-56	1. Sequencing: *mir-55*: in neurons, intestine, pharynx and muscle, *mir-56*: in muscle and pharynx (L1 stage, mime-seq, supplemental) [3].<br>2. Reporter: expression seen from mid embryos to adulthood. Expressed strongly in nerve in head (amphid) and tail. Weak expression in external vulval cells, dnc and vnc is also detected [1].	1. Sequencing [3]:<br>Tissue level: neuron(*mir-55*); intestine(*mir-55*); pharynx(*mir-55-56*); muscle(*mir-55-56*)<br>2. Reporter [1]:<br>Cell level: nerve in head (amphid) and tail; dorsal nerve cord; ventral nerve cord	**Enriched tissue:**<br>neuron (pan)<br>muscle (pan)<br><br>**Expression status:**<br>neuron (100.0%)<br>glia (100.0%)<br>hypodermis (100.0%)<br>seam cell (100.0%)<br>P cell (100.0%)<br>pharynx (100.0%)<br>muscle (100.0%)<br>intestine (100.0%)<br>rectum & anus (100.0%)<br>excretory system (100.0%)<br>coelomocyte (100.0%)<br><br>**Ubiquitous expression** (emb: 55.7%, L1: 99.6%)	1. Partially consistent<br>2. Consistent<br><br>Conclusion: consistent
30	miR-57	1. Sequencing and reporter: in intestine and muscle (L1 stage, mime-seq, supplemental), Few motor neurons in addition to many other muscle and hypodermal cells (fosmid reporter, supplemental) [3].<br>2. Reporter: Expression seen in embryos starting at the comma stage and continuing throughout	1. Sequencing and reporter [3]:<br>Tissue level: intestine; muscle; hypodermis<br>Cell level: few motor neurons<br>2. Reporter [1]:<br>Tissue level: rectum<br>Cell level: posterior intestine; ventral nerve cord; ventral muscles; tail hypodermis	**Enriched tissue:**<br>hypodermis (sub)<br>seam cell (sub)<br>P cell (sub)<br>rectum & anus (pan)<br><br>**Expression status:**	1. Partially consistent<br>2. Partially consistent<br>3. Partially consistent

		adulthood. Posterior regions: intestine, ventral nerve cord, rectum, ventral muscles and tail hypodermis[1].<br>3. Reporter: The *mir-57* gene is expressed in posterior sublineages in the posterior regions of the animal. The cells from these sublineages lie in the posterior part of the embryo only and represent a wide variety of cell types, including tail seam cells, the hypodermal cells hyp10 and hyp11, the cells producing the tail spike, rectal cells, the P11/12 cells and even body wall muscle cells. Inspection of the movies beyond the comma stage also showed expression in the intestinal cells after elongation [11].	3. Reporter [11]:<br>Tissue level: body wall muscle; intestine<br>Cell level: rectum; tail seam cells; hypodermal cells (hyp10 and hyp11); the cells producing the tail spike; P11/12 cells	neuron (8.9%)<br>glia (5.0%)<br>hypodermis (22.9%, hyp10 and hyp11)<br>seam cell (20.0%)<br>P cell (66.7%, P11/12)<br>pharynx (0.0%)<br>muscle (6.9%)<br>intestine (0.0%)<br>rectum & anus (86.7%)<br>excretory system (16.7%)<br>coelomocyte (0.0%)	Conclusion:<br>partially consistent
31	miR-61-250	1. Sequencing: *mir-61*: in neurons and intestine, *mir-250*: neurons, intestine, pharynx and muscle (L1 stage, mime-seq, supplemental) [3].<br>2. Reporter: expression detected from early embryos to adults. Detected in dnc, two head nerves whose processes run ventral to each other and laterally towards the tip of the head. Also seen in tail nerves and posterior intestine[1].	1. Sequencing [3]:<br>Tissue level: neuron(*mir-61-250*); intestine(*mir-61-250*); pharynx(*mir-250*); muscle(*mir-250*)<br>2. Reporter [1]:<br>Cell level: dorsal nerve cord; two head nerves; tail nerves; posterior intestine	**Enriched tissue:**<br>hypodermis (sub)<br>seam cell (pan)<br>P cell (pan)<br>rectum & anus (sub)<br><br>**Expression status:**<br>neuron (0.0%)<br>glia (5.0%)<br>hypodermis (27.1%)<br>seam cell (80.0%)	1. Partially consistent<br>2. Not consistent<br><br>Conclusion:<br>partially consistent

				P cell (91.7%)<br>pharynx (6.3%)<br>muscle (0.0%)<br>intestine (0.0%)<br>rectum & anus (40.0%)<br>excretory system (16.7%)<br>coelomocyte (0.0%)<br><br>**Ubiquitous expression** (emb: 100.0%, L1: 10.8%)	
32	miR-63	1. Sequencing: in neurons, intestine and pharynx (L1 stage, mime-seq, supplemental) [3].<br>2. Reporter: Expression detected from comma stage to adults. Expression seen in intestine [1].	1. Sequencing [3]:<br>Tissue level: neuron, intestine and pharynx<br>2. Reporter [1]:<br>Tissue level: intestine	**Enriched tissue:**<br>intestine (sub)<br><br>**Expression status:**<br>neuron (0.0%)<br>glia (0.0%)<br>hypodermis (0.0%)<br>seam cell (0.0%)<br>P cell (0.0%)<br>pharynx (0.0%)<br>muscle (0.0%)<br>intestine (50.0%)<br>rectum & anus (0.0%)<br>excretory system (0.0%)<br>coelomocyte (0.0%)	1. Partially consistent<br>2. Partially consistent<br><br>Conclusion: partially consistent
33	miR-72	1. Sequencing: in neurons, intestine and pharynx	1. Sequencing [3]:	**Enriched tissue:**	1. Partially

		(L1 stage, mime-seq, supplemental) [3].<br>2. Reporter: expression seen in late embryos continuing to adulthood. Expression seen in pharynx and amphids, specifically 3 pairs of nerves with cell bodies just posterior to the anterior pharyngeal bulb [1].	Tissue level: neuron, intestine and pharynx<br>2. Reporter [1]:<br>Tissue level: pharynx<br>Cell level: amphid neurons	neuron (sub)<br><br>**Expression status:**<br>neuron (5.0%)<br>glia (0.0%)<br>hypodermis (0.0%)<br>seam cell (0.0%)<br>P cell (0.0%)<br>pharynx (0.0%)<br>muscle (0.0%)<br>intestine (0.0%)<br>rectum & anus (0.0%)<br>excretory system (0.0%)<br>coelomocyte (0.0%)<br><br>**Ubiquitous expression** (emb: 100.0%, L1: 2.2%)	consistent<br>2. Partially consistent<br><br>Conclusion: partially consistent
34	miR-73-74	1. Sequencing: *mir-73*: in intestine and pharynx, *mir-74*: in intestine (L1 stage, mime-seq, supplemental) [3].	1. Sequencing [3]:<br>Tissue level: intestine(*mir-73-74*); pharynx(*mir-73*)	**Enriched tissue:**<br>pharynx (pan)<br><br>**Expression status:**<br>neuron (100.0%)<br>glia (100.0%)<br>hypodermis (100.0%)<br>seam cell (100.0%)<br>P cell (100.0%)	Conclusion: partially consistent

				pharynx (100.0%)<br>muscle (86.2%)<br>intestine (100.0%)<br>rectum & anus (100.0%)<br>excretory system (100.0%)<br>coelomocyte (100.0%)<br><br>**Ubiquitous expression** (emb: 100.0%, L1: 97.5%)	
35	miR-75	1. Sequencing: in intestine (L1 stage, mime-seq, supplemental) [3].<br>2. Reporter: expression detected from mid embryos till adults. Expression seen exclusively in intestine [1].	1. Sequencing [3]:<br>Tissue level: intestine<br>2. Reporter [1]:<br>Tissue level: intestine	**Enriched tissue:**<br>intestine (pan)<br><br>**Expression status:**<br>neuron (0.0%)<br>glia (0.0%)<br>hypodermis (0.0%)<br>seam cell (0.0%)<br>P cell (0.0%)<br>pharynx (0.0%)<br>muscle (0.0%)<br>intestine (100.0%)<br>rectum & anus (0.0%)<br>excretory system (0.0%)<br>coelomocyte (0.0%)	1. Consistent<br>2. Consistent<br><br>Conclusion: consistent
36	miR-79	1. Sequencing: in muscle and pharynx (L1 stage, mime-seq, supplemental) [3].	1. Sequencing [3]:<br>Tissue level: muscle; pharynx	**Enriched tissue:**<br>hypodermis (pan)	1. Partially consistent

		2. Reporter: expressed from mid embryo continuing through adulthood. In mid embryos, expression is detected on lateral sides. Later in development, expression is detected in hypodermis [1].<br>3. Reporter: first detected in embryonic hypodermal (epidermal) tissue at 330 min after fertilization and continued to be expressed in the hypodermis through larval development [12].	2. Reporter [1]:<br>Tissue level: hypodermis<br>3. Reporter [12]:<br>Tissue level: hypodermis	seam cell (pan)<br>P cell (pan)<br>pharynx (sub)<br><br>**Expression status:**<br>neuron (3.5%)<br>glia (5.0%)<br>hypodermis (81.2%)<br>seam cell (100.0%)<br>P cell (100.0%)<br>pharynx (42.1%)<br>muscle (0.0%)<br>intestine (0.0%)<br>rectum & anus (20.0%)<br>excretory system (16.7%)<br>coelomocyte (0.0%)	2. Consistent<br>3. Consistent<br><br>Conclusion:<br>consistent
37	miR-790	1. Sequencing: in neurons (L1 stage, mime-seq, supplemental) [3].	1. Sequencing [3]:<br>Tissue level: neuron	**Enriched tissue:**<br>glia (sub)<br>seam cell (sub)<br>rectum & anus (sub)<br><br>**Expression status:**<br>neuron (1.0%)<br>glia (20.0%)<br>hypodermis (6.2%)<br>seam cell (20.0%)	Conclusion:<br>partially consistent

				P cell (0.0%)<br>pharynx (2.1%)<br>muscle (0.0%)<br>intestine (0.0%)<br>rectum & anus (46.7%)<br>excretory system (0.0%)<br>coelomocyte (0.0%)	
38	miR-793	1. Sequencing and Reporter: in neurons and pharynx (L1 stage, mime-seq, supplemental), ASEs and few other neurons (fosmid reporter, supplemental) [3].<br>2. Reporter: expressed from late embryo stage till adults. Expression seen in head and tail neurons [1].	1. Sequencing and Reporter [3]:<br>Tissue level: neuron; pharynx<br>2. Reporter [1]:<br>Cell level: head and tail neurons	**Enriched tissue:**<br>neuron (sub)<br><br>**Expression status:**<br>neuron (6.9%)<br>glia (0.0%)<br>hypodermis (0.0%)<br>seam cell (0.0%)<br>P cell (0.0%)<br>pharynx (7.4%)<br>muscle (0.0%)<br>intestine (0.0%)<br>rectum & anus (0.0%)<br>excretory system (0.0%)<br>coelomocyte (0.0%)	1. Partially consistent<br>2. Partially consistent<br><br>Conclusion: partially consistent
39	miR-794	1. Sequencing: in neurons and muscle (L1 stage, mime-seq, supplemental) [3].<br>2. Reporter: expressed from late embryos to adulthood. Expressed in head and body wall	1. Sequencing [3]:<br>Tissue level: neuron; muscle<br>2. Reporter [1]:<br>Tissue level: body wall muscle; intestine	**Enriched tissue:**<br>neuron (pan)<br><br>**Expression status:**	1. Partially consistent<br>2. Partially consistent

		muscle and intestine (mosaic) [1].		neuron (97.5%)<br>glia (0.0%)<br>hypodermis (0.0%)<br>seam cell (0.0%)<br>P cell (0.0%)<br>pharynx (26.3%)<br>muscle (31.0%)<br>intestine (50.0%)<br>rectum & anus (46.7%)<br>excretory system (0.0%)<br>coelomocyte (0.0%)	Conclusion:<br>partially consistent
40	miR-795	No reference.	No reference.	**Enriched tissue:**<br>neuron (sub)<br>rectum & anus (sub)<br><br>**Expression status:**<br>neuron (1.5%)<br>glia (0.0%)<br>hypodermis (0.0%)<br>seam cell (0.0%)<br>P cell (0.0%)<br>pharynx (0.0%)<br>muscle (0.0%)<br>intestine (0.0%)<br>rectum & anus (0.0%)<br>excretory system (0.0%)	

				coelomocyte (0.0%)	
41	miR-796	No reference.	No reference.	**Expression status:**<br>neuron (0.0%)<br>glia (0.0%)<br>hypodermis (0.0%)<br>seam cell (0.0%)<br>P cell (0.0%)<br>pharynx (0.0%)<br>muscle (0.0%)<br>intestine (0.0%)<br>rectum & anus (0.0%)<br>excretory system (0.0%)<br>coelomocyte (0.0%)	
42	miR-80	1. Reporter: Expressed from late embryos to adulthood. Expressed in excretory cells, uterus, vulva, dnc, distal tip cells, posterior intestine, amphid neurons, head and body wall muscle [1].	1. Reporter [1]:<br>Tissue level: body wall muscle<br>Cell level: excretory cells; dorsal nerve cord; distal tip cells; posterior intestine; amphid neurons	**Enriched tissue:**<br>pharynx (sub)<br><br>**Expression status:**<br>neuron (0.0%)<br>glia (0.0%)<br>hypodermis (0.0%)<br>seam cell (0.0%)<br>P cell (0.0%)<br>pharynx (3.2%)<br>muscle (0.0%)<br>intestine (0.0%)<br>rectum & anus (0.0%)	Conclusion:<br>not consistent

				excretory system (0.0%)<br>coelomocyte (0.0%)	
43	miR-81	1. Sequencing: in muscle (L1 stage, mime-seq, supplemental) [3].	1. Sequencing [3]:<br>Tissue level: muscle	**Enriched tissue:**<br>pharynx (sub)<br>excretory system (sub)<br><br>**Expression status:**<br>neuron (0.5%)<br>glia (2.5%)<br>hypodermis (6.2%)<br>seam cell (0.0%)<br>P cell (0.0%)<br>pharynx (36.8%)<br>muscle (0.0%)<br>intestine (0.0%)<br>rectum & anus (13.3%)<br>excretory system (50.0%)<br>coelomocyte (0.0%)	Conclusion:<br>not consistent
44	miR-82	1. Sequencing: in pharynx (L1 stage, mime-seq, supplemental) [3].<br>2. Reporter: two amphid neurons, excretory gland cell, subset of neurons in the tail; expressed at all stages, highest expression in L4s and adults [5].	1. Sequencing [3]:<br>Tissue level: pharynx<br>2. Reporter [5]:<br>Cell level: two amphid neurons; excretory gland cell; subset of neurons in the tail	**Enriched tissue:**<br>pharynx (sub)<br>coelomocyte (pan)<br><br>**Expression status:**<br>neuron (0.0%)<br>glia (0.0%)<br>hypodermis (0.0%)	1. Partially consistent<br>2. Not consistent<br><br>Conclusion:<br>partially consistent

				seam cell (0.0%)<br>P cell (0.0%)<br>pharynx (24.2%)<br>muscle (0.0%)<br>intestine (0.0%)<br>rectum & anus (0.0%)<br>excretory system (0.0%)<br>coelomocyte (100.0%)	
45	miR-83	1. Sequencing and Reporter: in neurons, intestine and pharynx (L1 stage, mime-seq, supplemental), Neurons in the head and the tail (very bright), pharynx and intestine (fosmid reporter, supplemental) [3].<br>2. Reporter: expressed from early embryo continuing through adulthood. Expression detected in many nerves from head and tail, the most obvious are amphids and phasmids. Also, in dnc, vnc, canal nerves, intestine, rectal gland, spermatheca/uterine valve [1].<br>3. Reporter: we observed that the *Pmir-83*::GFP reporter was widely expressed at all stages of development and across a range of tissues such as the intestine, neurons, and body wall muscles [13].	1. Sequencing and Reporter [3]:<br>Tissue level: neuron; intestine; pharynx<br>2. Reporter [1]:<br>Tissue level: neuron; intestine<br>Cell level: rectal gland<br>3. Reporter [13]:<br>Tissue level: intestine; neuron; body wall muscle	**Enriched tissue:**<br>neuron (sub)<br>intestine (pan)<br>**Expression status:**<br>neuron (38.1%)<br>glia (0.0%)<br>hypodermis (2.1%)<br>seam cell (0.0%)<br>P cell (0.0%)<br>pharynx (1.1%)<br>muscle (0.0%)<br>intestine (100.0%)<br>rectum & anus (20.0%)<br>excretory system (0.0%)<br>coelomocyte (0.0%)	1. Partially consistent<br>2. Partially consistent<br>3. Partially consistent<br><br>Conclusion:<br>partially consistent
46	miR-84	1. Sequencing and Reporter: in neurons (L1 stage, mime-seq, supplemental), Neurons in	1. Sequencing and Reporter [3]:<br>Tissue level: neuron	**Expression status:**<br>neuron (4.0%)	1. Partially consistent

		the head (close to pharynx) and in the tail (fosmid reporter, supplemental) [3].<br>2. Reporter: expression seen from L1 to adult stages. Strong expression seen is pharynx, vulva and rectal glands. Weak expression seen in hypodermis and seam cells. Also seen in canal nerves, spermatheca/uterine valve and head neuron. Posterior intestine expression also detected [1].<br>3. Reporter: mir-84::gfp was first observed in the somatic gonad in larval stage 1 (L1)[14].<br>4. Reporter: mir-84 was expressed at the L1 stage in the Z1 and Z4 cells of the gonad [15].	2. Reporter [1]:<br>Tissue level: pharynx<br>Cell level: rectal glands; canal nerves; head neuron; posterior intestine	glia (0.0%)<br>hypodermis (0.0%)<br>seam cell (0.0%)<br>P cell (0.0%)<br>pharynx (6.3%)<br>muscle (0.0%)<br>intestine (0.0%)<br>rectum & anus (0.0%)<br>excretory system (0.0%)<br>coelomocyte (0.0%)	2. Partially consistent<br><br>Conclusion: partially consistent
47	miR-86	1. Reporter: ventral nerve cord (some cells), dorsal nerve cord, subset of neurons in the tail, many neurons in the nerve ring; expressed at all stages, highest expression in L2s [5].	1. Reporter [5]:<br>Cell level: ventral nerve cord (some cells); dorsal nerve cord; subset of neurons in the tail; many neurons in the nerve ring	**Expression status:**<br>neuron (0.0%)<br>glia (0.0%)<br>hypodermis (0.0%)<br>seam cell (0.0%)<br>P cell (0.0%)<br>pharynx (0.0%)<br>muscle (0.0%)<br>intestine (0.0%)<br>rectum & anus (0.0%)<br>excretory system (0.0%)<br>coelomocyte (0.0%)	Conclusion: not consistent
48	miR-90	1. Sequencing: in neurons and muscle (L1 stage,	1. Sequencing[3]:	**Expression status:**	1. Partially

		mime-seq, supplemental) [3].<br>2. Reporter: expression seen in mid embryos, specifically on the lateral sides of the embryo, continuing to adulthood. GFP expression is seeing in head and body wall muscles, amphids and phasmids, including other nerve nuclei in head but cannot see processes. Also, expressed in vnc and lateral nerves of body. Expression seen also in vulval muscles and cells at uterus, proximal to the vulva [1].	Tissue level: neuron; muscle<br>2. Reporter[1].:<br>Tissue level: body wall muscle<br>Cell level: amphid and phasmid neurons; ventral nerve cord and lateral nerves of body	neuron (100.0%)<br>glia (100.0%)<br>hypodermis (100.0%)<br>seam cell (100.0%)<br>P cell (100.0%)<br>pharynx (100.0%)<br>muscle (100.0%)<br>intestine (100.0%)<br>rectum & anus (100.0%)<br>excretory system (100.0%)<br>coelomocyte (100.0%)<br><br>**Ubiquitous expression** (emb: 100.0%, L1: 99.6%)	consistent<br>2. Partially consistent<br><br>Conclusion: partially consistent

References

1. Martinez, N.J. *et al.* Genome-scale spatiotemporal analysis of Caenorhabditis elegans microRNA promoter activity. *Genome Res.* **18**, 2005-2015 (2008).
2. McCulloch, K.A. & Rougvie, A.E. Caenorhabditis elegans period homolog lin-42 regulates the timing of heterochronic miRNA expression. *Theor. Biol. Med. Model.* **111**, 15450-15455 (2014).
3. Alberti, C. *et al.* Cell-type specific sequencing of microRNAs from complex animal tissues. *Nat. Methods* **15**, 283-289 (2018).
4. Andachi, Y. & Kohara, Y. A whole-mount in situ hybridization method for microRNA detection in Caenorhabditis elegans. *RNA* **22**, 1099-1106 (2016).
5. Isik, M., Korswagen, H.C. & Berezikov, E. Expression patterns of intronic microRNAs in Caenorhabditis elegans. *Silence* **1**, 5 (2010).
6. Clark, A.M. *et al.* The microRNA miR-124 controls gene expression in the sensory nervous system of Caenorhabditis elegans. *Nucleic Acids Research* **38**, 3780-3793 (2010).
7. Rapti, G., Li, C., Shan, A., Lu, Y. & Shaham, S. Glia initiate brain assembly through noncanonical Chimaerin-Furin axon guidance in C. elegans. *Nature Neuroscience* **20**, 1350-1360 (2017).
8. Snieckute, G. *et al.* mir-234 controls neuropeptide release at the Caenorhabditis elegans neuromuscular junction. *Molecular and Cellular Neuroscience* **98**, 70-81 (2019).
9. Kasuga, H., Fukuyama, M., Kitazawa, A., Kontani, K. & Katada, T. The microRNA miR-235 couples blast-cell quiescence to the nutritional state. *Nature* **497**, 503-506 (2013).
10. Shaw, W.R., Armisen, J., Lehrbach, N.J. & Miska, E.A. The conserved miR-51 microRNA family is redundantly required for embryonic development and pharynx attachment in Caenorhabditis elegans. *Genetics* **185**, 897-905 (2010).
11. Zhao, Z. *et al.* A negative regulatory loop between microRNA and Hox gene controls posterior identities in Caenorhabditis elegans. *PLoS Genetics* **6**, e1001089 (2010).
12. Pedersen, M.E. *et al.* An epidermal microRNA regulates neuronal migration through control of the cellular glycosylation state. *Science* **341**, 1404-1408 (2013).
13. Dzakah, E.E. *et al.* Loss of miR-83 extends lifespan and affects target gene expression in an age-dependent manner in Caenorhabditis elegans. *J Genet Genomics* **45**, 651-662 (2018).
14. Johnson, S.M. *et al.* RAS is regulated by the let-7 microRNA family. *Cell* **120**, 635-647 (2005).
15. Esquela-Kerscher, A. *et al.* Post-embryonic expression of C. elegans microRNAs belonging to the lin-4 and let-7 families in the hypodermis and the reproductive system. *Developmental Dynamics* **234**, 868-877 (2005).
