# Supplementary material for: A lineage-resolved cartography of microRNA promoter activity in C. elegans empowers multidimensional developmental analysis
Source: Nat Commun. 2024 Mar 30;15:2783. doi: 10.1038/s41467-024-47055-4 (PMC10981687; doi:10.1038/s41467-024-47055-4)
Supplement: Supplementary file 11 — Supplementary Dataset 8 [file 41467_2024_47055_MOESM11_ESM.pdf]

#	miRNA-pr	Tissue specificity detected in this study (embryo and L1 stage)	Related pattern description in the literature using approaches excepting sequencing (Previous studies are numbered and the method used to determine the expression is indicated)	Comment
1	let-7	hypodermis (sub);<br>intestine (pan);<br>coelomocyte (pan)	1. Reporter [1]: expression detected from late embryos to adults. In late embryos to L1, expression is seen in dnc and intestine.<br>2. Reporter [2]: expression observed in a variety of tissues (hypodermis, intestine, body wall muscle, pharynx, vulva, and nervous system) beginning in different larval stages. For example, gut and pharyngeal expression began in the L1, whereas hypodermal seam cells, a site of relevance for the *lin-42* heterochronic phenotype, were not observed until the L3 stage.	2/3 undetected specificity
2	lin-4	pharynx (sub)	1. Reporter [1]: expression seen from late L1 to adult stages. Weak expression detected ubiquitously (except germline). Stronger in pharynx, vulva, vulval muscle, body wall muscle.	0/1 undetected specificity
3	miR-1	pharynx (pan);<br>muscle (pan)	1. Reporter [1]: expression detected from early embryos to adults specifically in pharynx and precursors.<br>2. In situ hybridization [3]: observed in the body-wall muscle at the adult stage. Additionally, *mir-1* miRNA was detected in the anal depressor muscle and the pharynx, which is a muscular tube used to suck food. The miRNA was also detected in sex-specific muscles, i.e., the vulval muscle in hermaphrodites and the diagonal muscle in males. Analyses of larvae by the method also gave signals in these muscles at each of the four larval stages (Fig. 1D–G). Pharyngeal signals were detected in embryos at the 500-cell stage and later (Fig. 1H–K).	0/2 undetected specificity

4	miR-1018			0/0 undetected specificity
5	miR-1022	seam cell (sub);<br>pharynx (sub);<br>rectum & anus (sub)		3/3 undetected specificity
6	miR-124	neuron (sub)	1. Reporter [4]: Neurons in the head (close to pharynx) and in the tail (fosmid reporter, supplemental).<br>2. Reporter [5]: Nervous system.<br>3. Reporter [6]: from mid-embryogenesis (350min post-fertilization), when neuronal differentiation begins, throughout development and in adults. We detected *mir-124* promoter::*gfp* expression in 40 of the 302 neurons in C. elegans (some of these as sensory neurons most of which are ciliated [AWC, AWA, AWB, ASH, ASI, ASK, PVQ (not ciliated), ASE, PHA, PHB, PVD (not ciliated), IL1, ADE, PDE).	0/1 undetected specificity
7	miR-2	neuron (pan);<br>muscle (pan)	1. Reporter [1]: expressed from late embryos to adulthood. Strong expression detected in many nerves, nerve ring, dnc, vnc an also nerves in the tail.	1/2 undetected specificity

8	miR-228	P cell (sub); intestine (sub)	1. Reporter [1]: expressed from mid embryo continuing through adulthood. Expressed in amphids, excretory cells, seam cells, vulva, body neurons, rectum and posterior intestine.	1/2 undetected specificity
9	miR-231	seam cell (pan); P cell (pan); intestine (pan); rectum & anus (sub)	1. Reporter [1]: expressed from early embryo continuing through adulthood. In early embryos, expression is detected on the lateral sides and in mid embryo stages, expression is detected on the posterior part only. In larval and adult stages, expression is seen in posterior areas of intestine, rectum and tail hypodermis.	2/4 undetected specificity
10	miR-232	neuron (sub); excretory system (sub)	1. Reporter [1]: expression detected from late embryos till adulthood. Expressed in excretory cells and canals.	1/2 undetected specificity
11	miR-234	intestine (sub); rectum & anus (sub)		2/2 undetected specificity
12	miR-235	hypodermis (pan); seam cell (pan); P cell (pan); rectum & anus (sub)	1. Reporter [1]: expressed from late embryos to adulthood. Expression detected in hypodermis, especially at L1-L2. Also detected in vulva, rectum and some amphid neurons.<br>2. Reporter [7]: P*mir-235::gfp* is apparently expressed in the entire hypodermis from late embryogenesis to adult. In addition to the hypodermis, its expression was also seen in glia-like socket cells surrounding amphid neurons at L1 diapause (AmsoL/R).	2/4 undetected specificity

13	miR-236	intestine (pan)	1. Reporter [1]: expressed from mid embryo to adulthood. Expression seen in intestine, rectal glands and dnc.	0/1 undetected specificity
14	miR-244	neuron (pan);<br>glia (pan);<br>hypodermis (pan);<br>seam cell (pan);<br>P cell (pan);<br>pharynx (pan);<br>muscle (pan)	1. Reporter [1]: expressed in seam cells from late embryos till adults.	6/7 undetected specificity
15	miR-245	coelomocyte (pan)		1/1 undetected specificity
16	miR-247-797	pharynx (sub);<br>rectum & anus (sub)	1. Reporter [1]: expression detected in pharynx from late embryo thorough L1. From L1 to L2/L3, expression is also detected in rectal glands and from L2 to L4, in distal tip cells.	0/2 undetected specificity
17	miR-251	pharynx (sub)	1. Reporter [1]: expression seen from late embryos to adulthood. Expressed in pharyngeal muscles.	0/1 undetected specificity
18	miR-252	intestine (pan);<br>excretory system (sub)		2/2 undetected specificity

19	miR-259	pharynx (sub); intestine (sub); rectum & anus (sub)	1. Reporter [1]: expression seen from mid embryos to adults. In mid embryos, expression is detected in few cells in the posterior and anterior part. In larval stages and adults, expression is seen in rectal glands, pharyngeal/intestinal valve. Expression detected also in the reproductive tract, specifically in somatic cells of the uterus, spermatheca and spermatheca-uterus valve.	1/3 undetected specificity
20	miR-268	pharynx (sub)	1. Reporter [1]: expression seen from late embryos to adulthood in dnc, vnc, other head/body and tail nuclei nerves as well as pharyngeal nerve in anterior bulb.	0/1 undetected specificity
21	miR-34	rectum & anus (sub)		1/1 undetected specificity
22	miR-(42-)43-44	neuron (pan); glia (pan); seam cell (pan); P cell (pan); rectum & anus (pan)	1. Reporter [1]: expression seen in early embryos and continuing through adulthood. In the embryo, expression is seeing as stripes on the outside part of the embryo. In the mid embryo stage, gfp is seeing on dorsal and ventral part of the embryo, including head. Late embryos show complex expression. Strong expression in seam cells and vulva. Weaker expression in hypodermis. Also seeing in posterior intestine and rectum and dnc. In larval stages, expression was detected in hypodermal seam cells.	3/5 undetected specificity
23	miR-45	hypodermis (pan); seam cell (pan); P cell (pan); pharynx (pan); intestine (pan)	1. Reporter [1]: expression seen in all stages in the intestine, although it varies from anterior and/or posterior part. From late embryo till L1, strong expression is seen in the posterior part of the pharynx. Expresses also in dnc and a pair of nerves in the head at each side of the posterior pharyngeal bulb, in all stages and two amphids from L2 on. The late embryo stage shows a complex pattern of expression especially in lateral stripes.	4/5 undetected specificity

24	miR-48	intestine (sub)		1/1 undetected specificity
25	miR-49	hypodermis (sub);<br>seam cell (pan);<br>intestine (pan)		3/3 undetected specificity
26	miR-51	seam cell (pan);<br>P cell (pan);<br>intestine (pan)	1. Reporter [1]: expression detected from mid embryos to adults. In late embryos, expression is seen on one side of the embryo, ventral and mostly anterior. From late embryo to L1, expression is detected in canal cells and canal nerves. Also, from late embryos to adults, expression is detected in several nerves, including dnc and vnc. In addition, expression is detected in head muscles, coelomocytes and intestine.	2/3 undetected specificity
27	miR-52			0/0 undetected specificity
28	miR-53			0/0 undetected specificity
29	miR-54-55-56	neuron (pan);<br>muscle (pan)	1. Reporter [1]: expression seen from mid embryos to adulthood. Expressed strongly in nerve in head (amphid) and tail.	1/2 undetected specificity
30	miR-57	hypodermis (sub);<br>seam cell (sub);<br>P cell (sub);	1. Reporter [4]: Few motor neurons in addition to many other muscle and hypodermal cells (fosmid reporter, supplemental).<br>2. Reporter [1]: Expression seen in embryos starting at the comma stage and continuing throughout adulthood. Posterior regions: intestine, ventral nerve cord, rectum, ventral muscles and tail	1/4 undetected specificity

		rectum & anus (pan)	hypodermis.<br><br>3. Reporter [8]: The *mir-57* gene is expressed in posterior sublineages in the posterior regions of the animal. The cells from these sublineages lie in the posterior part of the embryo only and represent a wide variety of cell types, including tail seam cells, the hypodermal cells hyp10 and hyp11, the cells producing the tail spike, rectal cells, the P11/12 cells and even body wall muscle cells. Inspection of the movies beyond the comma stage also showed expression in the intestinal cells after elongation.	
31	miR-61-250	hypodermis (sub);<br><br>seam cell (pan);<br><br>P cell (pan);<br><br>rectum & anus (sub)		4/4 undetected specificity
32	miR-63	intestine (sub)	1. Reporter [1]: expression detected from comma stage to adults. Expression seen in intestine.	0/1 undetected specificity
33	miR-72	neuron (sub)	1. Reporter [1]: expression seen in late embryos continuing to adulthood. Expression seen in pharynx and amphids, specifically 3 pairs of nerves with cell bodies just posterior to the anterior pharyngeal bulb.	0/1 undetected specificity
34	miR-73-74	pharynx (pan)		1/1 undetected specificity
35	miR-75	intestine (pan)	1. Reporter [1]: expression detected from mid embryos till adults. Expression seen exclusively in	0/1 undetected

			intestine.	specificity
36	miR-79	hypodermis (pan);<br>seam cell (pan);<br>P cell (pan);<br>pharynx (sub)	1. Reporter [1]: expressed from mid embryo continuing through adulthood. In mid embryos, expression is detected on lateral sides. Later in development, expression is detected in hypodermis.<br>2. Reporter [9]: first detected in embryonic hypodermal (epidermal) tissue at 330 min after fertilization and continued to be expressed in the hypodermis through larval development.	3/4 undetected specificity
37	miR-790	glia (sub);<br>seam cell (sub);<br>rectum & anus (sub)		3/3 undetected specificity
38	miR-793	neuron (sub)	1. Reporter [4]: ASEs and few other neurons (fosmid reporter, supplemental).<br>2. Reporter [1]: expressed from late embryo stage till adults. Expression seen in head and tail neurons.	0/1 undetected specificity
39	miR-794	neuron (pan)		1/1 undetected specificity
40	miR-795	neuron (sub);<br>rectum & anus (sub)		2/2 undetected specificity
41	miR-796			0/0 undetected specificity

42	miR-80	pharynx (sub)		1/1 undetected specificity
43	miR-81	pharynx (sub);<br>excretory system (sub)		2/2 undetected specificity
44	miR-82	pharynx (sub);<br>coelomocyte (pan)		2/2 undetected specificity
45	miR-83	neuron (sub);<br>intestine (pan)	1. Reporter [4]: Neurons in the head and the tail (very bright), pharynx and intestine (fosmid reporter, supplemental).<br>2. Reporter [1]: expressed from early embryo continuing through adulthood. Expression detected in many nerves from head and tail, the most obvious are amphids and phasmids. Also, in dnc, vnc, canal nerves, intestine, rectal gland, spermatheca/uterine valve.<br>3. Reporter [10]: we observed that the *Pmir-83*::GFP reporter was widely expressed at all stages of development and across a range of tissues such as the intestine, neurons, and body wall muscles.	0/2 undetected specificity
46	miR-84			0/0 undetected specificity
47	miR-86			0/0 undetected specificity

48	miR-90			0/0 undetected specificity

References

1. Martinez, N.J. *et al.* Genome-scale spatiotemporal analysis of Caenorhabditis elegans microRNA promoter activity. *Genome Res* **18**, 2005-2015 (2008).
2. McCulloch, K.A. & Rougvie, A.E. Caenorhabditis elegans period homolog lin-42 regulates the timing of heterochronic miRNA expression. *Theor. Biol. Med. Model.* **111**, 15450-15455 (2014).
3. Andachi, Y. & Kohara, Y. A whole-mount in situ hybridization method for microRNA detection in Caenorhabditis elegans. *RNA* **22**, 1099-1106 (2016).
4. Alberti, C. *et al.* Cell-type specific sequencing of microRNAs from complex animal tissues. *Nat Methods* **15**, 283-289 (2018).
5. Isik, M., Korswagen, H.C. & Berezikov, E. Expression patterns of intronic microRNAs in Caenorhabditis elegans. *Silence* **1**, 5 (2010).
6. Clark, A.M. *et al.* The microRNA miR-124 controls gene expression in the sensory nervous system of Caenorhabditis elegans. *Nucleic Acids Research* **38**, 3780-3793 (2010).
7. Kasuga, H., Fukuyama, M., Kitazawa, A., Kontani, K. & Katada, T. The microRNA miR-235 couples blast-cell quiescence to the nutritional state. *Nature* **497**, 503-506 (2013).
8. Zhao, Z. *et al.* A negative regulatory loop between microRNA and Hox gene controls posterior identities in Caenorhabditis elegans. *PLoS Genetics* **6**, e1001089 (2010).
9. Pedersen, M.E. *et al.* An epidermal microRNA regulates neuronal migration through control of the cellular glycosylation state. *Science* **341**, 1404-1408 (2013).
10. Dzakah, E.E. *et al.* Loss of miR-83 extends lifespan and affects target gene expression in an age-dependent manner in Caenorhabditis elegans. *J Genet Genomics* **45**, 651-662 (2018).
