# Supplementary material for: A lineage-resolved cartography of microRNA promoter activity in C. elegans empowers multidimensional developmental analysis
Source: Nat Commun. 2024 Mar 30;15:2783. doi: 10.1038/s41467-024-47055-4 (PMC10981687; doi:10.1038/s41467-024-47055-4)
Supplement: Supplementary file 22 — Reporting Summary [file 41467_2024_47055_MOESM22_ESM.pdf]

nature portfolio

Corresponding author(s): Yongbin Li and Zhuo Du

Last updated by author(s): Feb 28, 2024

Reporting Summary

Nature Portfolio wishes to improve the reproducibility of the work that we publish. This form provides structure for consistency and transparency in reporting. For further information on Nature Portfolio policies, see our Editorial Policies and the Editorial Policy Checklist.

Statistics

For all statistical analyses, confirm that the following items are present in the figure legend, table legend, main text, or Methods section.

n/a	Confirmed	
☐	☒	The exact sample size (*n*) for each experimental group/condition, given as a discrete number and unit of measurement
☐	☒	A statement on whether measurements were taken from distinct samples or whether the same sample was measured repeatedly
☐	☒	The statistical test(s) used AND whether they are one- or two-sided<br>*Only common tests should be described solely by name; describe more complex techniques in the Methods section.*
☒	☐	A description of all covariates tested
☐	☒	A description of any assumptions or corrections, such as tests of normality and adjustment for multiple comparisons
☐	☒	A full description of the statistical parameters including central tendency (e.g. means) or other basic estimates (e.g. regression coefficient) AND variation (e.g. standard deviation) or associated estimates of uncertainty (e.g. confidence intervals)
☐	☒	For null hypothesis testing, the test statistic (e.g. *F*, *t*, *r*) with confidence intervals, effect sizes, degrees of freedom and *P* value noted<br>*Give P values as exact values whenever suitable.*
☒	☐	For Bayesian analysis, information on the choice of priors and Markov chain Monte Carlo settings
☒	☐	For hierarchical and complex designs, identification of the appropriate level for tests and full reporting of outcomes
☐	☒	Estimates of effect sizes (e.g. Cohen's *d*, Pearson's *r*), indicating how they were calculated

Our web collection on statistics for biologists contains articles on many of the points above.

Software and code

Policy information about availability of computer code

Data collection	MetaMorph software, version 7.10, was used to acquire 3D time-lapse confocal images of C. elegans embryogenesis. Leica Application Suite X (LAS X), version 1.4.4, was used to acquire 3D confocal images of C. elegans L1 larvae.
Data analysis	An open-source image analysis software, StarryNite (http://starrynite.sourceforge.net), was used to automate cell identification and tracing to construct the embryonic cell lineage. An open-source lineage visualization software, AceTree (http://acetree.sourceforge.net), was used for lineage curation. The open-source image analysis pipeline CellExplorer was used to straighten 3D image stacks of L1s computationally, and VANO was used to annotate nuclear identities (https://sites.google.com/a/cellexplorer.org/vano/software-downloads). The open-source software, Python version 3.7.1, Perl version 5.30.3, and R version 3.6.1, was used to quantify and analyze microRNA expression, conduct statistic tests, and perform other bioinformatics. GraphPad Prism 9 was used to perform statistical tests and generate some figures. An Open source software, POV-Ray version 3.7, was used to visualize the cellular expression of microRNA reporters in the embryo and at the L1 stage. The CRISPR Design Tool (http://zlab.bio/guide-design-resources) was used to choose single-guide RNAs (sgRNAs). An open-source image analysis software, ImageJ 1.48v, was used for image analysis.

For manuscripts utilizing custom algorithms or software that are central to the research but not yet described in published literature, software must be made available to editors and reviewers. We strongly encourage code deposition in a community repository (e.g. GitHub). See the Nature Portfolio guidelines for submitting code & software for further information.

Data

Policy information about availability of data

All manuscripts must include a data availability statement. This statement should provide the following information, where applicable:

- Accession codes, unique identifiers, or web links for publicly available datasets
- A description of any restrictions on data availability
- For clinical datasets or third party data, please ensure that the statement adheres to our policy

All results generated in this study are available in Supplementary Data.
All new reporter strains generated for this study will be available for order from Caenorhabditis Genetics Center.
The 3D time-lapse image data and cell lineage tracing results used to construct the scCAMERA generated in this study have been deposited in OMIX (Open Archive for Miscellaneous Data), China National Center for Bioinformation, Chinese Academy of Sciences [https://ngdc.cncb.ac.cn/omix/release/OMIX002458].
The raw RNA-seq data are available at the Genome Sequence Archive of the National Genomics Data Center of China [https://ngdc.cncb.ac.cn/gsa/browse/CRA009193].
All processed data generated in this study and their visualization are available at http://dulab.genetics.ac.cn/scCAMERA.

Research involving human participants, their data, or biological material

Policy information about studies with human participants or human data. See also policy information about sex, gender (identity/presentation), and sexual orientation and race, ethnicity and racism.

Reporting on sex and gender	Not applicable
Reporting on race, ethnicity, or other socially relevant groupings	Not applicable
Population characteristics	Not applicable
Recruitment	Not applicable
Ethics oversight	Not applicable

Note that full information on the approval of the study protocol must also be provided in the manuscript.

Field-specific reporting

Please select the one below that is the best fit for your research. If you are not sure, read the appropriate sections before making your selection.

☒ Life sciences ☐ Behavioural & social sciences ☐ Ecological, evolutionary & environmental sciences

For a reference copy of the document with all sections, see nature.com/documents/nr-reporting-summary-flat.pdf

Life sciences study design

All studies must disclose on these points even when the disclosure is negative.

Sample size	The invariant lineage of C. elegans embryogenesis ensures high reproducibility of events. All expressing experiments involving 4D imaging of embryos were performed with at least two replicates. For functional experiments, sample sizes were determined based on previous reports containing similar assays (Milton et al., Dev Biol, 2013; Hughes et al., G3 (Bethesda), 2022; Al-Hashimi et al., G3 (Bethesda), 2019). All sample size numbers were listed in the corresponding figure legends. No statistical method was used to predetermine the sample size.
Data exclusions	No data was excluded.
Replication	Multiple embryos (two or more) were imaged and analyzed for each reporter strain for the embryonic expression of microRNAs. For L1 stage expression, multiple larvae were imaged, and the one that exhibited a representative expression pattern was selected to determine cellular expression. Functional experiments were successfully repeated at least twice to ensure reliability. The exact number of replicates is given in the figure legends. All findings described in this study are reproducible.
Randomization	For all experiments, groups were allocated based on genotype, which was confirmed either by PCR or the expression of relevant fluorescent markers.
Blinding	Investigators were blinded to group allocations for the following experiments: (1) quantifying and comparing pharyngeal pumping rates between animals with different genotypes (Figure 4I), (2) measuring pharynx morphology to determine the genetic interaction between mir-1 and tbx-2 (Figure S11H-J), (3) measuring canal length to demonstrate the genetic interaction between miR-232 and miR-234 in canal development (Figure 6). Other experiments were not blinded because gene expression patterns and mutant phenotypes were easily observable or obtained computationally.

Reporting for specific materials, systems and methods

We require information from authors about some types of materials, experimental systems and methods used in many studies. Here, indicate whether each material, system or method listed is relevant to your study. If you are not sure if a list item applies to your research, read the appropriate section before selecting a response.

Materials & experimental systems

n/a	Involved in the study
☒	☐ Antibodies
☒	☐ Eukaryotic cell lines
☒	☐ Palaeontology and archaeology
☐	☒ Animals and other organisms
☒	☐ Clinical data
☒	☐ Dual use research of concern
☒	☐ Plants

Methods

n/a	Involved in the study
☒	☐ ChIP-seq
☒	☐ Flow cytometry
☒	☐ MRI-based neuroimaging

Animals and other research organisms

Policy information about studies involving animals; ARRIVE guidelines recommended for reporting animal research, and Sex and Gender in Research

Laboratory animals	Caenorhabditis elegans - All C. elegans strains used in this study are detailed in Supplementary Data 1. L4 stage worms were used for crossing. 1-3 day old adult hermaphrodites were used for embryo mounting and L1 stage larvae were used for RNAi treatments. Embryos and L1 stage larvae (sex was not determined) were imaged.
Wild animals	No wild animals were used in this study.
Reporting on sex	Most C. elegans (99.9%) are hermaphrodites, so all findings described in this study apply to hermaphrodite animals.
Field-collected samples	No field-collected samples were used in this study.
Ethics oversight	No ethical approval or guidance was required.

Note that full information on the approval of the study protocol must also be provided in the manuscript.

Plants

Seed stocks	*Report on the source of all seed stocks or other plant material used. If applicable, state the seed stock centre and catalogue number. If plant specimens were collected from the field, describe the collection location, date and sampling procedures.*
Novel plant genotypes	*Describe the methods by which all novel plant genotypes were produced. This includes those generated by transgenic approaches, gene editing, chemical/radiation-based mutagenesis and hybridization. For transgenic lines, describe the transformation method, the number of independent lines analyzed and the generation upon which experiments were performed. For gene-edited lines, describe the editor used, the endogenous sequence targeted for editing, the targeting guide RNA sequence (if applicable) and how the editor was applied.*
Authentication	*Describe any authentication procedures for each seed stock used or novel genotype generated. Describe any experiments used to assess the effect of a mutation and, where applicable, how potential secondary effects (e.g. second site T-DNA insertions, mosiacism, off-target gene editing) were examined.*
